# Interventions to enhance work participation in people with chronic pain: A systematic review and meta‐analysis including analysis of complex psychological intervention components

**DOI:** 10.1111/bjhp.70077

**Published:** 2026-05-08

**Authors:** Joanna McParland, Lorna Booth, Grace Dibben, Ukachukwu Abaraogu, Elaine Wainwright, Evangelia Demou, Lynn Williams, Paul Flowers, Lisa Kidd, Jo Daniels, Hussein Patwa, Paulina Wegrzynek, Sarah Audsley, Ronald O'Kane, Amelia Parchment, Hannah Ranaldi, Karen Walker‐Bone

**Affiliations:** ^1^ School of Health and Life Sciences Glasgow Caledonian University Glasgow UK; ^2^ School of Health and Wellbeing University of Glasgow Glasgow UK; ^3^ School of Health and Life Sciences University of the West of Scotland Paisley UK; ^4^ Institute for Applied Health Sciences University of Aberdeen UK; ^5^ Department of Psychological Sciences and Health University of Strathclyde Glasgow UK; ^6^ Department of Psychology University of Bath Bath UK; ^7^ Patient Partner with Lived Experience of Chronic Pain Aberdeen UK; ^8^ School of Sciences Bath Spa University Bath UK; ^9^ Department of Sport, Exercise and Rehabilitation University of Northumbria Newcastle upon Tyne UK; ^10^ School of Health Sciences University of Manchester Manchester UK; ^11^ Monash Centre for Occupational and Environmental Health University of Monash Melbourne Australia

**Keywords:** behaviour change techniques, chronic pain, intervention functions, meta‐analysis, systematic review, theoretical domains, work outcomes

## Abstract

**Purpose:**

Chronic pain impairs work participation. Psychological interventions can support people with chronic pain to work, yet little is known about which components are most effective. A systematic review and meta‐analysis assessed the effectiveness of interventions targeting sick leave, return to work, work ability and work‐related self‐efficacy in chronic pain populations. Intervention content was analysed to identify effective components.

**Methods:**

A search strategy was developed and applied to six databases from inception until 2nd March 2023, being updated in December 2024: PsychInFO, Medline, Cinahl, Web of Science, Cochrane Library and Embase. Intervention descriptions were coded for intervention functions, theoretical domains and behaviour change techniques. Risk of bias was assessed using the ROB‐2 tool.

**Results:**

51 randomized controlled trials were identified. Study quality was poor overall. Meta‐analysis showed that psychological interventions were complex, that is, contained multiple components delivered alongside other interventions, which together were associated with reduced sick leave (SMD −.41, 95% CI: −.64 to −.18) and a small increase in those working at long‐term follow‐up (>12 months) (RR 1.03, 95% CI: 1.01–1.06; *I* ^2^ = 0%) but not work ability/capacity (SMD −.02, 95% CI: −.12–.08, *I* ^2^ = 0%) or return to work (RR .98, 95% CI: .91–1.05, *I* ^2^ = 0%). No intervention components appeared most effective, but five common components were identified: education, skills/training, social support, emotional regulation, and confidence building.

**Conclusion:**

Complex psychological interventions can positively influence work outcomes for people with chronic pain. Future research should prioritize high‐quality studies and incorporate the five components to enhance work‐focussed support.

**Statement of Contribution**

**What is already known on this subject?**

- Chronic pain contributes to global disability and is associated with work loss and reduced employment.
- Interventions can improve quality of life, functioning, and work outcomes among people with chronic pain.
- It is less clear which intervention components work best to improve work outcomes among people with chronic pain.

**What does this study add?**

- There is poor quality evidence of which interventions improve work participation among people with chronic pain.
- Interventions to improve work outcomes for chronic pain are complex in nature and include five common components:
- Poor reporting and data gaps limit conclusions on effective interventions, notably for specific employee groups.

## INTRODUCTION

Chronic pain, defined as pain in one or more anatomical regions that persists or recurs for longer than 3 months (Nicholas et al., 2019), is a disease in its own right (World Health Organization, 2022) and a major and growing public health concern. At least one third of the UK population self‐report chronic pain, with 14% reporting disabling pain (Fayaz et al., 2016).

Chronic pain substantially affects quality of life, including working life (Patel et al., 2012). Around 49% of disability benefit recipients cannot work, frequently due to chronic pain (Department for Work and Pensions, 2025). For those in employment, work productivity can be reduced (Kawai et al., 2017) and chronic pain is among the leading causes of sickness absence in the UK (Health and Safety Executive, 2024), with 32% of employees off sick with chronic pain absent more than 1 month (Wynne‐Jones et al., 2014). Not working is problematic because high‐quality work benefits physical and psychological wellbeing compared with unemployment (Rueda et al., 2012; Waddell & Burton, 2006; Wainwright et al., 2021) and can reduce health inequalities (Black, 2008). Economically, work participation decline costs the economy several billion pounds (Bevan, 2015).

NICE clinical guidelines (2021) recommend the management of chronic pain should incorporate a biological, psychological, and social framework. Evidence (Demou et al., 2018; Wainwright et al., 2019) shows that approaches including workplace accommodations, service coordination, and providing health services alongside psychological interventions can reduce sick leave and promote return to work. Despite methodological variations and inconsistent research quality (LoMartire et al., 2021; Pike et al., 2016; Wainwright et al., 2019), data suggest psychological interventions can help to improve return‐to‐work outcomes delivered alone (Finnes et al., 2019; Wainwright et al., 2019), or as part of a complex intervention (Cullen et al., 2018; Kamper et al., 2015; Wegrzynek et al., 2020).

Psychological interventions for chronic pain draw on varied theories, from cognitive behavioural to less common psychodynamic approaches. Despite their differences, they share the aim to deepen understanding of pain and build skills to overcome psychological and behavioural barriers to living an active, valued life (Vase et al., 2025). Common components include psychoeducation, self‐efficacy building, problem‐solving, cognitive reframing, behavioural exposure, and strategies to increase engagement in valued activities (Eccleston et al., 2013; Vase et al., 2025; Williams et al., 2020). However, because these interventions are complex and multi‐component, it is currently unclear which components are most effective, particularly for improving work outcomes.

Behaviour change techniques (BCTs) provide a framework for identifying the active ingredients of interventions that influence behaviour by targeting psychological, social, and environmental drivers (Michie et al., 2013). Mapping BCTs helps clarify effective components, standardize intervention reporting, support replication (Abraham et al., 2014; Wood et al., 2016), and link BCTs with theoretical domains (Cane et al., 2012) and broad intervention functions (broad types of activity or strategies that change behaviour) (Michie et al., 2014) to comprehensively understand how interventions work. While a previous review analysed BCTs in interventions for those with musculoskeletal conditions in relation to work outcomes (Palmer et al., 2012), it lacked theoretical analysis and is now outdated.

We conducted a systematic review to synthesize the current evidence on interventions to improve work‐related outcomes among individuals with chronic pain. Our objective was to assess intervention effectiveness and specify intervention components in relation to BCTs, theoretical domains, and intervention categories associated with improved work outcomes in this population.

## MATERIALS AND METHODS

The protocol for this systematic review was prospectively registered on the Prospective Register of Systematic Reviews (PROSPERO) database (ID: CRD42022375328). This article follows the Preferred Reporting Items for Systematic Reviews and Meta‐Analyses (PRISMA) reporting guidelines (Rethlefsen et al., 2021) (Supplementary file 1).

### Information sources and inclusion criteria

The review included randomized controlled trials involving adult participants (age ≥ 18 years) with self‐reported or clinically diagnosed chronic pain lasting ≥3 months. Interventions were eligible if they incorporated psychological components, that is, techniques that target internal processes such as cognition and emotion and how they influence behaviour and experience. This might include techniques building self‐efficacy and confidence, problem‐solving, reframing cognition and behavioural exposure (Vase et al., 2025). Any type of comparator or control condition was included. Work‐related outcomes included return to work following time away, sick leave, presenteeism, work ability and work‐related self‐efficacy. Observational studies, qualitative studies, commentaries, opinion letters, and case studies were excluded.

A systematic search of six electronic databases was conducted without date or language restriction from inception until 2nd March 2023: PsychINFO (Ovid); MEDLINE (via EBSCO); Web of Science (Core Collection); CINAHL (via EBSCO); Cochrane Library (CENTRAL – Cochrane Central Register of Control Trials) and EMBASE (via Ovid, accessed via the NHS Knowledge Network). See Supplementary file 2 for a sample search strategy. An updated search for RCTs was conducted on 9th December 2024. Pain, work, and clinical websites were searched between April 2023–January 2024; the reference lists of included studies were reviewed, and citations of included papers were tracked until 1 March 2024.

### Study selection

Title/abstract and full‐text screening were each undertaken independently by two reviewers using Covidence, and disagreements were discussed with a third reviewer. Non‐English abstracts were processed through Google Translate to determine eligibility. Five papers were translated into English at the full‐text screening stage by a translator. Forty‐two authors were contacted for further information at this stage, with a 45% response rate. The updated search was managed using RefWorks.

### Data extraction

Extracted data included study design, recruitment methods, study characteristics, participants (e.g., age, sex, socio‐economic status, and work characteristics); intervention and comparators and work outcomes. Data extraction was informed by the TIDier framework (Hoffmann et al., 2014) and pilot tested. One reviewer extracted data from each study into a standardized data extraction form which was checked by a second reviewer. Forty‐nine authors were contacted for further information about their intervention at this stage, with a 37% response rate.

### Risk of bias assessment

Two reviewers independently assessed risk of bias (ROB) using the Risk‐Of‐Bias 2 (ROB 2) tool (Higgins et al., 2021). Each assessment focussed on the longest follow‐up period for each outcome. Reviewers met to resolve disagreements.

### GRADE certainty of evidence

The certainty of the evidence was judged as either ‘high’, ‘moderate’, ‘low’ or ‘very low’ based on the five GRADE (Grading of Recommendations, Assessment, Development and Evaluation) considerations of unexplained heterogeneity or inconsistency of results, indirectness of evidence, imprecision of results, and evidence of publication bias (Guyatt et al., 2008). GRADE assessments were conducted independently by two reviewers who met to resolve discrepancies.

### Coding interventions

Verbatim descriptions of interventions and comparator/control conditions were coded for the presence of the 9 intervention functions from the Behaviour Change Wheel Framework (Michie et al., 2014); for the 14 domains within the Theoretical Domains Framework (TDF) (Cane et al., 2012), and for the 93 BCTs across 16 groups within the Behaviour Change Technique Taxonomy (BCTTv1) (Michie et al., 2013). Interventions were coded to give a systematic, theory‐driven account of active components, allowing consistent comparison and clearer identification of mechanisms of effect.

The interventions and control/comparator conditions were coded independently by two reviewers trained in the use of the BCT taxonomy. A third reviewer resolved disagreements. A congruence analysis was conducted to assess alignment among intervention functions, TDF domains, and BCTs within each condition. Three authors independently reviewed the coding for each intervention and control/comparator condition, applying their expertise and the Behaviour Change Wheel framework (Michie et al., 2014) to assign a score: 1 = low, 2 = moderate, 3 = high congruence. Final scores were based on agreement between at least two of the three authors.

### Data synthesis and statistical analysis

Study, participant, and intervention characteristics were summarized descriptively, and intervention component differences were tested using two‐tailed independent *t*‐tests. Meta‐analysis was conducted only where appropriate, that is, interventions, participants, and outcomes were similar enough for pooling. Where necessary, reported outcomes were transformed using conversion formulae recommended in the Cochrane handbook (Higgins et al., 2024). Given the heterogeneity across included studies, we used the DerSimonian and Laird random‐effects model, which assumes that each study estimates a different underlying intervention effect. Dichotomous outcomes (e.g., work status, return to work) were expressed as risk ratios (RR) with 95% confidence intervals (CI). Continuous outcomes (e.g., sick leave and work capacity) were measured and reported in different ways and were therefore pooled using standardized mean differences (SMD) with 95% CI. Effect sizes were interpreted using Cohen's *d*. Primary analyses were based on outcome data pooled at the longest reported follow‐up.

Statistical heterogeneity was assessed using the *I* ^2^ statistic, with *I* ^2^ > 50% considered to indicate substantial heterogeneity. For meta‐analyses with ≥10 comparisons, we visually inspected funnel plots and used Egger's test to examine potential publication bias (Page et al., 2024).

We investigated heterogeneity and pre‐specified intervention effect modifiers using the following approaches: (1) Univariate random‐effects meta‐regression to explore: (a) inclusion of individual intervention components (intervention functions, theoretical domains and BCTs), (b) type of professionals involved in intervention delivery; and (2) stratified subgroup meta‐analyses to explore: (a) risk of bias, (b) follow‐up duration (0–3 months, 3–6 months, 6–12 months and >12 months), (c) mode of intervention delivery (individual, group or a combination of both – hereafter referred to as hybrid), and (d) presence of a clearly identifiable aspect of work within the interventions, such as ergonomics support. Given the large number of statistical tests performed in this review, interpretation was based on 95% CIs rather than *p*‐values. Analyses were performed in STATA version 18. Two sensitivity analyses were conducted to assess the robustness of primary analysis results, first by removing an outlying study reporting sick leave data, and second to include work capacity data reported in two studies identified in the updated searches.

Additional outcomes (presenteeism, work‐related self‐efficacy) were narratively synthesized using a vote counting approach (Higgins et al., 2022), summarizing the number of statistically significant results in favour of the intervention, in favour of the control or comparator, or indicating no difference between groups.

### Patient and public involvement

A researcher with lived experience of chronic pain co‐chaired the Project Advisory Group (PAG), which included two others with lived experience, alongside employers, clinicians, and policy experts. The group monitored review methods, supported interpretation of findings, and advised on dissemination. Impact was tracked using the PIRIT tool (Newman et al., 2023) and qualitative feedback, reported per GRIPP 2 guidelines (Staniszewska et al., 2017).

## RESULTS

### Study selection

**FIGURE 1:**
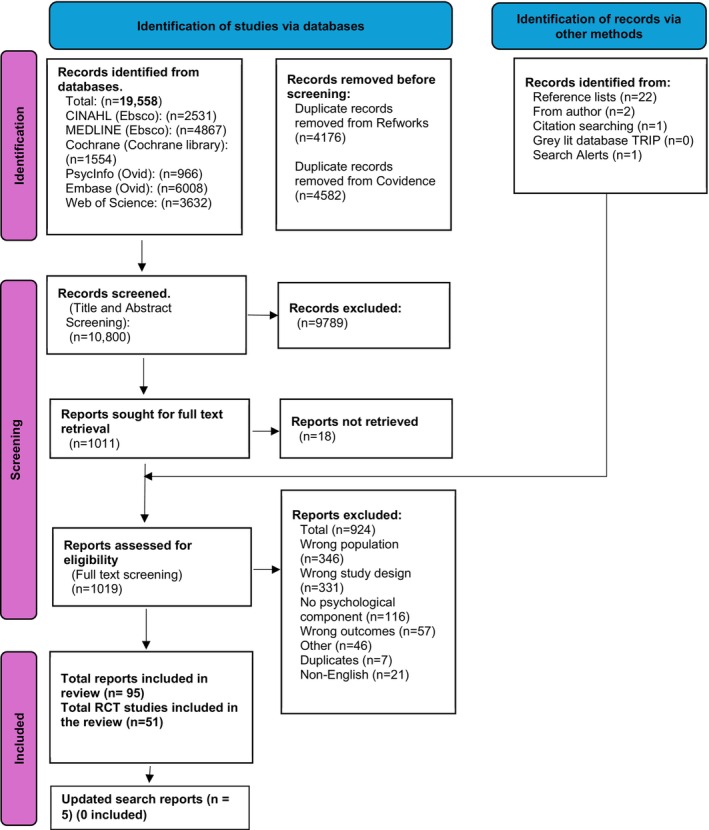
PRISMA flow diagram.

Overall, 19,558 records were identified through database searching. After de‐duplication, 10,800 title/abstracts were screened. Of 1011 records eligible for full‐text screening, 18 could not be accessed. Following grey literature searching which identified 26 records, 1019 records underwent full‐text screening, and following the exclusion of 924 papers (Supplementary file 3 Table of excluded studies.xlsx), 51 RCTs were included (Figure 1). See supplementary file 4 for the linked RCT papers.

### Study characteristics

Most studies were conducted in Europe (*n* = 44, 86%) and the rest (*n* = 7, 14%) in North America. Parallel RCTs were most common (*n* = 45, 88%), followed by pilot RCTs (*n* = 3, 6%), factorial RCTs (*n* = 2, 4%) and one crossover RCT (2%). The majority of studies (*n* = 38, 75%) had 2 study arms (usually one intervention and a control), while the rest (*n* = 13, 25%) had multiple intervention and/or control arms (Table 1).

**TABLE 1:** RCT study characteristics.

Author/linked papers	Design	Country	Participants Total randomized/total who took part	Arms (*N*)	Follow‐up time periods	Participant demographics (mean) age: (in years) % female: SES:	Recruitment	Pain condition	Pain duration	Employment status	Sick leave at baseline	Sick leave category
Alaranta et al. (1994)	Parallel RCT	Finland	293/293	2	3 months 12 months	Age: 40.4 to 40.5. Female: 55%. SES: Leading position/entrepreneurs 8/13%: White collar 31/30%; Blue collar 61/57%	Insurance/health records	Chronic low back pain	>6 months	100% Employed	Mean 57.8–58.5 days during previous 12 months	Long‐term sick leave
Altmaier et al. (1992)	Parallel RCT	USA	47/45	2	6 months	Age: 39.9. Female: 26%. SES: Mean Duncan SEI score for occupation 24.18–25.65	Medical setting	Chronic low back pain	NR	NR	>3–30 months	Long‐term sick leave
Andersen et al. (2015)/Anderson et al. (2013, 2016)	Parallel RCT	Denmark	141/140	3	3 months 11 months	Age: 45.2. Female: 55%. SES: No education (18%); minimal further education (50%); higher education (30%)	Insurance/health records	Chronic back or upper body	>3 months	Employed and unemployed (<50% unemployed)	<9 weeks	Short‐ and long‐term sick leave
Baumeister et al. (2021)/Lin et al. (2017)	Parallel RCT	Germany	210/210	2	9 weeks 6 months	Age: 49.9. Female: 60%. SES: High education (14%), medium (22%), low (63%)	Medical setting	Chronic back pain	>6 months	NR	NR	NR
Bendix et al. (1995)/Bendix et al. (1997)	Parallel RCT	Denmark	138/127	3	4 months 1 year 2 years 5 years	Age: 40–41. Female: 62%. SES: NR	Medical setting	Chronic low back pain	>6 months	Employed and unemployed	Median sick leave during previous 3 years = 180	Long‐term sick leave
Bendix et al. (1996)/Bendix et al. (1998a, 1998b)	Parallel RCT	Denmark	106/106	2	4 months, 2 years 5 years	Age: 40 to 41. Female: 62%. SES: NR	Medical setting	Chronic low back pain	>6 months	Employed and unemployed	Median sick leave during previous 3 years 340–370 days	Long‐term sick leave
Bendix et al. (2000)	Parallel RCT	Denmark	132/125	2	12 months	Age: 40 to 44. Female: 60%. SES: NR	Medical setting	Chronic low back pain	>3 months	Employed and unemployed (<50% unemployed)	Median sick leave during previous 3 years: 296–400	Long‐term sick leave
Brendbekken et al. (2017)/Brendbekken et al. (2016, 2018), Hagen et al. (2000), Indahl et al. (1995)	Parallel RCT	Norway	284/284	2	3 months 12 months 24 months	Age: 40.9 to 41.6. Female: 54%. SES: Public school 1–12 years (69%); university college >12 years (18%)	Medical setting	Musculoskeletal pain	>3 months	100% Employed	Mean SL 147 days (previous 8 months)	Long‐term sick leave
Brox et al. (2003)/Brox et al. (2010), Froholdt et al. (2012)	Parallel RCT	Norway	64/64	2	12 months 4 years 9 years 11 years	Age: 42.4 to 44.1. Female: 61%. SES: Primary school (41%), High school (27%), Uni/college (33%)	Medical setting	Chronic low back pain	>1 year	Employed (<50% unemployed)	NR	NR
Brox et al. (2006)/Brox et al. (2010), Froholdt et al. (2012)	Parallel RCT	Norway	60/60	2	12 months 4 years 9 years 11 years	Age: 42 to 43. Female: 48%. SES: High school/university/college (33%)	Medical setting	Chronic low back pain	>1 year	<50% unemployed	NR	Short‐term sick leave
Calner et al. (2017)/Protocol ID: NCT01475591	Parallel RCT	Sweden	109/109	2	4 months 12 months	Age: 42 to 44. Female: 77%. SES: NR	Medical setting	Musculoskeletal	78 to 79 months	Employed and unemployed (<50% unemployed)	NR	Short‐term sick leave
den Hollander et al. (2018)/den Hollander et al. (2016)Protocol ID: NCT00625976	Parallel RCT	The Netherlands	46/38	2	6‐months after treatment	Age: 44.85. Female: 70%. SES: Low (63%), middle (29%), high (13%)	NR	Complex regional pain syndrome‐I	5.1 years	Employed and unemployed (>50% unemployed)	NR	Short‐ and long‐term sick leave
Ehrenborg and Archenholtz (2010)/Ehrenborg et al. (2014)	Parallel RCT	Sweden	65/62	2	6 months	Age: 39.4. Female: 51%. SES: NR	Hospital/rehabilitation	Whiplash	25 months	Employed and unemployed	NR	Long‐term sick leave
Friedrich et al. (1998)/Friedrich et al. (2005)	Parallel RCT	Austria	98/93	2	3.5 weeks 4 months 12 months 5 years	Age: 43.3–44.9. F: 48%. SES: NR	Medical setting	Chronic low back pain	46.1–50.64 months	Employed	NR	NR
Gustavsson and von Koch (2006)	Pilot RCT	Sweden	37/37	2	5 months	Age: 36–43. Female: 76%. SES: NR	Medical setting	Neck pain	>3 months	NR	Median sick leave during previous 3 months 0–23 days	Short‐term sick leave
Hampel et al. (2019)/Protocol ID: DRKS00015465; Kopnick et al. (2020); Neumann and Hampel (2022)	Crossover RCT	Germany	1306/1306	2	3 months 6 months 12 months 24 months	Age: 53.3. Female: 36%. SES: Low (20%), middle (48%), high (30%)	Medical setting	Chronic low back pain	>6 months	Employed and unemployed (<50% unemployed)	>2 weeks in previous 3 months (int: *n* = 148, 50%; con: *n* = 133,46%)	Short‐ and long‐term sick leave
Härkäpää et al. (1990)/Härkäpää et al. (1989)	Parallel RCT	Finland	459/459	3	1.5 years. 2.5 years. 4.5 years	Age: 44.8–45.2. Female: 37%. SES: Basic education <9 years (93%), >9 years (7%), vocational education (52%)	Population level	Chronic low back pain	14.2–14.6 years	Employed (100% employed)	Average sick leave during previous 2 years 31.3–37.7 days	Short‐term sick leave
Haugli et al. (2000)/Haughli et al. (2001, 2003)	Parallel RCT	Norway	174/174	2	1 month 12 months	Age: 41–45. Female: 95%. SES: Education >12 years (17%)	Insurance/health records	Musculoskeletal	6–9 years	100% Employed	Average sick leave during previous 6 months 87–108 days	Long‐term sick leave
Haugmark et al. (2021)/Haughmark et al. (2018)	Parallel RCT	Norway	170/170	2	3 months 12 months	Age: 41–44. Female: 94%. SES: Primary/middle school (12%), upper secondary/vocational (40%), bachelor/university (48%)	Medical setting	Fibro‐myalgia	8 years	Employed and unemployed (<50% unemployed)	NR	NR
Hutting et al. (2015)/Hutting et al. (2013)	Parallel RCT	The Netherlands	123/123	2	3 months 6 months 12 months	Age: 45–47.7. Female: 72%. SES: Lower general (42%), higher professional (33%), academic higher (25%)	Population level	CANS	3 months to 10 years	100% Employed	NR	Long‐term sick leave
Jensen et al. (1995)	Parallel RCT	Sweden	66/66	2	6 months 12 18 months	Age: 39–40. Female: 65%. SES: NR	Medical Setting	Neck and shoulder	NR	100% Employed	Average sick leave during previous year 237–256 days	Long‐term sick leave
Jensen et al. (1997)	Parallel RCT	Sweden	63/63	2	6 months 12 months 18 months	Age: 43–45. Female: 100%. SES: Education <10 years (37%), 10–13 years (52%), ≥14 years (11%)	Insurance/health records	Non‐specific spinal pain	44–51 months	100% Employed	Average sick leave during previous year 73–84 days	Long‐term sick leave
Jensen et al. (2001)/Jensen et al. (2005)	Factorial RCT	Sweden	214/214	4	3 months 6 months 9 months 12 months 15 months 18 months 3 years	Age: 42.5–43.9. Female: 55%. SES: Compulsory school (58%), high school (33%), post‐high school (10%)	Insurance/health records	Non‐specific spinal pain	22–27.3 months	Employed and unemployed (<50% unemployed)	Average sick leave during previous year: 135–162	Long‐term sick leave
Johansson et al. (1998)	Parallel RCT	Sweden	42/42	2	1 month	Age: 43.5. Female: 86%. SES: Elementary school only (28%), higher education (8%)	Medical setting	Musculoskeletal	11 years (SD 6.3)	Employed and unemployed (<50% unemployed)	Level of sick leave: 51.7%–84.3%	Short‐term and long‐term sick leave
Kaapa et al. (2006)	Parallel RCT	Finland	130/120	2	8 weeks 6 months 12 months 24 months	Age: 46–46.5. Female: 100%. SES: NR	Medical setting	Chronic low back pain with or without sciatica	14–16 months	100% Employed	<90 days during previous year	Short‐term and long‐term sick leave
Kool et al. (2005)/Kool et al. (2007), Oesch et al. (2006)	Parallel RCT	Switzerland	174/174	2	3 weeks 3 months 1 year.	Age: 41.6–42.5. Female: 79%. SES: No professional education (46%)	NR	Chronic low back pain	>3 months	Employed and unemployed (<50% unemployed)	Average sick leave during previous 2 years: 184–199	Long‐term sick leave
Lambeek et al. (2010)/Lambeek et al. (2007)	Parallel RCT	The Netherlands	134/134	2	12 months	Age: 45.5–46.8. Female: 42%. SES: Education Low (28%), Intermediate (49%), High (23%)	Medical setting	Chronic low back pain	>3 months	100% Employed	Median sick leave (unspecified window): 142–163	Long‐term sick leave
Lemstra et al. (2002)	Parallel RCT	Canada	80/80	2	6 weeks 3 months	Age: 33.2–35.6. Female: 66%. SES: Less than high school (5%), high school graduate (60%), some university or college (35%)	Medical setting	Migraine	101.67–102.91 (months)	Employed and unemployed (<50% unemployed)	NR	NR
Lemstra and Olszynski (2005)	Parallel RCT	Canada	79/79	2	15 months	Age: 49.1–49.7. Female: 85%. SES: Less than high school (9%), high school graduate (48%), university/college (43%)	Medical setting	Fibro‐myalgia	120.64–121.70 months	Employed and unemployed (<50% unemployed)	NR	NR
Lindell et al. (2008)	Parallel RCT	Sweden	125/125	2	6 months 12 months 18 months	Age: 42.2–43. Female: 54%. SES: NR	Medical setting	Back and neck	>3 months	Employed and unemployed (<50% unemployed)	Average sick leave during previous 18 months: 222–223	Long‐term sick leave
Lindh et al. (1997)	Parallel RCT	Sweden	611/464	2	12 months 3 years 5 years	Age: 39–40. Female: 65%. SES: NR	Insurance/health records	Mixed (Musculoskeletal, fibro‐myalgia, neck, shoulder, back pain)	>3 months	Employed and unemployed (<50% unemployed)	Average sick leave during previous 2 years: 77–80	Long‐term sick leave
Linton et al. (1997)	Parallel RCT	Sweden	113/103	3	12 months	Age: 50–53. Female: 67%. SES: NR	Insurance/health records	Musculoskeletal	26–43 months	100% Employed	Average sick leave during previous 2 years 76.4–92	Long‐term sick leave
Magnussen et al. (2007)	Parallel RCT	Norway	89/89	2	12 months	Age: 49–49.1. Female: 63%. SES: High school or less (<12 years) (78%), college/university (>12 years) (16%)	Insurance/health records	Chronic back pain	9.8–11.6 years	100% unemployed	NR	NR
Marhold et al. (2001)	Parallel RCT	Sweden	72/72	4	4 months 6 months	Age: 46. Female: 100%. SES: Compulsory school (61%), high school (25%), university degree (14%)	Insurance/health records	Musculoskeletal	10–48 months	100% Employed	Average sick leave over 2 months 52.6–57.4	Long‐term sick leave
McKnight et al. (2010)/Going (2012)	Parallel RCT	USA	273/269	3	9 months 24 months	Age: 51.9–53.3. Female: 76%. SES: College educated (63%)	Population level	Knee osteo‐arthritis	NR	NR	>4 months during year prior	Long‐term sick leave
Meyer et al. (2005)	Pilot RCT	Switzerland	33/33	2	8 weeks 32 weeks	Age: 42–44. Female: 21%. SES: No professional education (61%)	Medical setting	Non‐specific pain	36 months	100% Employed	Median duration of work incapacity 5.6–7 months	Long‐term sick leave
Miller et al. (2020)/Miller et al. (2015a, 2015b)	Parallel RCT	Canada	102/102	2	1 week 3 months‐	Age: 52.2–53.4. Female: 74%. SES: Less than high school (24%), high school (51%), university (26%)	Medical setting	Non‐cancer pain	120 months	>50% unemployed	NR	NR
Mitchell and Carmen (1994)	Parallel RCT	Canada	542/542	2	12 months 24 months	Age: <45 (*n* = 348); >45 (*n* = 194). Female: 29%. SES: NR	Population level	Soft tissue or back injury	NR	100% Employed	NR	NR
Pach et al. (2022)/Blodt et al. (2014)	Parallel RCT	Germany	220/220	2	3 months 6 months	Age: 37.9–39.8. Female: 70%. SES: ≥10 years education (70%)	Population level	Neck	79.2–86.4 months	NR	Average sick leave days (unspecified period) 1.7–2.1	Short‐term sick leave
Pato et al. (2010)	Factorial RCT	Switzerland	87/87	6	3 months 6 months	Age: 40.5. Female: 62%. SES: NR	Insurance/health records	Whiplash	6–12 months	Employed and unemployed	NR (<50% unemployed)	NR
Reme et al. (2016)/Harris et al. (2017), Reme et al. (2007, 2011)	Parallel RCT	Norway	529/529	6	3 months 6 months 12 months	Age: 42.9–45.5. Female: 51%. SES: Primary school (1–12 years) (61%), university/college (29%), other (6%)	Insurance/health records	Chronic low back pain	12.5 years	100% Employed	2–10 months	Long‐term sick leave
Rolving et al. (2015)/Rolving et al. (2014)	Parallel RCT	Denmark	96/96	2	3 months 6 months 12 months	Age: 47.7–51.4. Female: 53%. SES: NR	Medical setting	Lumbar spinal	NR	Employed and unemployed (<50% unemployed)	NR	NR
Rolving et al. (2022)/Protocol ID: NCT03141541, Skovbo et al. (2021)	Parallel RCT	Denmark	136/136	2	6 months 12 months	Age: 41.7–46.1. Female: 71%. SES: No further education (24%), <3 years (47%), 3–4 years (26%), >4 years (2%)	Medical setting	Chronic low back pain	>3 months	Employed and unemployed (<50% unemployed)	Sick leave in previous 4 weeks: 1–4 days (*n* = 25); 5–7 days (*n* = 0)	Short‐term sick leave
Sander et al. (2020)/Sander et al. (2017)	Parallel RCT	Germany	295/295	2	9 weeks 6 months and 12 months	Age: 51.7–53.9. Female: 62%. SES: Low (70%), medium (14.6%), high (15.3%)	Medical setting	Chronic back pain	>6 months	NR	NR	NR
Schlicker et al. (2020)/Protocol ID: DRKS00010820	Pilot RCT	Germany	76/76	2	9 weeks 6 months	Age: 50.1–51.3. Female: 72%. SES: Education, Low (11%), medium (67%), high (22%)	Insurance/health records	Chronic back pain	>3 months	100% Employed	>1 week to <6 months	Short and long‐term sick leave
Schweikert et al. (2006)	Parallel RCT	Germany	409/409	2	6 months	Age: 46.6–46.9. Female: 8%. SES: NR	Insurance/health records	Chronic low back pain	>6 months	Employed and unemployed	N % sick listed <6 months in previous year: 283 (69.2%); >6 months 23 (5.6%)	Short‐term and long‐term sick leave
Skouen et al. (2002)/Haldorsen et al. (2002), Skouen and Kvale(2009)	Parallel RCT	Norway	211/195	3	12 months 18 months 26 months	Age: 42.9–44. Female: 60%. SES: Average annual earning approximately US $28,000	Insurance/health records	Chronic low back pain	>3 months	100% Employed	3 months average	Long‐term sick leave
Skouen et al. (2006)	Parallel RCT	Norway	215/208	3	54 months	Age: 42.6–43.2. Female: 67%. SES: NR	Insurance/health records	Chronic widespread pain	>3 months	100% Employed	3 months average	Long‐term sick leave
Smeets et al. (2009)	Parallel RCT	The Netherlands	223/223	4	26 weeks 52 weeks.	Age: 41.5–43. Female: 45%. SES: Low (63.1%), middle‐high (36.9%)	Medical setting	Chronic low back pain	61.6–73.2 months	Employed and unemployed (<50% unemployed)	NR	NR
van Eijk‐Hustings et al. (2013)/Kroese (2006)	Parallel RCT	The Netherlands	203/203	3	18 months	Age: 41.6–43.9. Female: 96%. SES: Education, low (53%), medium (33%), high (13%)	Medical setting	Fibro‐myalgia	6.2–7.3 month	Employed and unemployed (<50% unemployed)	NR	Short‐term sick leave
Von Korff et al. (2005)	Parallel RCT	USA	240/240	2	2 Months 6 Months 12 Months 24 Months	Age: 49.7–49.8. Female: 63%. SES: Education, ≤12 years (7%), some college (44%), college graduate (49%)	Insurance/health records	Chronic back pain	>3 months	Employed and unemployed (<50% unemployed)	NR	Short‐term and long‐term sick leave

### Participant characteristics

Overall, 9944 participants were randomized, 56% of whom were female, and mean ages ranged from 33.2 to 53.9 years. Most participants had back pain (*n* = 46, 91%). Average pain duration ranged from 10 months to 15 years (reported in 27 studies, 53%), and 19 studies (37%) reported minimum pain durations >3 months (*n* = 12, 63%); >6 months (*n* = 6, 32%) or 6–12 months (*n* = 1, 5%) and 5 studies (10%) did not report pain duration. Employed participants were included in 18 studies (35%), 21 (60%) had a mix of employed and unemployed participants, and 1 (2%) included only unemployed participants, while 11 studies did not report employment status. Twenty‐two studies (43%) involved participants who were on long‐term sick leave (≥30 days/year), 8 (16%) on short‐term sick leave (<30 days/year), 8 (16%) included a mix of both, and 13 (25%) did not report this information.

Nine studies (18%) reported the type of work undertaken by participants. With one exception (Hutting et al., 2015), which included a managerial sample, type of work involved manual/blue collar work (Härkäpää et al., 1990; Jensen et al., 1995, 1997; Kaapa et al., 2006; Lindell et al., 2008; Lindh et al., 1997; Marhold et al., 2001; Meyer et al., 2005).

### Intervention characteristics

There were 124 study arms across the 51 RCTs: 74 (60%) were intervention arms including psychological components, while 50 (40%) were comparator arms or controls that did not contain a psychological component. Interventions reporting a psychological component were complex in nature, that is, contained several interacting components (Medical Research Council, 2021), and often included components of cognitive behavioural therapy delivered alongside exercise and physiotherapy, medical treatment and/or health and lifestyle guidance. The interventions mostly targeted pain management, although 20 studies (39%, 24 study arms) involved some clearly identifiable aspect of work (Table 2), commonly ergonomics support. See Supplementary file 5 for intervention content details.

**TABLE 2:** RCT intervention characteristics.

Lead author	Intervention work component (yes/no)	Intervention duration	Who delivered the intervention	Individual or group intervention	Intervention delivery (in‐person or remote)	Intervention location
Alaranta et al. (1994)	No	3 weeks	Psychology professional, physiotherapist, OT, Social worker, health professionals, other	NR	Face‐to‐face	Medical/healthcare setting
Altmaier et al. (1992)	Yes	3 weeks	NR	Intervention 1 = group and individual. Intervention 2 = group.	Face‐to‐face	Medical/healthcare setting
Andersen et al. (2015)	No	6 weeks	Persons with lived experience, other.	Group	Face‐to‐face	Medical/healthcare setting
Baumeister et al. (2021)	No	6 weeks	Psychology professional, health professionals	Individual	Intervention: Remote Control: NR	Home
Bendix et al. (1995)	Yes	6 weeks	Both interventions: Psychology professionals, Physiotherapists, OT, Social worker, Health Professionals, Other	Intervention 1IMT = Group and individual. Intervention 2 PPP = group; Comparator: Group	Face‐to‐face	Medical/healthcare setting
Bendix et al. (1996)	Yes	3 weeks	Psychology professionals, Physiotherapists, OT, Social worker, Health Professionals, Other	Group	Face‐to‐face	Medical/healthcare setting
Bendix et al. (2000)	Yes	8 weeks	Psychology professional, Physiotherapists, OT, Social worker, Health professionals	Individual and group	Face‐to‐face	Medical/healthcare setting
Brendbekken et al. (2017)	Yes	3 months	Intervention 1 (MI)13:47 Physiotherapists, Social Worker, Health Professionals. Intervention 2 (BI): Physiotherapists, Health Professionals	Individual	Face‐to‐face	Medical/healthcare setting
Brox et al. (2003)	No	5 weeks	Physiotherapists	Group and individual	Face‐to‐face	Medical/healthcare setting
Brox et al. (2006)	No	5 weeks	Physiotherapists	Group and individual	Face‐to‐face	Medical/healthcare setting
Calner et al. (2017)	Yes	16 weeks	Both Interventions: Psychology professionals, Physiotherapists, OT, Health Professionals, Other	Individual and group	Intervention 1 = face‐to‐face and remote. Intervention 2 = face‐to‐face.	NR
den Hollander et al. (2018)	No	17 weeks	Psychology professionals, Physiotherapists, OT	Individual	Face‐to‐face	Medical/healthcare setting
Ehrenborg and Archenholtz (2010)	Yes	4–6 weeks	Both interventions: Psychology professionals, Physiotherapists, OT, Social worker, Health Professionals	NR	Face‐to‐face	Medical/healthcare setting
Friedrich et al. (1998)	No	5 weeks	Psychology professionals, Physiotherapists	NR	NR	NR
Gustavsson and von Koch (2006)	No	7 weeks	Physiotherapists	Group and individual	Face‐to‐face	NR
Hampel et al. (2019)	No	4 weeks	Both interventions: Psychology professionals	Group	Face‐to‐face	Medical/healthcare setting
Härkäpää et al. (1990)	No	5 weeks	Both interventions: Psychology professionals, Physiotherapists, Health Professionals	Group	Face‐to‐face	Workplace and medical setting
Haugli et al. (2000)	No	8 months	Physiotherapists, Health Professionals	Intervention: Group Control: individual	Face‐to‐face	NR
Haugmark et al. (2021)	No	22 weeks	Physiotherapists, Health Professionals	Group and individual	Face‐to‐face	Community
Hutting et al. (2015)	Yes	6 weeks	Physiotherapists	Intervention: Group and individual Control: TAU	Face‐to ‐face and remote.	NR
Jensen et al. (1995)	No	5 weeks	Psychology professionals, Physiotherapists, Health Professionals	Group	Face‐to‐face	Medical/healthcare setting
Jensen et al. (1997)	No	5 weeks	Both interventions: Psychology professionals, Physiotherapists, Health Professionals	Group	Face‐to‐face	Medical/healthcare setting
Jensen et al. (2001)	No	4 weeks	Both Interventions: Psychology professionals, Physiotherapists, Health Professionals	Intervention: Group Control: NR	Interventions: Face‐to‐face Control: NR	Medical/healthcare setting
Johansson et al. (1998)	Yes	4 weeks	Psychology professionals, Physiotherapists, OT, Health Professionals, Other	Individual and group	Face‐to‐face	Medical/healthcare setting
Kaapa et al. (2006)	Yes	6–8 weeks	Psychology professionals, Physiotherapists, OT, Health Professionals	Intervention: Group Comparator: Individual	Face‐to‐face	Medical/healthcare setting
Kool et al. (2005)	Yes	3 weeks	Both Interventions: Psychology professionals, Physiotherapists, OT, Social Workers, Health Professionals	NR	Face‐to‐face	Medical/healthcare setting
Lambeek et al. (2010)	Yes	3 months	Physiotherapists, OT, Health Professionals	Group and individual	Face‐to‐face	Workplace and medical setting
Lemstra et al. (2002)	No	6 weeks	Psychology professionals, Physiotherapists, Health Professionals, Other	Intervention: Group and individual Control: Treatment as usual	Face‐to‐face	Community
Lemstra and Olszynski (2005)	No	6 weeks	Psychology professionals, Physiotherapists, Health Professionals, Other	Group and individual	Face‐to‐face	Community
Lindell et al. (2008)	Yes	8 weeks	Psychology professionals, Physiotherapists, Social Worker, Health Professionals	NR	Face‐to‐face	Medical/healthcare setting
Lindh et al. (1997)	Yes	NR	Psychology professionals, Physiotherapists, OT, Social Worker, Other	Intervention: Individual and group Control: NR	Face‐to‐face	Medical/healthcare setting
Linton et al. (1997)	No	12 months	Intervention 1 Educational support group: Persons with lived experience. Intervention 2 Professional Support Group: Other	Intervention: Group Control: Individual	Face‐to‐face	Medical/healthcare setting
Magnussen et al. (2007)	Yes	3–5 days	Health Professionals, Other	Intervention: Individual and group Control: NR	Face‐to‐face	NR
Marhold et al. (2001)	Yes	12 weeks	Both Interventions, Psychology Professionals	Individual	Face‐to‐face	Medical/healthcare setting
McKnight et al. (2010)	No	24 months	Intervention 1: Health Professionals, Intervention 2: Physiotherapists, Health Professionals	NR	Face‐to‐face and remote	Community
Meyer et al. (2005)	Yes	8 weeks	Psychology professionals, Physiotherapists, OT, Social Worker, Health Professionals	Intervention: Group Comparator: NR	Face‐to‐face	Medical/healthcare setting
Miller et al. (2020)	No	6 weeks	Physiotherapists	Intervention: Group Control: individual	Face‐to‐face	Medical/healthcare setting
Mitchell and Carmen (1994)	No	8 weeks	NR	Group and individual	Face‐to‐face	Medical/healthcare setting
Pach et al. (2022)	No	6 months	Not applicable ‐remote intervention	Individual	Intervention: Remote Control: Face‐to‐face	Home
Pato et al. (2010)	No	8 weeks	Intervention 1 (CBT and Infiltration) and Intervention 3 (CBT and Medication): Psychology Professional, Health Professionals. Intervention 2 (CBT plus physio): Psychology Professional, Physiotherapists	Individual	Face‐to‐face	Medical/healthcare setting
Reme et al. (2016)	No	2–3 months	Intervention 1, 5 and 6: Physiotherapists, Health Professionals. Intervention 2, 3, and 4: Psychology Professional, Physiotherapists, Health Professionals	Individual and group	Face‐to‐face	Medical/healthcare setting
Rolving et al. (2015)	No	6 months	Psychology professionals, Physiotherapists, OT, Social Worker, Persons with lived experience. Health Professionals	Group and individual	Face‐to‐face	Medical/healthcare setting
Rolving et al. (2022)	No	3 months	Psychology professionals, Physiotherapists, Health Professionals	Group and individual	Face‐to‐face	Community and healthcare setting
Sander et al. (2020)	No	6 weeks‐4 months	Psychology Professional	Individual	Intervention: Remote Control: NR	Home
Schlicker et al. (2020)	Yes	7‐12 weeks	Psychology Professional	Individual	Remote	Home
Schweikert et al. (2006)	No	3 weeks	Psychology Professional, Health Professional	Group and individual	Face‐to‐face	Medical/healthcare setting
Skouen et al. (2002)	Yes	10 months	Both interventions: Psychology Professional, Physiotherapists, Health Professionals	Intervention 1 Light multidisciplinary: Group. Intervention 2: Extensive multidisciplinary (Intervention): Group and Individual	Face‐to‐face	Medical/healthcare setting
Skouen et al. (2006)	Yes	10 months	Both interventions: Psychology Professional, Physiotherapists, Health Professionals	Intervention 1 Light multidisciplinary: Group. Intervention 2: Extensive multidisciplinary (Intervention). Group and Individual. Control: Individual	Face‐to‐face	Medical/healthcare setting
Smeets et al. (2009)	No	10 weeks	Intervention 1 (GAP): Psychology professional, Social Worker. Intervention 2 (Combined): Psychology professional, Physiotherapists, Social Worker	Group and individual	Face‐to‐face	Medical/healthcare setting
van Eijk‐Hustings et al. (2013)	No	13 months	Physiotherapists, OT	Group and individual	Face‐to‐face	Medical/healthcare setting
Von Korff et al. (2005)	No	34 days	Psychology Professional, Physiotherapists	Individual	Face‐to‐face	Medical/healthcare setting

The interventions including psychological components had a median duration of 8 weeks (interquartile range [IQR] 5–22 weeks), with follow‐up up to 11 years (median 12 months). Most interventions were delivered in healthcare settings by professionals (e.g., psychologists, physiotherapists). Delivery was typically in‐person, either individually (*n* = 17, 23%), in groups (*n* = 23, 31%) or combined group and individual formats (*n* = 27, 36%). Interventions reporting an aspect of work were more frequently delivered by occupational therapists (14/24, 58% vs. 8/46, 17%) and social workers (10/24, 42% vs. 5/46, 11%) than interventions not involving work‐focussed components (Table 2).

### Intervention function, theoretical domain, and BCT intervention components

Compared with comparator/control groups, interventions with psychological components contained significantly more intervention functions (mean 3.8 ± 1.0 vs. 1.3 ± 1.0, *p* < .001); theoretical domains (mean 6.0 ± 1.9 vs. 1.1 ± 1.5, *p* < .005), and BCTs (mean 9.7 ± 4.0 vs. 2.3 ± 2.3, *p* < .001). Interventions with psychological components included 7 of 9 intervention functions, 12 of 14 theoretical domains and 49 of 93 BCTs.

Commonly included intervention functions were education, enablement, training, and persuasion (Supplementary file 6). Common TDF domains were knowledge, skills, emotion, beliefs about capabilities, and social influences. Common BCTs included instruction on performing behaviour, behavioural rehearsal and demonstration of behaviour, social support, credible source, reducing negative emotions, problem‐solving, and body changes. Unique within work‐focussed interventions were environmental restructuring components of TDF domains (7/26, 27% vs. 4/48, 8%) and BCTs (5/26, 19% vs. 0/48, 0%).

Good congruence between the intervention functions, TDF domains and BCTs was identified across 44/124 arms, with some congruence across 54/124 arms (79% overall (Supplementary file 5)). Poor reporting of interventions restricted coding ability in the remaining arms (*n* = 26, 21%). We mapped the most common interventions functions, TDF domains and BCTs against each other (Michie et al., 2014) and identified five common and clear content areas across interventions. These were knowledge/education; skills/training; social support; reducing negative emotions and promoting beliefs about capabilities (Supplementary file 7).

### Work outcomes

Across the 51 RCTs, 91 measures of work participation were identified, with significant measurement heterogeneity. Thirty‐nine measures (43%) were self‐reported; 19 (21%) came from registry data; three studies (3%) had a mix of both and 30 (33%) were reported without clear methodology. Forty (44%) were primary outcomes; 38 (42%) were secondary outcomes, and 13 (14%) were undefined. For meta‐analysis, outcomes were grouped into: work status (e.g., in employment, retirement, disability pension vs. out of work after interventions) (*n* = 24, 40%); return to work (e.g., resuming work following sick leave) (*n* = 9, 15%); sick leave (e.g., number of absent days) (*n* = 14, 23%); or work ability/capacity (e.g., work ability scales, working ability or work impairment) (*n* = 13, 22%).

### Risk of bias

**FIGURE 2:**
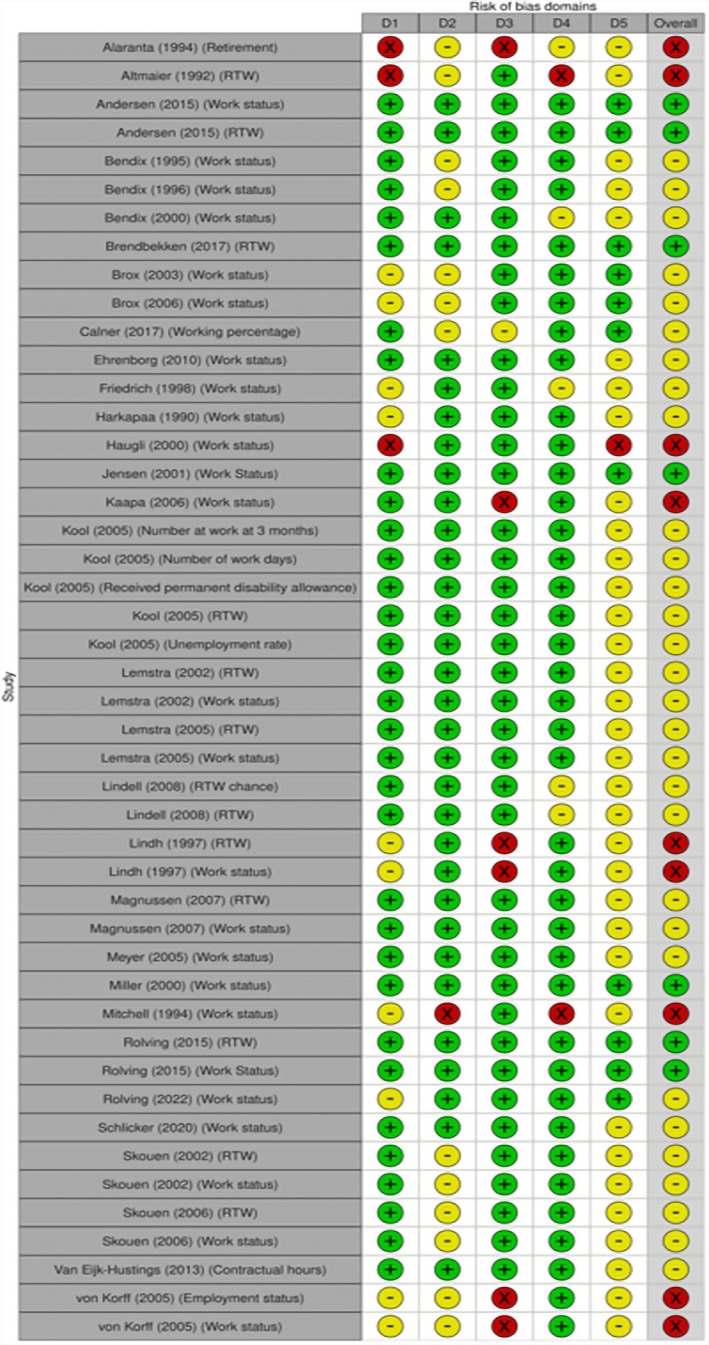
Return to work and work status risk of bias assessment.

Nine of 46 (20%) studies measuring return to work or work status had a high ROB (Figure 2), mostly due to missing data. Most studies raised some concerns (*n* = 30, 65%) largely due to bias in the selection of the reported result. The remaining 7 (15%) outcomes had a low ROB.

**FIGURE 3:**
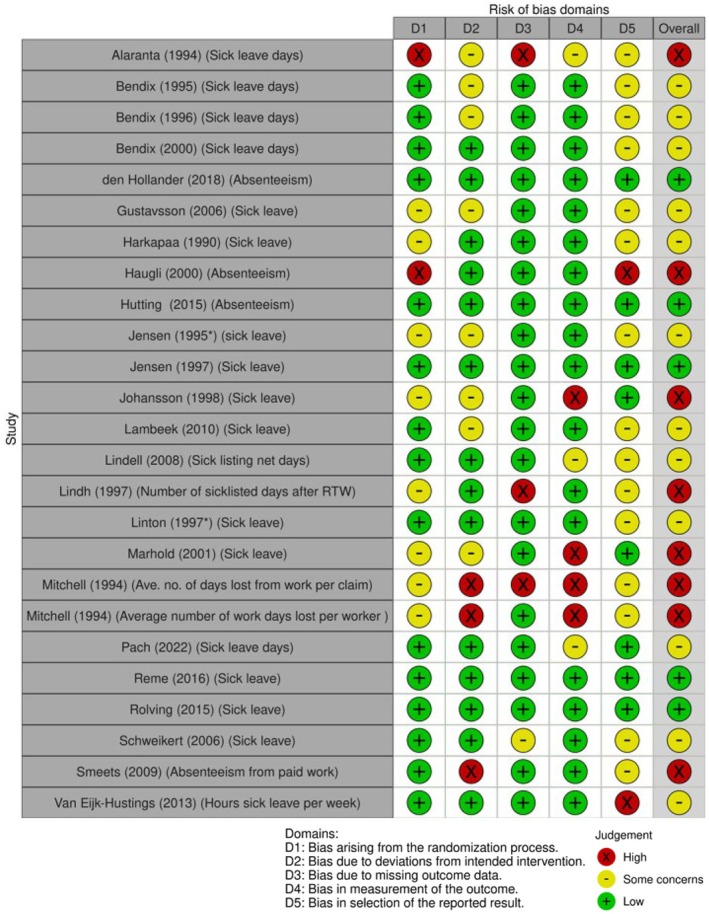
Sick leave risk of bias assessment.

Eight of the 25 (32%) studies measuring sick leave outcomes had a high ROB (Figure 3), largely attributable to bias in the measurement of the outcomes. Twelve outcomes (48%) had some concerns, mostly due to selection bias. The remaining five outcomes (20%) had a low ROB.

**FIGURE 4:**
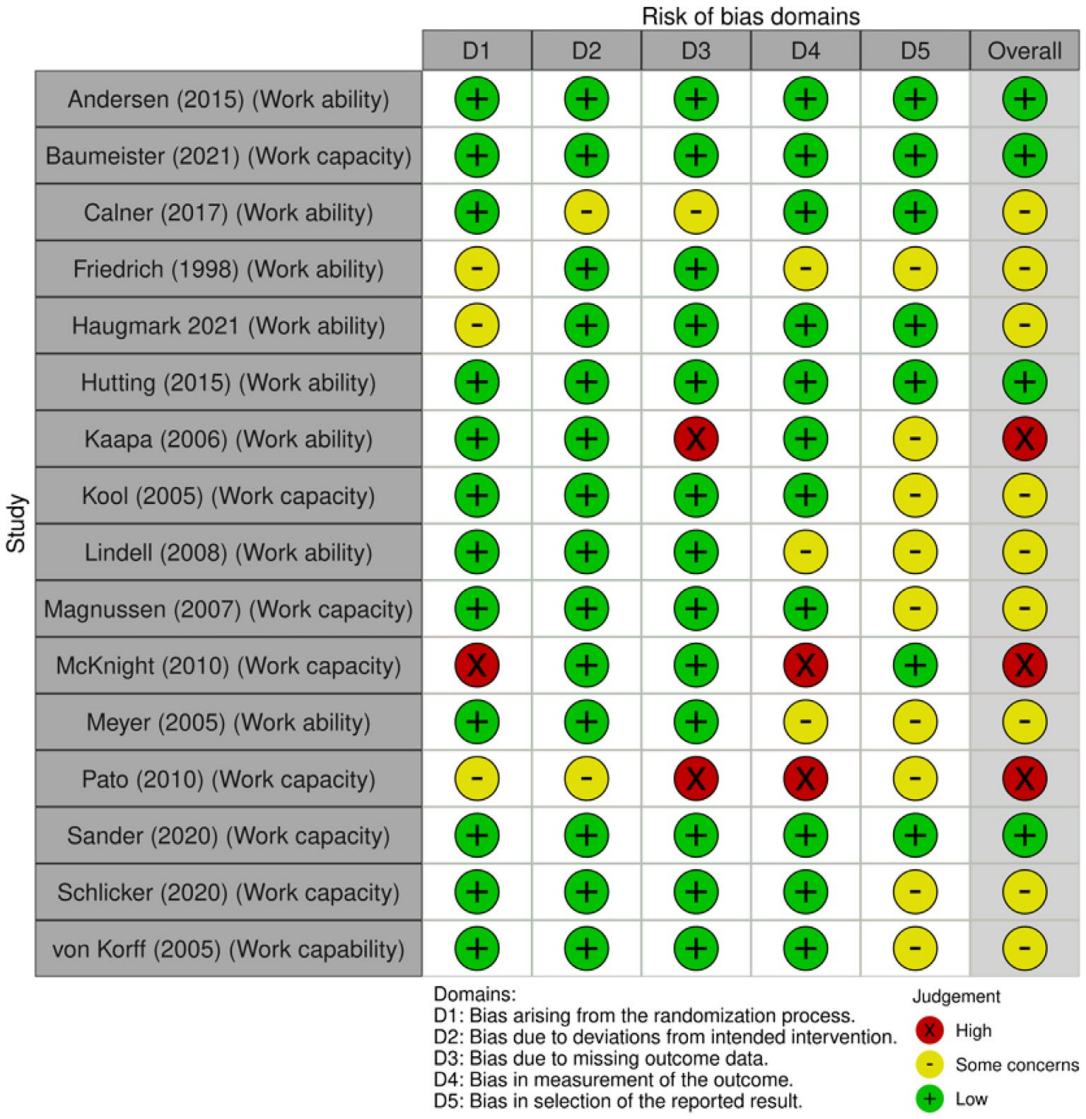
Work ability/capacity risk of bias assessment.

Three of the 16 (19%) studies measuring work ability/work capacity outcomes had a high ROB (Figure 4) mostly owing to missing data. Nine outcomes (56%) had some concerns mostly due to selection bias. The four remaining outcomes (25%) had a low ROB.

**FIGURE 5:**
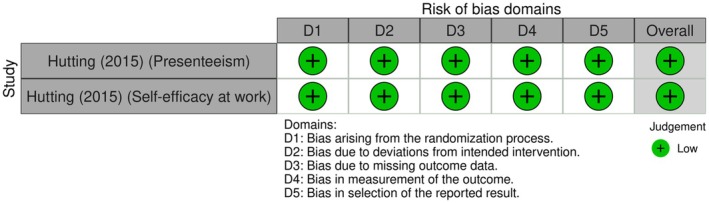
Presenteeism and work‐related self‐efficacy risk of bias assessment.

**FIGURE 6:**
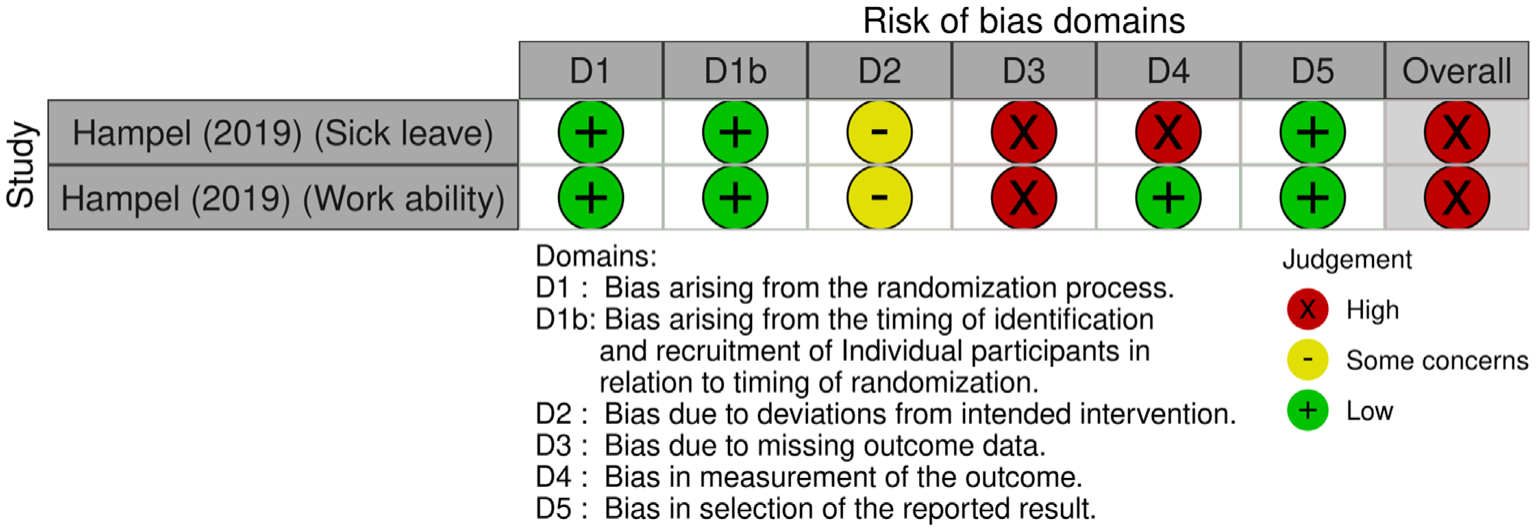
Cluster sick leave and work ability risk of bias assessment.

The one study measuring presenteeism and self‐efficacy as work outcomes (Figure 5) had a low ROB on all domains. One of the RCTs was a cluster trial measuring sick leave and work ability (Figure 6). Both had a high ROB due to missing outcome data.

### Effect of interventions containing psychological components on work outcomes

#### Work status

**FIGURE 7:**
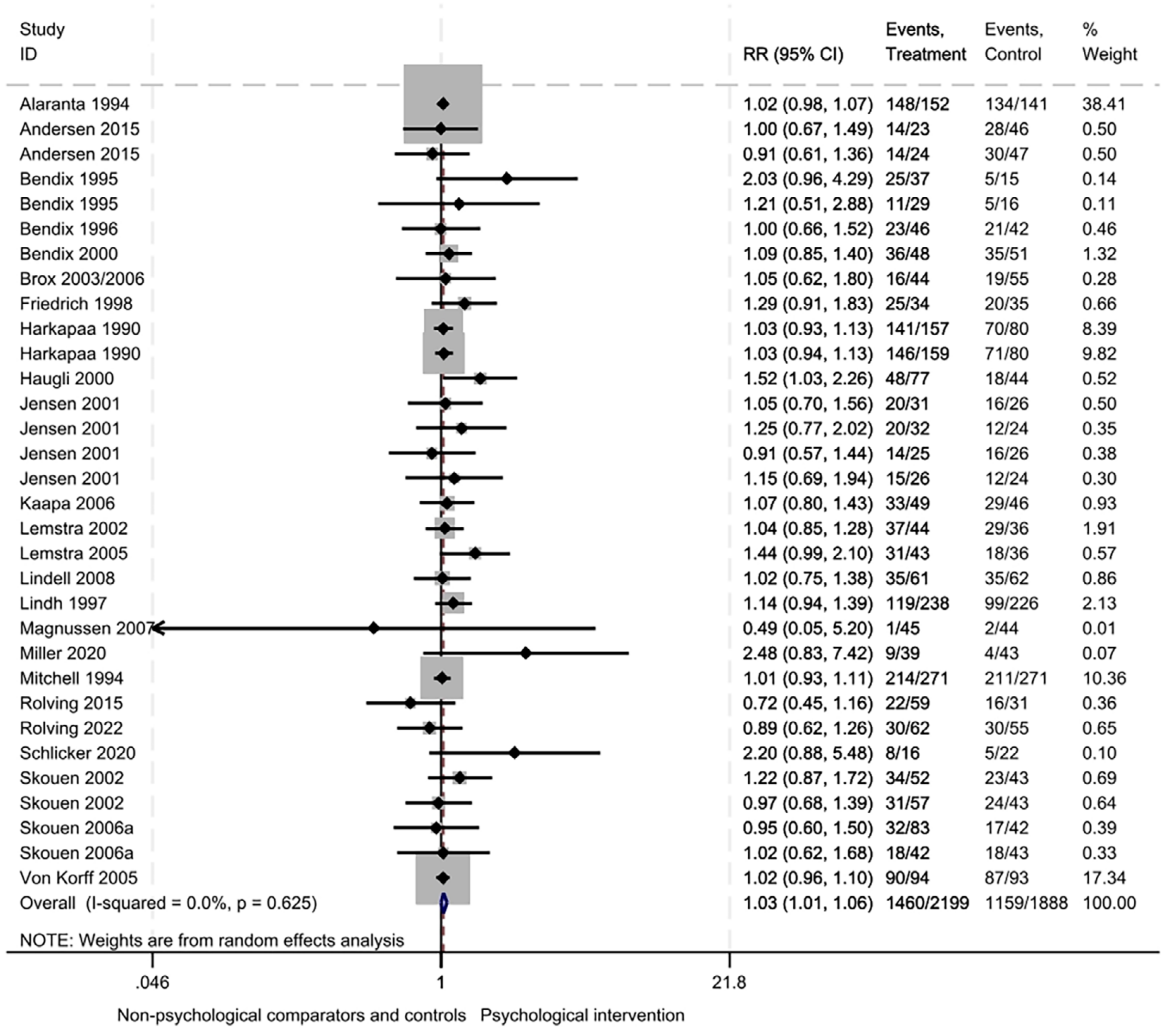
Work status at the longest follow‐up.

Evidence from 24 RCTs (32 comparisons, 4087 participants) showed at longest follow‐up (mean 40 ± 39 months) a 3% increase in the proportion of people at work after interventions including psychological components compared with comparators/controls (RR 1.03, 95% CI: 1.01–1.06; *I* ^2^ = 0%; Figure 7).

There was evidence of publication bias, with funnel plot asymmetry and significant Egger's test (*p* = .04; see Supplementary file 8, panel A). GRADE assessment of certainty was judged to be very low (Supplementary file 9). Evidence was downgraded for ROB: poor reporting of population characteristics, variation in interventions, outcome definitions and measurement methods, and evidence of publication bias.

Across different follow‐up time points (Supplementary file 10) there was no difference in work status at 0–3 months (RR 1.05, 95% CI: .93–1.19; *I* ^2^ = 22%). Between 3–6 month follow‐up there was a 29% increase in the proportion of people at work (RR 1.29, 95% CI: 1.02–1.64; *I* ^2^ = 78%). At 6–12 months there was no difference (RR 1.07, 95% CI: .99–1.42; *I* ^2^ = 53%), nor beyond 12 months (RR 1.03, 95% CI: 1.00–1.07; *I* ^2^ = 0%).

Meta‐regression showed no intervention functions or theoretical domains were more effective than others at improving work status. There was evidence that the inclusion of BCT 2.4: Self‐monitoring the outcomes of behaviour led to increased treatment effects (RR 2.24, 95% CI: 1.08–4.65, *p* = .03); however, this must be interpreted with caution as only two studies included this BCT, and there is a risk of Type 1 error (Supplementary file 11). There was no evidence of differences in treatment effects according to ROB (Supplementary file 12), the type of professional involved in the delivery of the intervention (Supplementary file 13), delivery mode (Supplementary file 14), or interventions containing clear work components (Supplementary file 15).

#### Return to work

**FIGURE 8:**
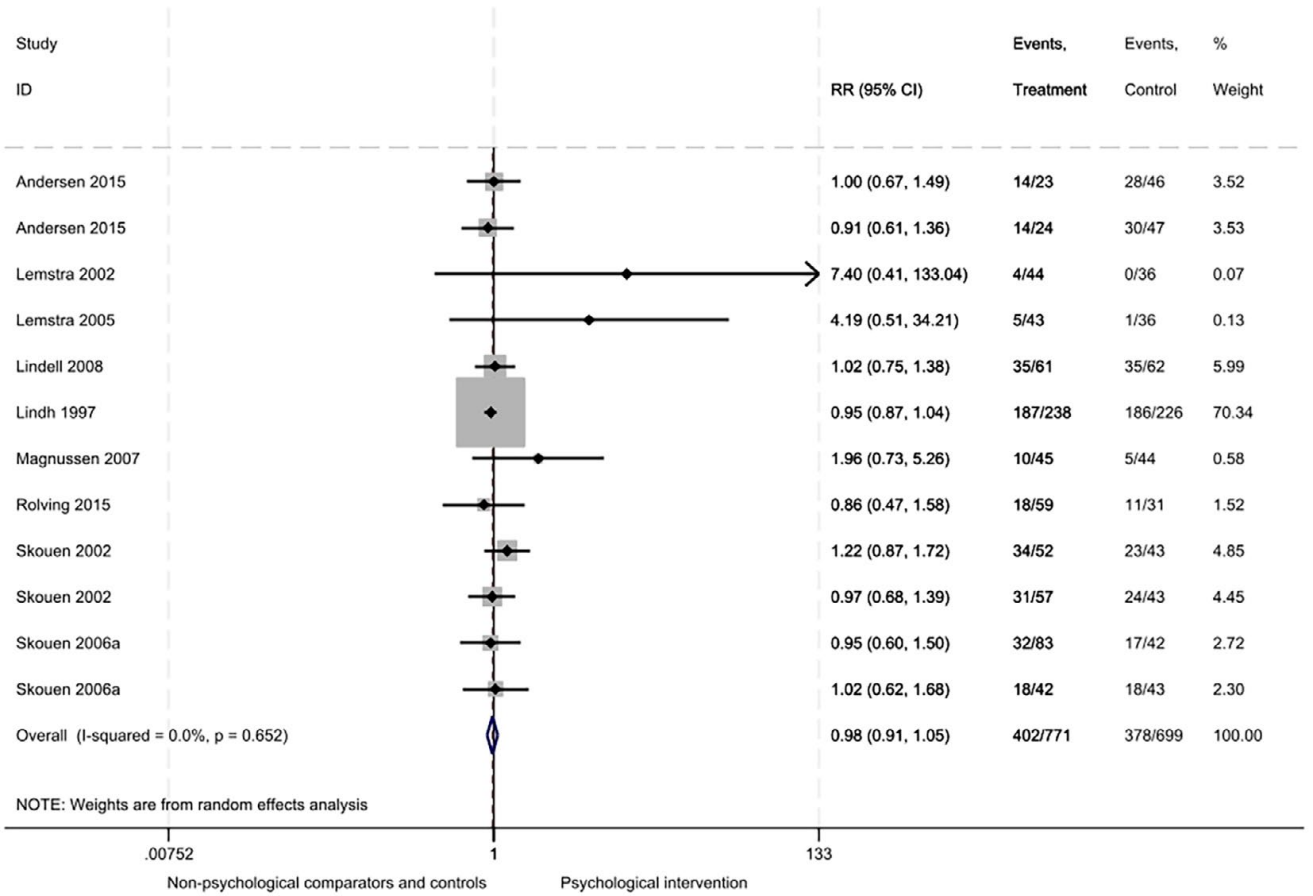
Return to work at the longest follow‐up.

Evidence from 9 RCTs (12 comparisons, 1470 participants) showed that at the longest follow‐up (mean 24 ± 21 months), there was no difference in return to work comparing interventions including psychological components and comparators/controls (RR .98, 95% CI: .91–1.05, *I* ^2^ = 0%; Figure 8). No differences in intervention effects on return to work at any other time point were shown (Supplementary file 16).

There was evidence of publication bias, with funnel plot asymmetry and a significant Egger's test (*p* = .02; Supplementary file 8, panel B). GRADE assessment of certainty was judged to be very low (Supplementary file 9). Evidence was downgraded due to high ROB, poor reporting of population characteristics, variation in interventions, outcome definitions and measurement methods, evidence of publication bias, and 95% CI which overlaps the null effect.

Meta‐regression found no particular intervention functions, theoretical domains, or BCTs were more effective at improving return to work (Supplementary file 11). Similarly, no differences were seen relating to the type of professionals involved in delivering the interventions (*p* > .05; Supplementary file 13), ROB (Supplementary file 17), intervention delivery mode (Supplementary file 18), or clear work components (Supplementary file 19).

#### Sick leave

**FIGURE 9:**
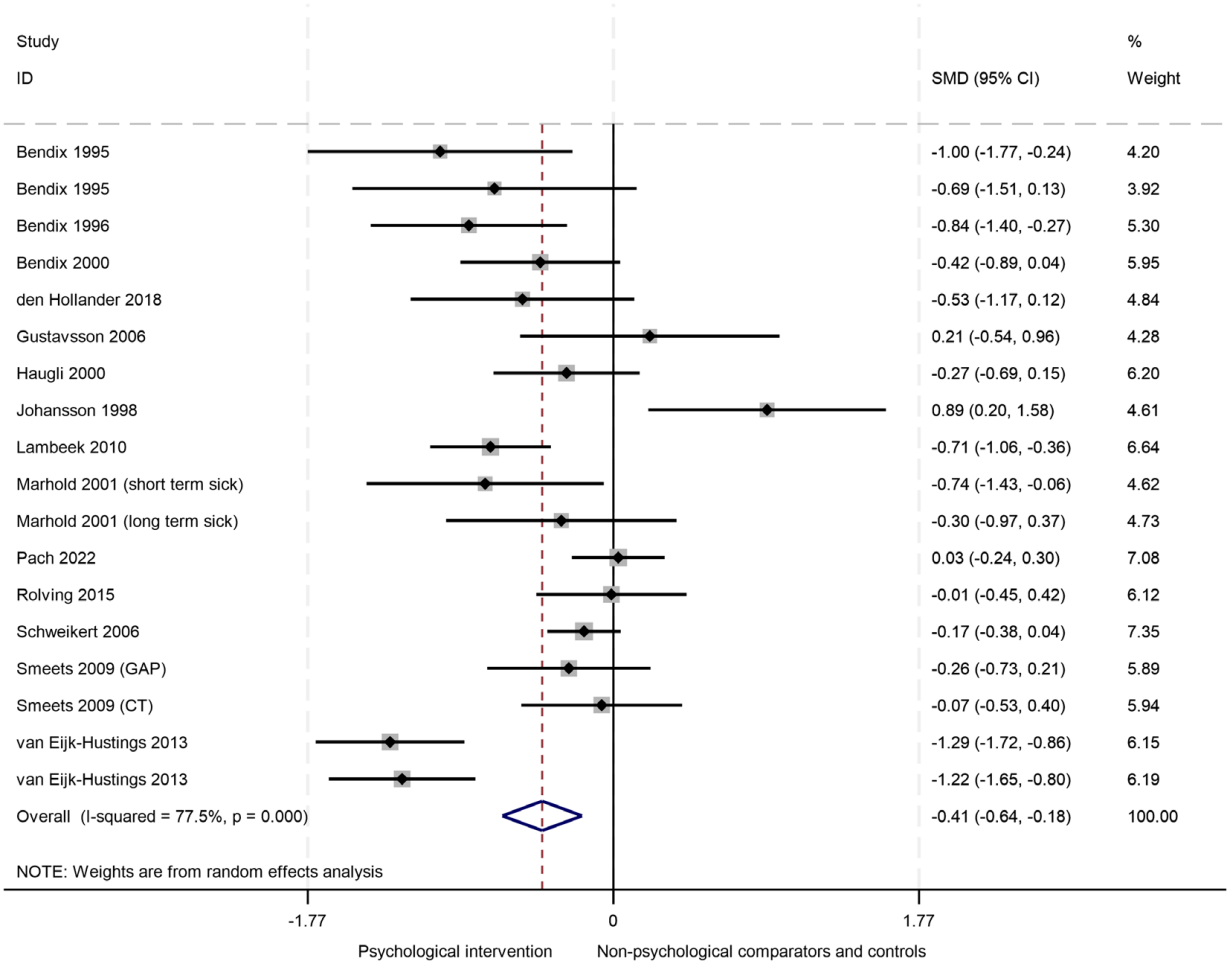
Sick leave at the longest follow‐up.

Evidence from 14 RCTs (18 comparisons, 1607 participants) showed a small effect in favour of interventions containing psychological components at the longest follow‐up time point (mean 12.5 ± 8 months; SMD −.41, 95% CI: −.64 to −.18, Cohen's effect size .4 = small; Figure 9). There was evidence of substantial heterogeneity: *I* ^2^ = 78%, but no evidence of publication bias (Supplementary file 8, panel C; Egger's test *p* = .4). GRADE assessment of certainty was judged to be very low (Supplementary file 9). Evidence was downgraded due to ROB, poor reporting of population characteristics, variation in interventions, outcome definitions and measurement methods, and substantial heterogeneity.

At 0–3 months follow‐up, there was no evidence that psychological interventions reduced sick leave (SMD .38, 95% CI: −.16 to .93). At >3–6 months and >6–12 months follow‐up, there was evidence of a small effect: SMD −.36 (95% CI: −.58 to −.13), *I* ^2^ = 53%, and SMD −.36 (95% CI: −.57 to −.14), *I* ^2^ = 42, respectively. At >12 months follow‐up, there was evidence of a large effect: SMD −1.11 (95% CI: −1.35 to −.87), *I* ^2^ = 0% (Supplementary file 20).

Meta‐regression analyses showed no particular intervention functions or theoretical domains were more effective (Supplementary file 11). There was some evidence that 2 BCTs possibly reduced the effect size: 10.4 Social reward: (RR 1.36, 95% CI: .25–2.48, *p* = .02); and 15.4 Self‐talk: (RR 1.36, 95% CI: .25–2.48, *p* = .02); however, these results should be interpreted with caution due to the low number of studies including these BCTs, one of which we identified as a potential outlier, and high risk for Type 1 error (Johansson et al., 1998).

There was weak evidence that interventions delivered individually (SMD −.30, 95% CI: −.68 to .08) may be slightly less effective at reducing sick leave than group‐based (SMD −.53, 95% CI: −.91 to −.15) and hybrid interventions (SMD −.39, 95% CI: −.72 to −.06; supplementary file 21). Studies with low ROB (SMD −.21, 95% CI: −.70 to .28; supplementary file 22) and high ROB (SMD −.14, 95% CI: −.49 to .21) had little to no effect compared with RCTs judged to have some concerns (SMD −.6, 95% CI: −.93 to .27). There were no differences in intervention effects relating to professionals involved (Supplementary file 13) or inclusion of work‐related components (Supplementary file 23).

#### Sensitivity analysis

Johansson et al. (1998) was identified as an outlying study regarding sick leave, with results strongly in favour of the waitlist control comparator. Further investigation did not provide any explanation for this. We conducted a sensitivity analysis by removing this study from our primary analysis. The effect size increased slightly (SMD −.47, 95% CI: −.69 to −.25), but heterogeneity remained substantial at 75% (Supplementary file 24).

#### Work ability and work capacity

**FIGURE 10:**
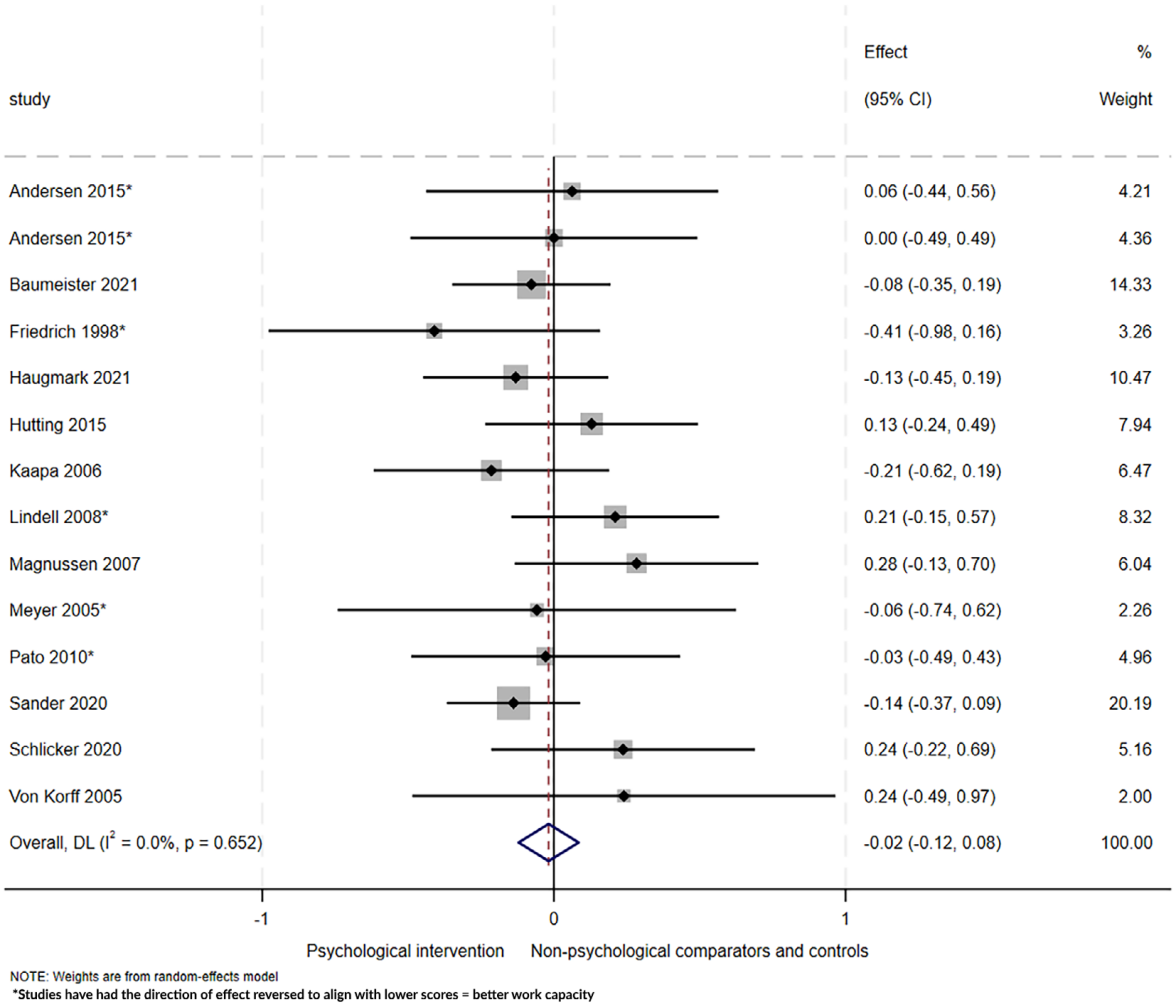
Work ability and capacity at the longest follow‐up.

Evidence from 13 studies (14 comparisons, 1658 participants) showed no evidence that interventions including psychological components improved work ability or capacity at longest follow‐up (mean 12 ± 6 months; SMD −.02, 95% CI: −.12 to .08, *I* ^2^ = 0%; Figure 10), or at any other follow‐up time point (Supplementary file 25).

There was no evidence of publication bias (Supplementary file 8, panel D; Egger's test *p* = .3). GRADE assessment of certainty was judged to be very low (Supplementary file 9). Evidence was downgraded due to ROB, poor reporting of population characteristics, variation in interventions, outcome definitions and measurement methods, and 95% CI which overlapped the null effect.

Meta‐regression analyses found no particular intervention functions, theoretical domains, or BCTs were more effective than others (*p* > .05, Supplementary file 11). No differences in intervention effects were seen for ROB (Supplementary file 26), the type of professionals involved in delivering interventions (Supplementary file 13), intervention delivery mode (Supplementary file 27), or interventions containing a clear work component (Supplementary file 28).

#### Narrative syntheses

Additional work‐related outcomes (self‐efficacy at work, presenteeism, return‐to‐work chance, and work simulator) were identified from three studies and synthesized narratively (Supplementary file 29). All reported results showed no statistical difference between intervention and control groups. Eight studies directly compared different interventions containing psychological components, without non‐psychological control arms (Supplementary file 30). In total, only 11 out of 68 (16%) of results reported showed a statistically significant difference between interventions containing psychological components.

### Additional data following updated search

Updated searches identified five potentially relevant papers. Three were excluded due to ineligibility at full‐text screening stage (Aanesen et al., 2023; Lanhers et al., 2024; Ulrich et al., 2024). Two studies reported work capacity outcomes (Cui et al., 2023; Hansen et al., 2023). A sensitivity analysis showed that the addition of these two studies made no difference to the primary work ability/capacity analysis (Supplementary file 31).

## DISCUSSION

Fifty‐one RCTs were identified in our systematic review. Complex interventions with psychological components were no more effective than non‐psychological comparators for reducing presenteeism or increasing work‐related self‐efficacy. They may reduce sick leave and slightly improve long‐term work participation by addressing physical and psychological needs (Nazarov et al., 2019; Skamagki et al., 2018; Venning et al., 2021), although the evidence was very uncertain. Interventions showed no effect on return to work or work ability/capacity, with evidence again very uncertain. This likely reflects limited study quality and inconsistent, often secondary, work outcomes rather than true ineffectiveness. Heterogeneous outcome measures required grouping similar outcomes for meta‐analysis. The lack of improvement in work ability and capacity may reflect our pragmatic decision to combine these outcomes. Evidence shows interventions can improve work ability (perceived ability to meet job demands) (Oakman et al., 2018), but merging it with work capacity, a more objective performance measure, may have obscured effects. This underscores the need to distinguish these constructs in future research. The recent core outcome set for work participation (Ravinskaya et al., 2023) should be adopted to improve measurement consistency and support data pooling.

Only 39% of studies included a work‐focussed component, typically ergonomic support, which may have been insufficient to address return‐to‐work challenges and contributed to the null findings. Employees require collaborative and flexible return‐to‐work support, but difficult engagement with employers and healthcare providers hampers this (Lundin et al., 2024). Fully incorporating support for work within interventions can help to improve work outcomes (Svanholm et al., 2023), but, as found in this review, condition management is often delivered by healthcare professionals independent of the workplace. Better collaboration between clinical staff and the workplace is needed (Svanholm et al., 2022; Williams et al., 2007) to ensure the translation of work‐oriented treatment into return‐to‐work as part of clinical management.

The heterogeneity of the evidence prevented firm conclusions about which intervention functions, theoretical domains, or BCTs improve work participation. However, the intervention components were largely theoretically congruent and 5 common content areas of alignment were identified. First, knowledge/education, and second, training/skills. This included instructions on rehearsing and performing behaviour, for example, exercise or psychological pain management strategies. Third, social influence and social support, involving, for example, family, friends, or the workplace helping set plans for behaviour change. Fourth, addressing negative emotions and stress, involving addressing negative thoughts, and managing fear, anxiety, and anger. Fifth, strategies to promote beliefs about capabilities, including persuasion, confidence building, and boosting self‐efficacy to engage in activity. The coded BCTs are consistent with those found in chronic pain management and work‐focussed intervention reviews, such as instructions to perform a behaviour, the practice and rehearsal of the behaviour and social support (Elbers et al., 2018; Keogh et al., 2015; Meade et al., 2019; Palmer et al., 2012). Identifying BCTs provides a clearer understanding of intervention content, providing a framework for designing and selecting interventions to support people with chronic pain in work. We highlighted BCTs coded in over half of the interventions, but less commonly reported or aligned BCTs that may improve work outcomes were present, including goal setting that could help provide purpose, plan activity, prioritize tasks and improve time management. This is worthy of inclusion in future interventions.

Poor reporting of work characteristics limited interpretation of the findings. Future research should systematically report baseline data on working hours, sick leave, employment status and job type, with precise and consistent definitions that are assessed over time, as they may influence intervention outcomes (Nielsen et al., 2022). For example, inconsistent reporting of sick leave and type of work precluded our ability to draw conclusions about who may have benefitted most from treatment. Evidence suggests those on long‐term sick leave who have the poorest return‐to‐work prognosis may benefit most from interdisciplinary interventions (Constan et al., 2025), but we were unable to draw firm conclusions about this. Furthermore, much of the included literature was outdated. Future research should reflect post‐pandemic changes like AI's impact on job roles, increased gig work, self‐employment, and working from home. Notably, work‐focussed interventions were more likely to involve social workers or occupational therapists, suggesting that subject matter expertise may also influence intervention design.

This review has several strengths. The protocol was prospectively registered, followed Cochrane guidelines for conduct and adhered to PRISMA reporting standards. We also searched grey literature to enhance comprehensiveness. However, heterogeneity, high ROB and low certainty of evidence, often due to poor measurement and reporting of work outcomes, affected confidence in our findings. We did not use the updated Behaviour Change Ontology (Marques et al., 2024), as it was unavailable when we began coding the data, although a mapping exercise showed 79% alignment with our BCT coding. Inter‐rater reliability was not calculated, as intervention functions and TDF domains lack coding rules as are available for BCT coding, but we developed guidelines among experienced coders, focusing on consensus and distinguishing between confident and uncertain coding. Retrospective coding was used to identify intervention components, highlighting the need for research to explicitly incorporate BCTs to clarify active ingredients and standardize intervention development.

## CONCLUSION

In conclusion, our evidence synthesis showed that complex, multidisciplinary, psychological interventions can improve sick leave and work status; however, further investigations using well‐designed, work‐focussed studies with longer term follow‐up are needed. Five common components of the interventions were identified that provide direction to work‐focussed future research and practice. Our findings align with initiatives to improve work outcomes for employees with pain and health problems (Department for Work and Pensions, 2024), notably the Pain at Work Toolkit (Blake et al., 2022) to promote the self‐management of pain at work.

## AUTHOR CONTRIBUTIONS

**Joanna McParland:** Conceptualization; investigation; funding acquisition; writing – original draft; methodology; validation; visualization; writing – review and editing; formal analysis; project administration; supervision. **Lorna Booth:** Investigation; writing – original draft; validation; formal analysis; project administration; methodology; writing – review and editing. **Grace Dibben:** Formal analysis; methodology; visualization; writing – original draft; writing – review and editing; conceptualization; investigation; project administration. **Ukachukwu Abaraogu:** Conceptualization; funding acquisition; methodology; supervision; writing – original draft; project administration; writing – review and editing. **Elaine Wainwright:** Conceptualization; funding acquisition; writing – original draft; methodology; writing – review and editing; validation; project administration. **Evangelia Demou:** Conceptualization; funding acquisition; writing – original draft; methodology; validation; project administration; writing – review and editing. **Lynn Williams:** Conceptualization; funding acquisition; writing – original draft; validation; methodology; formal analysis; project administration; writing – review and editing. **Paul Flowers:** Conceptualization; funding acquisition; writing – original draft; validation; methodology; formal analysis; project administration; writing – review and editing. **Lisa Kidd:** Conceptualization; funding acquisition; writing – original draft; methodology; writing – review and editing; project administration. **Jo Daniels:** Methodology; project administration; writing – original draft; writing – review and editing. **Hussein Patwa:** Funding acquisition; methodology; project administration. **Paulina Wegrzynek:** Writing – original draft; validation; – review and editing. **Sarah Audsley:** Writing – original draft; writing – review and editing; validation. **Ronald O'Kane:** Writing – original draft; validation. **Amelia Parchment:** Writing – original draft; writing – review and editing; validation. **Hannah Ranaldi:** Validation. **Karen Walker‐Bone:** Conceptualization; funding acquisition; writing – original draft; writing – review and editing; methodology; project administration; supervision.

## FUNDING INFORMATION

This project was funded by the NIHR (NIHR 203430/Policy Research Programme). The views expressed are those of the author(s) and not necessarily those of the NIHR or the Department of Health and Social Care. ED wishes to acknowledge funding from the Medical Research Council, United Kingdom (MC_UU_00022/2) and the Chief Scientist Office, Scottish Government Health and Social Care Directorate (SPHSU17).

## CONFLICT OF INTEREST STATEMENT

Dr. Jo Daniels works for the Department of Health and Social Care. This project was funded by the NIHR (NIHR 203430/Policy Research Programme). The views expressed are those of the author(s) and not necessarily those of the NIHR or the Department of Health and Social Care.

## ETHICS STATEMENT

Ethical approval was not required for this review. The review protocol was registered on the PROSPERO database (registration number: CRD42022375328).

## Supporting information


**Supplementary file 1:** PRISMA checklist.
**Supplementary File 2:** Medline (EBSCO) search strategy.
**Supplementary file 4:** linked RCT papers.
**Supplementary file 5:** RCT intervention description and congruence analysis.
**Supplementary file 6:** Distribution of intervention functions, TDF domains and Behavioural Change Techniques.
**Supplementary file 7:** Description and mapping of the five common intervention components.
**Supplementary file 8:** Panel graph displaying publication bias funnel plots for (A) work status; (B) return to work; (C) sick leave; and (D) work capacity.
**Supplementary file 9:** GRADE summary of findings for all work outcomes.
**Supplementary file10:** Effectiveness of interventions containing psychological components in relation to work status at different follow‐up time points.
**Supplementary file 11:** Meta‐analysis of intervention coding components for all outcomes.
**Supplementary file 12:** Risk of bias in studies included in work status analysis.
**Supplementary file 13:** meta‐regression results for intervention delivery (all outcomes).
**Supplementary file 14:** Intervention delivery mode in relation to work status.
**Supplementary file 15:** Comparison of interventions containing a work component versus no work component in relation to work status.
**Supplementary file 16:** Different follow‐up time periods in relation to return to work.
**Supplementary file 17:** Risk of bias in relation to return to work.
**Supplementary file 18:** Intervention delivery mode in relation to return to work.
**Supplementary file 19:** Comparison of interventions containing a work component versus no work component in relation to return to work.
**Supplementary file 20:** Comparisons of interventions and comparator/controls in relation to sick leave at different follow‐up time points.
**Supplementary file 21:** Intervention delivery mode in relation to sick leave.
**Supplementary file 22:** Risk of bias in relation to sick leave.
**Supplementary file 23:** Comparison of interventions containing a work component versus those that don't in relation to sick leave.
**Supplementary file 24:** Sick leave sensitivity analysis for Johansson et al. (1998) paper.
**Supplementary file 25:** Interventions and comparator/controls in relation to work ability and work capacity at different follow‐up time points.
**Supplementary file 26:** Risk of bias in relation to work ability/capacity.
**Supplementary file 27:** Intervention delivery mode for work ability/capacity.
**Supplementary file 28:** Comparison of interventions containing a work component versus those that do not in relation to work ability and capacity.
**Supplementary file 29:** RCT Narrative synthesis of work outcomes.
**Supplementary file 30:** Narrative synthesis: RCT psychological interventions versus psychological interventions.
**Supplementary file 31:** Sensitivity analysis including additional papers.

Supplementary File 3:

## Data Availability

This is a systematic review and no primary data were collected. The review was registered with PROSPERO (ID: CRD42022375328).
