# Supplementary material for: Interventions to enhance work participation in people with chronic pain: A systematic review and meta‐analysis including analysis of complex psychological intervention components
Source: Br J Health Psychol. 2026 May 8;31:e70077. doi: 10.1111/bjhp.70077 (PMC13155186; doi:10.1111/bjhp.70077)
Supplement: Supplementary file 1 — PRISMA checklist. Supplementary File 2: Medline (EBSCO) search strategy. Supplementary file 4: linked RCT papers. Supplementary file 5: RCT intervention description and congruence analysis. Supplementary file 6: Distribution of intervention functions, TDF domains and Behavioural Change Techniques. Supplementary file 7: Description and mapping of the five common intervention components. Supplementary file 8: Panel graph displaying publication bias funnel plots for (A) work status; (B) return to work; (C) sick leave; and (D) work capacity. Supplementary file 9: GRADE summary of findings for all work outcomes. Supplementary file10: Effectiveness of interventions containing psychological components in relation to work status at different follow‐up time points. Supplementary file 11: Meta‐analysis of intervention coding components for all outcomes. Supplementary file 12: Risk of bias in studies included in work status analysis. Supplementary file 13: meta‐regression results for intervention delivery (all outcomes). Supplementary file 14: Intervention delivery mode in relation to work status. Supplementary file 15: Comparison of interventions containing a work component versus no work component in relation to work status. Supplementary file 16: Different follow‐up time periods in relation to return to work. Supplementary file 17: Risk of bias in relation to return to work. Supplementary file 18: Intervention delivery mode in relation to return to work. Supplementary file 19: Comparison of interventions containing a work component versus no work component in relation to return to work. Supplementary file 20: Comparisons of interventions and comparator/controls in relation to sick leave at different follow‐up time points. Supplementary file 21: Intervention delivery mode in relation to sick leave. Supplementary file 22: Risk of bias in relation to sick leave. Supplementary file 23: Comparison of interventions containing a work component versus those th [file BJHP-31-0-s002.docx]

Supplementary file 1: PRISMA checklist

| **Section and Topic** | **Item #** | **Checklist item** | **Location where item is reported** |
| --- | --- | --- | --- |
| **TITLE** | | |  |
| Title | 1 | Identify the report as a systematic review. | Title |
| **ABSTRACT** | | |  |
| Abstract | 2 | See the PRISMA 2020 for Abstracts checklist. | Abstract |
| **INTRODUCTION** | | |  |
| Rationale | 3 | Describe the rationale for the review in the context of existing knowledge. | Introduction |
| Objectives | 4 | Provide an explicit statement of the objective(s) or question(s) the review addresses. | Introduction |
| **METHODS** | | |  |
| Eligibility criteria | 5 | Specify the inclusion and exclusion criteria for the review and how studies were grouped for the syntheses. | Methods – Information sources and inclusion criteria |
| Information sources | 6 | Specify all databases, registers, websites, organisations, reference lists and other sources searched or consulted to identify studies. Specify the date when each source was last searched or consulted. | Methods – Information sources and inclusion criteria |
| Search strategy | 7 | Present the full search strategies for all databases, registers and websites, including any filters and limits used. | Supplementary file 2 |
| Selection process | 8 | Specify the methods used to decide whether a study met the inclusion criteria of the review, including how many reviewers screened each record and each report retrieved, whether they worked independently, and if applicable, details of automation tools used in the process. | Methods -study selection |
| Data collection process | 9 | Specify the methods used to collect data from reports, including how many reviewers collected data from each report, whether they worked independently, any processes for obtaining or confirming data from study investigators, and if applicable, details of automation tools used in the process. | Methods – study selection, risk of bias, GRADE certainty of evidence |
| Data items | 10a | List and define all outcomes for which data were sought. Specify whether all results that were compatible with each outcome domain in each study were sought (e.g. for all measures, time points, analyses), and if not, the methods used to decide which results to collect. | Methods -information sources and inclusion criteria |
|  | 10b | List and define all other variables for which data were sought (e.g. participant and intervention characteristics, funding sources). Describe any assumptions made about any missing or unclear information. | Methods – Data extraction |
| Study risk of bias assessment | 11 | Specify the methods used to assess risk of bias in the included studies, including details of the tool(s) used, how many reviewers assessed each study and whether they worked independently, and if applicable, details of automation tools used in the process. | Methods -Risk of bias assessment |
| Effect measures | 12 | Specify for each outcome the effect measure(s) (e.g. risk ratio, mean difference) used in the synthesis or presentation of results. | Methods -Data synthesis and statistical analysis |
| Synthesis methods | 13a | Describe the processes used to decide which studies were eligible for each synthesis (e.g. tabulating the study intervention characteristics and comparing against the planned groups for each synthesis (item #5)). | Methods -Data synthesis and statistical analysis |
|  | 13b | Describe any methods required to prepare the data for presentation or synthesis, such as handling of missing summary statistics, or data conversions. | Methods -Data synthesis and statistical analysis |
|  | 13c | Describe any methods used to tabulate or visually display results of individual studies and syntheses. | Methods -Data synthesis and statistical analysis |
|  | 13d | Describe any methods used to synthesize results and provide a rationale for the choice(s). If meta-analysis was performed, describe the model(s), method(s) to identify the presence and extent of statistical heterogeneity, and software package(s) used. | Methods -Data synthesis and statistical analysis |
|  | 13e | Describe any methods used to explore possible causes of heterogeneity among study results (e.g. subgroup analysis, meta-regression). | Methods -Data synthesis and statistical analysis |
|  | 13f | Describe any sensitivity analyses conducted to assess robustness of the synthesized results. | Methods -Data synthesis and statistical analysis |
| Reporting bias assessment | 14 | Describe any methods used to assess risk of bias due to missing results in a synthesis (arising from reporting biases). | Methods-Risk of bias assessment |
| Certainty assessment | 15 | Describe any methods used to assess certainty (or confidence) in the body of evidence for an outcome. | Methods -GRADE certainty of evidence |
| **RESULTS** | | |  |
| Study selection | 16a | Describe the results of the search and selection process, from the number of records identified in the search to the number of studies included in the review, ideally using a flow diagram. | Figure 1 |
|  | 16b | Cite studies that might appear to meet the inclusion criteria, but which were excluded, and explain why they were excluded. | Supplementary file 3 |
| Study characteristics | 17 | Cite each included study and present its characteristics. | Tables 1 and 2, Results -Study. participant and intervention characteristics |
| Risk of bias in studies | 18 | Present assessments of risk of bias for each included study. | Results -Risk of bias; Figures 2-6 |
| Results of individual studies | 19 | For all outcomes, present, for each study: (a) summary statistics for each group (where appropriate) and (b) an effect estimate and its precision (e.g. confidence/credible interval), ideally using structured tables or plots. | Figures 7-10 |
| Results of syntheses | 20a | For each synthesis, briefly summarise the characteristics and risk of bias among contributing studies. | Results -Risk of bias |
|  | 20b | Present results of all statistical syntheses conducted. If meta-analysis was done, present for each the summary estimate and its precision (e.g. confidence/credible interval) and measures of statistical heterogeneity. If comparing groups, describe the direction of the effect. | Results – Effects of interventions on work outcomes |
|  | 20c | Present results of all investigations of possible causes of heterogeneity among study results. | Results – Effects of interventions on work outcomes; Supplementary files 8-28 |
|  | 20d | Present results of all sensitivity analyses conducted to assess the robustness of the synthesized results. | Results – Sensitivity analysis |
| Reporting biases | 21 | Present assessments of risk of bias due to missing results (arising from reporting biases) for each synthesis assessed. | Results -Risk of bias; Figures 2-6, Supplementary file 8 |
| Certainty of evidence | 22 | Present assessments of certainty (or confidence) in the body of evidence for each outcome assessed. | Results -Effects of interventions on work outcomes; Supplementary file 9 |
| **DISCUSSION** | | |  |
| Discussion | 23a | Provide a general interpretation of the results in the context of other evidence. | Discussion |
|  | 23b | Discuss any limitations of the evidence included in the review. | Discussion |
|  | 23c | Discuss any limitations of the review processes used. | Discussion |
|  | 23d | Discuss implications of the results for practice, policy, and future research. | Discussion |
| **OTHER INFORMATION** | | |  |
| Registration and protocol | 24a | Provide registration information for the review, including register name and registration number, or state that the review was not registered. | Methods |
|  | 24b | Indicate where the review protocol can be accessed, or state that a protocol was not prepared. | Methods |
|  | 24c | Describe and explain any amendments to information provided at registration or in the protocol. | N/A |
| Support | 25 | Describe sources of financial or non-financial support for the review, and the role of the funders or sponsors in the review. | Funding information |
| Competing interests | 26 | Declare any competing interests of review authors. | COI statement |
| Availability of data, code and other materials | 27 | Report which of the following are publicly available and where they can be found: template data collection forms; data extracted from included studies; data used for all analyses; analytic code; any other materials used in the review. | N/A |

*From:*  Page MJ, McKenzie JE, Bossuyt PM, Boutron I, Hoffmann TC, Mulrow CD, et al. The PRISMA 2020 statement: an updated guideline for reporting systematic reviews. BMJ 2021;372:n71. doi: 10.1136/bmj.n71. This work is licensed under CC BY 4.0. To view a copy of this license, visit <https://creativecommons.org/licenses/by/4.0/>

**Supplementary File 2: Medline (EBSCO) search strategy**

| **#** | **Query** |
| --- | --- |
| S161 | S43 AND S112 AND S160 |
| S160 | S113 OR S114 OR S115 OR S116 OR S117 OR S118 OR S119 OR S120 OR S121 OR S122 OR S123 OR S124 OR S125 OR S126 OR S127 OR S128 OR S129 OR S130 OR S131 OR S132 OR S133 OR S134 OR S135 OR S136 OR S137 OR S138 OR S139 OR S140 OR S141 OR S142 OR S143 OR S144 OR S145 OR S146 OR S147 OR S148 OR S149 OR S150 OR S151 OR S152 OR S153 OR S154 OR S155 OR S156 OR S157 OR S158 OR S159 |
| S159 | "reduce* hours" |
| S158 | "need for recovery" |
| S157 | "work* ability" OR workability OR "ability to work" |
| S156 | "job retention" |
| S155 | (work* OR employee) N1 attendance |
| S154 | (work* OR job) N1 maintenance |
| S153 | "work* limitation*" |
| S152 | (MH "Unemployment") |
| S151 | unemployment |
| S150 | worklessness |
| S149 | (work* OR employee OR job) N1 accommodation |
| S148 | (MH "Presenteeism") |
| S147 | presenteeism |
| S146 | "sustain* employ*" |
| S145 | "work* inclusion" |
| S144 | (work* OR employee) N1 readiness |
| S143 | "work resumption" |
| S142 | (MH "Employment Status") |
| S141 | (work* OR employ*) N1 status |
| S140 | "work* capacity" |
| S139 | (work* OR employee) N1 retention |
| S138 | (MH "Job Performance") |
| S137 | (work* OR employee OR job) N1 performance |
| S136 | (MH "Job Re-Entry") |
| S135 | "job re-entry" OR "job reentry" |
| S134 | "work* sustainability" |
| S133 | (work* OR employee) N1 participation |
| S132 | "stay at work" OR stay-at-work |
| S131 | (work* OR employee OR job) N1 productivity |
| S130 | (MH "Work Engagement") |
| S129 | (work* OR employee) N1 engagement |
| S128 | "work* reintegration" |
| S127 | "work adjustment training" |
| S126 | reemployment OR re-employment |
| S125 | (work* OR employee) N1 efficiency |
| S124 | (work* OR employee) N1 effectiveness |
| S123 | (work* OR job) N1 loss |
| S122 | "work-related self-efficacy" OR "return to work self-efficacy" OR "return-to-work self-efficacy" |
| S121 | (work* OR employee) N1 absence |
| S120 | (MH "Absenteeism") |
| S119 | absenteeism |
| S118 | "employee leave benefit*" |
| S117 | "sick listed" OR sick-listed |
| S116 | (MH "Sick Leave") |
| S115 | "sick leave" OR sick-leave OR sickleave |
| S114 | "sickness absence" |
| S113 | "return* to work" OR return-to-work OR "back to work" OR back-to-work |
| S112 | S44 OR S45 OR S46 OR S47 OR S48 OR S49 OR S50 OR S51 OR S52 OR S53 OR S54 OR S55 OR S56 OR S57 OR S58 OR S59 OR S60 OR S61 OR S62 OR S63 OR S64 OR S65 OR S66 OR S67 OR S68 OR S69 OR S70 OR S71 OR S72 OR S73 OR S74 OR S75 OR S76 OR S77 OR S78 OR S79 OR S80 OR S81 OR S82 OR S83 OR S84 OR S85 OR S86 OR S87 OR S88 OR S89 OR S90 OR S91 OR S92 OR S93 OR S94 OR S95 OR S96 OR S97 OR S98 OR S99 OR S100 OR S101 OR S102 OR S103 OR S104 OR S105 OR S106 OR S107 OR S108 OR S109 OR S110 OR S111 |
| S111 | (MH "Rehabilitation, Vocational") |
| S110 | (MH "Rehabilitation") |
| S109 | rehabilitation or "vocational rehabilitation" or "occupational rehabilitation" or "cognitive behavio* rehabilitation" or "work* rehabilitation" |
| S108 | "rational emotive therap*" OR "rational-emotive therap*" |
| S107 | multifaceted N3 (intervention* OR treatment*) |
| S106 | interdisciplinary N3 (intervention* OR treatment*) |
| S105 | multidisciplinary N3 (intervention* OR treatment*) |
| S104 | Self-help OR "Self help" |
| S103 | (MH "Self Care") |
| S102 | (MH "Self-Management") |
| S101 | "self manage*" OR self-manage* OR self-care OR "self care" |
| S100 | "behavio* change technique*" |
| S99 | "behavio* change" |
| S98 | "cognitive behavio* stress management" |
| S97 | "systematic desensiti#ation" |
| S96 | "behavio* experiment*" |
| S95 | (MH "Goal-Setting") |
| S94 | "goal setting" OR goal-setting |
| S93 | "graded exposure" |
| S92 | "graded activity" |
| S91 | (MH "Reinforcement (Psychology)") |
| S90 | reinforcement |
| S89 | "activity scheduling" |
| S88 | pacing |
| S87 | (MH "Eye Movement Desensitization and Reprogramming") |
| S86 | "eye movement desensiti#ation" |
| S85 | "functional restoration program*" |
| S84 | "in vivo exposure" |
| S83 | "behavio#ral activation" |
| S82 | (MH "Assertiveness Training") |
| S81 | "assertiveness training" |
| S80 | (MH "Problem Solving") |
| S79 | "problem solving" OR problem-solving |
| S78 | (MH "Cognitive Restructuring") |
| S77 | "cognitive restructuring" |
| S76 | (MH "Health Education") |
| S75 | "health education" |
| S74 | "health coaching" |
| S73 | visualization OR visualisation |
| S72 | (MH "Guided Imagery") |
| S71 | imagery |
| S70 | (vocation* OR work*) N1 intervention* |
| S69 | (MH "Psychoeducation") |
| S68 | psychoeducation OR psycho-education |
| S67 | (MH "Relaxation") |
| S66 | relaxation OR "autogenic training" |
| S65 | (MH "Biofeedback") |
| S64 | biofeedback |
| S63 | (MH "Counseling") |
| S62 | counseling OR counselling |
| S61 | psychological N1 (therap* OR intervention* OR treatment*) |
| S60 | "cognitive functional therap*" |
| S59 | (MH "Dialectical Behavior Therapy") |
| S58 | "dialectical behavio* therap*" |
| S57 | "focus#ed therap*" |
| S56 | (MH "Psychotherapy") |
| S55 | psychotherap* OR "psychotherapeutic intervention*" |
| S54 | (MH "Hypnosis") |
| S53 | hypno* |
| S52 | (MH "Acceptance and Commitment Therapy") |
| S51 | "acceptance and commitment therap*" |
| S50 | (MH "Motivational Interviewing") |
| S49 | "motivation* interview*" |
| S48 | (MH "Mindfulness") |
| S47 | mindfulness |
| S46 | (MH "Cognitive Therapy") |
| S45 | (MH "Behavior Therapy") |
| S44 | "cognitive behavio* therap*" OR "behavio* therap*" OR "cognitive therap*" |
| S43 | S1 OR S2 OR S3 OR S4 OR S5 OR S6 OR S7 OR S8 OR S9 OR S10 OR S11 OR S12 OR S13 OR S14 OR S15 OR S16 OR S17 OR S18 OR S19 OR S20 OR S21 OR S22 OR S23 OR S24 OR S25 OR S26 OR S27 OR S28 OR S29 OR S30 OR S31 OR S32 OR S33 OR S34 OR S35 OR S36 OR S37 OR S38 OR S39 OR S40 OR S41 OR S42 |
| S42 | (MH "Tennis Elbow") |
| S41 | "tennis elbow" |
| S40 | (MH "Whiplash Injuries") |
| S39 | whiplash |
| S38 | (MH "Raynaud's Disease") |
| S37 | raynaud* |
| S36 | (MH "Burning Mouth Syndrome") |
| S35 | "burning mouth syndrome" |
| S34 | (MH "Carpal Tunnel Syndrome") |
| S33 | (MH "Cumulative Trauma Disorders") |
| S32 | "cumulative trauma disorder*" OR "repetitive strain injur*" OR "carpal tunnel syndrome*" |
| S31 | (MH "Irritable Bowel Syndrome") |
| S30 | "irritable bowel syndrome" OR "irritable bowel disease*" |
| S29 | (MH "Endometriosis") |
| S28 | endometriosis |
| S27 | (MH "Trigeminal Neuralgia") |
| S26 | (MH "Neuralgia") |
| S25 | neuralgia OR "trigeminal neuralgia" |
| S24 | (MH "Vulvodynia") |
| S23 | vulvodynia |
| S22 | (MH "Osteoarthritis") |
| S21 | osteoarthritis |
| S20 | (MH "Arthritis") |
| S19 | arthritis |
| S18 | (MH "Migraine") |
| S17 | migraine |
| S16 | (MH "Headache") |
| S15 | headache |
| S14 | fibrositis |
| S13 | (MH "Fibromyalgia") |
| S12 | fibromyalgia |
| S11 | backache |
| S10 | "musculoskeletal disorder*" |
| S9 | (MH "Musculoskeletal Pain") |
| S8 | (MH "Complex Regional Pain Syndromes") |
| S7 | (MH "Sciatica") |
| S6 | (MH "Low Back Pain") |
| S5 | (MH "Back Pain") |
| S4 | (MH "Pain") |
| S3 | pain OR "back pain" OR "neuropathic pain" OR "complex regional pain" OR "musculoskeletal pain" OR "sciatic* pain" |
| S2 | (MH "Chronic Pain") |
| S1 | "chronic pain" OR chronic N3 pain |

**Supplementary file 4: linked RCT papers**

- 1. Andersen, L. N., Juul-Kristensen, B., Roessler, K. K., Herborg, L. G., Sorensen, T. L., & Sogaard, K. (2013). Efficacy of 'tailored physical activity' or 'chronic pain self-management program' on return to work for sick-listed citizens: Design of a randomised controlled trial. *BMC Public Health*, *13.*
  2. Andersen, L. N., Juul-Kristensen, B., SÃrensen, T. L., Herborg, L. G., Roessler, K. K., & SÃgaard, K. (2016). Longer term follow-up on effects of tailored physical activity or chronic pain self-management programme on return-to-work: A randomized controlled trial. *Journal of Rehabilitation Medicine*, *48*(10), 887-892.
  3. Bendix, A. E., Bendix, T., Haestrup, C., & Busch, E. (1998a). A prospective, randomized 5-year follow-up study of functional restoration in chronic low back pain patients. *European Spine Journal,* *7*(2), 111–119.
  4. Bendix, A. F., Bendix, T., Labriola, M., & Boekgaard, P. (1998b). Functional restoration for chronic low back pain - two-year follow-up of two randomized clinical trials. *Spine*, *23*(6), 717–725.
  5. Bendix, A. F., Bendix, T., Lund, C., Kirkbak, S., & Ostenfeld, S. (1997). Comparison of three intensive programs for chronic low back pain patients: A prospective, randomized, observer-blinded study with one-year follow-up. *Scandinavian Journal of Rehabilitation Medicine*, *29*(2), 81–89.
  6. Blödt, S., Pach, D., Roll, S., & Witt, C. M. (2014). Effectiveness of app-based relaxation for patients with chronic low back pain (relaxback) and chronic neck pain (relaxneck): Study protocol for two randomized pragmatic trials. *Trials, 15(*1), 490.
  7. Brendbekken, R., Harris, A., Ursin, H., Eriksen, H. R., & Tangen, T. (2016). Multidisciplinary intervention in patients with musculoskeletal pain: A randomized clinical trial. *International Journal of Behavioral Medicine*, *23*(1), 1–11.
  8. Brendbekken, R., Vaktskjold, A., Harris, A., & Tangen, T. (2018). Predictors of return-to-work in patients with chronic musculoskeletal pain: A randomized clinical trial. *Journal of Rehabilitation Medicine,* *50*(2), 193–199.
  9. Brox, J. I., Nygaard, Ã, Holm, I., Keller, A., Ingebrigtsen, T., & ReikerÃ¥s, O. (2010). Four-year follow-up of surgical versus non-surgical therapy for chronic low back pain. *Annals of the Rheumatic Diseases*, *69*(9).
  10. den Hollander, M., Goossens, M., de Jong, J., Ruijgrok, J., Oosterhof, J., Onghena, P., . . . Vlaeyen, J. W. S. (2016). Expose or protect? A randomized controlled trial of exposure in vivo vs pain-contingent treatment as usual in patients with complex regional pain syndrome type 1. *Pain, 157*(10), 2318-2329.
  11. DRKS00010820. Effectiveness of an internet- and mobile-based treatment of comorbid depression in chronic back pain patients on sick leave. Retrieved from https://www.cochranelibrary.com/central/doi/10.1002/central/CN-01853615/full
  12. DRKS00015465. A combined pain competence and depression prevention training in inpatient multidisciplinary rehabilitation for chronic low back pain (debora I). Retrieved from https://www.cochranelibrary.com/central/doi/10.1002/central/CN-01907485/fullhttps://www.cochranelibrary.com/central/doi/10.1002/central/CN-01907485/full.
  13. Ehrenborg, C., Gustafsson, S., & Archenholtz, B. (2014). Long-term effect in ADL after an interdisciplinary rehabilitation programme for WAD patients: A mixed-method study for deeper understanding of participants' programme experiences. *Disability and Rehabilitation, 36*(12), 1006–1013.
  14. Friedrich, M., Gittler, G., Arendasy, M., & Friedrich, K. M. (2005). Long-term effect of a combined exercise and motivational program on the level of disability of patients with chronic low back pain. *Spine,* *30*(9), 995–1000.
  15. Froholdt, A., Reikeraas, O., Holm, I., Keller, A., & Brox, J. I. (2012). No difference in 9-year outcome in CLBP patients randomized to lumbar fusion versus cognitive intervention and exercises. *European Spine Journal,* *21*(12), 2531-2538.
  16. Going, S. (2012). Community-based programs for improving physical function in people with early knee osteoarthritis. Retrieved from <https://clinicaltrials.gov/study/NCT00586300>
  17. Graded exposure (GEXP) in vivo versus physiotherapy in complex regional pain syndrome type I (CRPS-I). (2019) ClinicalTrials.Gov, Retrieved from https://clinicaltrials.gov/study/NCT00625976
  18. Hagen, E. M., Eriksen, H. R., & Ursin, H. (2000). Does early intervention with a light mobilization program reduce long-term sick leave for low back pain? *Spine, 25*(15), 1973–1976.
  19. Haldorsen, E., Grasdal, A. L., Skouen, J. S., Risa, A. E., Kronholm, K., & Ursin, H. (2002). Is there a right treatment for a particular patient group? comparison of ordinary treatment, light multidisciplinary treatment, and extensive multidisciplinary treatment for long-term sick-listed employees with musculoskeletal pain. *Pain, 95*(1-2), 49–63.
  20. Härkäpää, K., Järvikoski, A., Mellin, G., & Hurri, H. (1989). A controlled study on the outcome of inpatient and outpatient treatment of low back pain. part I. pain, disability, compliance, and reported treatment benefits three months after treatment. *Scandinavian Journal of Rehabilitation Medicine*, *21*, 81–9.
  21. Harris, A., Moe, T. F., Eriksen, H. R., Tangen, T., Lie, S. A., Tveito, T. H., & Reme, S. E. (2017). Brief intervention, physical exercise and cognitive behavioural group therapy for patients with chronic low back pain (the CINS trial). *European Journal of Pain, 21*(8), 1397–1407.
  22. Haugli, L., Steen, E., Laerum, E., Nygard, R., & Finset, A. (2001). Learning to have less pain - is it possible? A one-year follow-up study of the effects of a personal construct group learning programme on patients with chronic musculoskeletal pain. *Patient Education and Counseling,* *45(*2), 111–118.
  23. Haugli, L., Steen, E., Laerum, E., Nygard, R., & Finset, A. (2003). Psychological distress and employment status. effects of a group learning programme for patients with chronic musculoskeletal pain. *Psychology, Health & Medicine*, 8(2), 135–148.
  24. Haugmark, T., Hagen, K. B., Provan, S. A., Baerheim, E., & Zangi, H. A. (2018). Effects of a community-based multicomponent rehabilitation programme for patients with fibromyalgia: Protocol for a randomised controlled trial. *BMJ Open*, *8*(6).
  25. Hutting, N., Staal, J. B., Heerkens, Y. F., Engels, J. A., & Nijhuis-van der Sanden, M. (2013). A self-management program for employees with complaints of the arm, neck, or shoulder (CANS): Study protocol for a randomized controlled trial. *Trials*, *14.*
  26. Indahl, A., Velund, L., & Reikeraas, O. (1995). Good prognosis for low back pain when left untampered. A randomized clinical trial. *Spine, 20*(4), 473–477.
  27. Jensen, I. B., Bergstrom, G., Ljungquist, T., & Bodin, L. (2005). A 3-year follow-up of a multidisciplinary rehabilitation programme for back and neck pain. *Pain, 115*(3), 273–283.
  28. Kool, J., Bachmann, S., Oesch, P., Knuesel, O., Ambergen, T., de Bie, R., & van den Brandt, P. (2007). Function-centered rehabilitation increases work days in patients with nonacute nonspecific low back pain: 1-year results from a randomized controlled trial. *Archives of Physical Medicine and Rehabilitation, 88*(9), 1089–1094.
  29. Kopnick, A., & Hampel, P. (2020). Influence of social status on the success of rehabilitation among patients with chronic low back pain results of a 2-year follow-up after inpatient multidisciplinary rehabilitation. *Rehabilitation*, *59*(06), 348–356.
  30. Kroese, M. (2006). Fibromyalgia on the move. randomised study on the effect and cost-effectiveness of a multidisciplinary part-time daycare intervention. ISRCTN, doi:<https://doi.org/10.1186/ISRCTN32542621>
  31. Lambeek, L. C., Anema, J. R., van Royen, B.,J., Buijs, P. C., Wuisman, P. I., van Tulder, M.,W., & van Mechelen, W. (2007). Multidisciplinary outpatient care program for patients with chronic low back pain: Design of a randomized controlled trial and cost-effectiveness study [ISRCTN28478651]. *BMC Public Health*, *7*, 254.
  32. Lin, J. X., Sander, L., Paganini, S., Schlicker, S., Ebert, D., Berking, M., . . . Baumeister, H. (2017). Effectiveness and cost-effectiveness of a guided internet- and mobile-based depression intervention for individuals with chronic back pain: Protocol of a multi-centre randomised controlled trial. *BMJ Open*, *7*(12).
  33. Miller, J., MacDermid, J. C., Walton, D. M., & Richardson, J. (2015a). Chronic pain self-management support with pain science education and exercise (COMMENCE): Study protocol for a randomized controlled trial. *Trials, 16* .
  34. Miller et al., (2015b). Chronic pain self-management support with pain education and exercise. NCT02422459. Retrieved from https://www.cochranelibrary.com/central/doi/10.1002/central/CN-02033034/full
  35. NCT01475591. Individually tailored web-based multimodal pain rehabilitation in primary health care. Retrieved from https://www.cochranelibrary.com/central/doi/10.1002/central/CN-02033157/fullhttps://www.cochranelibrary.com/central/doi/10.1002/central/CN-02033157/full
  36. NCT03141541. Pain management for patients with low back pain and psychosocial risk factors in a hospital setting. Retrieved from https://www.cochranelibrary.com/central/doi/10.1002/central/CN-01580941/full
  37. Neumann, A., & Hampel, P. (2022). Long-term effects of rehabilitation and prevention of further chronification of pain among patients with non-specific low back pain. *Journal of Back and Musculoskeletal Rehabilitation*, 35(6), 1257–1268.
  38. Oesch, P. R., Kool, J. P., Bachmann, S., & Devereux, J. (2006). The influence of a functional capacity evaluation on fitness for work certificates in patients with non-specific chronic low back pain. Work: *Journal of Prevention, Assessment & Rehabilitation*, *26*(3), 259–271.
  39. Reme, S. E., Tveito, T. H., Chalder, T., Bjorkkjaer, T., Indahl, A., Brox, J. I., . . . Eriksen, H. R. (2011). Protocol for the cognitive interventions and nutritional supplements (CINS) trial: A randomized controlled multicenter trial of a brief intervention (BI) versus a BI plus cognitive behavioral treatment (CBT) versus nutritional supplements for patients with long-lasting muscle and back pain. *BMC Musculoskeletal Disorders, 12* .
  40. Reme, S. J., & Chalder, T. (2007). Manual for terapeuter: Kognitiv atferdsterapi for sykmeldte pasienter med langvarige ryggsmerter. Unpublished manuscript.
  41. Rolving, N., Oestergaard, L. G., Willert, M. V., Christensen, F. B., Blumensaat, F., Bunger, C., & Nielsen, C. V. (2014). Description and design considerations of a randomized clinical trial investigating the effect of a multidisciplinary cognitive-behavioural intervention for patients undergoing lumbar spinal fusion surgery. *BMC Musculoskeletal Disorders, 15*
  42. Sander, L., Paganini, S., Lin, J., Schlicker, S., Ebert, D. D., Buntrock, C., & Baumeister, H. (2017). Effectiveness and cost-effectiveness of a guided internet- and mobile-based intervention for the indicated prevention of major depression in patients with chronic back pain—study protocol of the PROD-BP multicenter pragmatic RCT. *BMC Psychiatry, 17*(1), 36.
  43. Skouen, J. S., & Kvåle, A. (2009). Different outcomes in subgroups of patients with long-term musculoskeletal pain. *Norsk Epidemiologi*, *16*(2).
  44. Skovbo, M. H., Agerbo, K., Jakobsen, A., Clausen, S. A., Langagergaard, V., & Rolving, N. (2021). Description of content, structure and theoretical model of a group-based pain management programme in the treatment of patients with persistent non-specific low back pain and psychological risk factors in a secondary sector setting. *Clinical Rehabilitation, 35*(8), 1077–1088.

Supplementary file 5: RCT intervention description and congruence analysis

| **First author Reference** | **Main intervention components** | **Agreed intervention functions present** | **Agreed TDF domains present** | **Agreed BCTs present** | **Agreed level of congruence:**  **1 = Little or no congruence**  **2 =Some congruence**  **3 = Good congruence** |
| --- | --- | --- | --- | --- | --- |
| Alaranta 1994 (Intervention) | Intervention 1 = Cognitive behavioural disability management (mixed psychological) plus exercise | **Education**  **Persuasion**  **Training (3)** | **Knowledge**  **Skills**  **Beliefs capabilities**  **Memory/attention/decision making**  **Emotion (5)** | **1.2 problem solving**  **3.1 soc supp unspecified**  **4.1 instructions perform behaviour**  **6.1 Demo**  **Behaviour**  **8.1 Behaviour practice/rehearsal**  **11.2 Reduce**  **Negative emotions**  **12.6 body changes (7)** | 2 |
|  |  |  |  |  |  |
| Alaranta 1994 (Comparator) | Comparator 1 =exercise, education (back school), passive physical therapy | **Education**  **Training**  **Enablement (3)** | **Knowledge**  **Skills (2)** | **4.1 Instructions perform behaviour**  **8.1 Behaviour practice/rehearsal**  **12.6 body changes (3)** | **2** |
|  |  |  |  |  |  |
| Altmaier 1992 (Intervention) | Intervention 1 Mixed psychological including cognitive behavioural coping skills and counselling, plus education (mechanisms of pain), exercise, medical treatment, vocational rehabilitation | **Education**  **Incentivisation**  **Persuasion**  **Training**  **Environmental restructuring (5)** | **Knowledge**  **Skills**  **Beliefs capability**  **Reinforcement**  **Environmental context resources**  **Social influence**  **Behavioural regulation (7)** | **2.2 Feedback on behaviour**  **2.3 self-monitoring behaviour**  **2.6 biofeedback**  **3.1 social support unspecified**  **4.1 instruction on performing behaviour**  **6.1 demonstration behaviour**  **8.1 Behavioural practice/rehearsal**  **10.4 social reward**  **11.2 reduce negative emotions**  **12.5 add objects to environment**  **12.6 Body changes**  **(11)** | **3** |
|  |  |  |  |  |  |
| Altmaier 1992 (intervention) | Intervention 2 - counselling, plus education (mechanisms of pain), exercise, medical treatment, vocational rehabilitation | **Education**  **Training (2)** | **Knowledge**  **Skills**  **Social influence (3)** | **3.1 social support unspecified**  **4.1 instructions on performing behaviour**  **6.1 Demonstration of the behaviour**  **8.1 Behavioural practice/rehearsal**  **11.2 Reduce negative emotions**  **12.6 Body changes (6)** | **2** |
|  |  |  |  |  |  |
| Anderson 2015 (Intervention) | Intervention 1 self-management including self-efficacy training, plus education and health guidance | **Education**  **Persuasion**  **(2)** | **Knowledge**  **Skills**  **Beliefs about capabilities**  **Goals**  **Social influence**  **Behavioural regulation (6)** | **1.1 Goal setting (outcome)**  **3.1 social support unspecified**  **4.1 instructions on performing behaviour**  **9.1 credible source**  **11.2 reduce negative emotions**  **12.6 body changes (6)** | **2** |
|  |  |  |  |  |  |
| Anderson 2015 (Comparator1) | Comparator 1 - Exercise and health guidance | **Education**  **Persuasion**  **Training (3)** | **Knowledge**  **Skills**  **Goals**  **Social influence**  **Behavioural regulation (5)** | **1.3 Goal setting outcome**  **2.6 Biofeedback**  **3.1 social support unspecified**  **4.1 instructions perform behaviour**  **6.1 Demonstration behaviour**  **8.1 Behavioural practice**  **8.7 graded tasks**  **9.1 credible source**  **12.6 body changes (9)** | **3** |
|  |  |  |  |  |  |
| Andersen 2015 (Comparator 2) | Comparator 2 - health guidance | **Education**  **Persuasion (2)** | **Knowledge**  **Goals**  **Social influence (3)** | **1.3 goal setting outcome**  **3.1 social support unspecified**  **(2)** | **2** |
|  |  |  |  |  |  |
| Baumeister 2021 (Intervention) | Intervention 1 Mixed psychological (CBT based including psychoeducation), plus exercise and medical treatment. | **Education**  **Persuasion**  **Incentivisation**  **Training**  **Environmental restructuring**  **Enablement (6)** | **Knowledge**  **Skills**  **Beliefs about capabilities**  **Goals**  **Environmental context and resources**  **Social influence**  **Emotion**  **Behavioural regulation (7)** | **1.1 Goal setting behaviour**  **1.2 problem solving**  **1.3 Goal setting outcome**  **2.2 Feedback on behaviour**  **2.3 self-monitoring of behaviour**  **2.4 self-monitoring of outcomes of behaviour**  **3.1 social support unspecified**  **4.1 instructions perform behaviour**  **5.4 monitoring emotional consequences of behaviour**  **7.1 prompts/cues**  **8.1 behavioural practice**  **9.1 credible source**  **11.2 reduce negative emotions**  **15.3 focus on past success**  **(14)** | **3** |
|  |  |  |  |  |  |
| Baumeister 2021 (Control) | Control TAU - medical treatment | **Education**  **Training**  **Enablement (3)** | **Knowledge**  **Skills**  **Social influence**  **Emotion (4)** | **3.1 social support unspecified**  **4.1 instructions perform behaviour**  **11.1 pharmacological support**  **11.2 reduce negative emotions**  **(4)** | **2** |
|  |  |  |  |  |  |
| Bendix 2000 (Intervention) | Intervention 1 - mixed psychological (eg psychoeducation, biofeedback) plus exercise and occupational therapy/ergonomics. | **Education persuasion**  **Training**  **Environmental restructuring**  **Enablement (5)** | **Knowledge**  **Skills**  **Beliefs capabilities**  **Goals**  **Environmental context and resource**  **Social influence (6)** | **1.3 goal setting outcome**  **2.6 biofeedback**  **3.1 social support unspecified**  **4.1 instructions perform behaviour**  **6.1 Demonstration of behaviour**  **8.1 Behaviour practice**  **9.1 credible source**  **11.2 reduce negative emotions**  **12.6 body changes**  **13.2 framing/reframing**  **(10)** | **2** |
|  |  |  |  |  |  |
| Bendix 2000 (Comparator) | Comparator 1: Exercise | **Training (1)** | **Skills (1)** | **4.1 instructions perform behaviour**  **6.1 demonstration behaviour**  **8.1 Behaviour practice**  **12.6 body changes (4)** | **2** |
|  |  |  |  |  |  |
| Bendix 1996 (Intervention) | Intervention 1- mixed psychological (eg biofeedback, counselling, psychoeducation), plus exercise and occupational therapy/ergonomics. | **Education**  **Persuasion**  **Training**  **Modelling**  **Enablement (5)** | **Knowledge**  **Skills**  **Beliefs capabilities**  **Goals**  **Environmental context resources**  **Social influences (6)** | **1.3 goal setting outcome**  **2.6 biofeedback**  **3.1 social support unspecified**  **3.2 social support practical**  **4.1 instructions perform behaviour**  **6.1 demonstration behaviour**  **8.1 behaviour practice**  **8.7 graded tasks**  **9.1 credible source**  **10.9 self reward**  **11.2 reduce negative emotions**  **12.6 body changes**  **13.2 framing/reframing**  **(13)** | **3** |
|  |  |  |  |  |  |
| Bendix 1996 (Control) | Control: TAU | **0** | **0** | **0** | **NA** |
|  |  |  |  |  |  |
| Bendix 1995 (Intervention1) | Intervention 1 (IMT) -mixed psychological (including counselling, psychoeducation) plus ergonomics, and exercise | **Education**  **Persuasion**  **Training**  **Modelling**  **Enablement (5)** | **Knowledge**  **Skills**  **Beliefs capabilities**  **Goals**  **Environmental context resources**  **Social influence**  **Emotion (7)** | **1.3 goal setting outcome**  **2.6 biofeedback**  **3.1 social support unspecified**  **3.2 social support practical**  **4.1instructions perform behaviour**  **6.1 demonstration of behaviour**  **8.1 Behaviour practice rehearsal**  **8.7 graded tasks**  **9.1 credible source**  **11.2 reduce negative emotions**  **12.6 body changes**  **13.2 framing/reframing**  **(12)** | **3** |
|  |  |  |  |  |  |
| Bendix 1995 (Intervention2) | Intervention 2 (PPP) - psychological pain management - mixed psychological (including psychoeducation), plus exercise | **Education**  **Training**  **(2)** | **Knowledge**  **Skills (2)** | **1.3 goal setting outcome**  **4.1 instructions perform behaviour**  **6.1 demonstration behaviour**  **8.1 behaviour practice**  **8.7 graded tasks**  **12.6 body changes (6)** | **3** |
|  |  |  |  |  |  |
| Bendix 1995 (Comparator) | Comparator 1 Exercise | **Education**  **Training (2)** | **Knowledge**  **Skills (2)** | **4.1 instructions perform behaviour**  **6.1 demonstration behaviour**  **8.1 behaviour practice**  **8.7 graded tasks**  **12.6 body changes (5)** | **3** |
|  |  |  |  |  |  |
| Brendbekken 2017 (Intervention 1) | Intervention 1 - MI = Counselling and Physical Examination; working conditions | **Education**  **Persuasion**  **Enablement (3)** | **Knowledge**  **Skills**  **Beliefs capabilities**  **Social influence**  **Behavioural regulation (5)** | **1.2 problem solving**  **2.7 feedback on outcomes of behaviour**  **3.1 social support unspecified**  **9/1 credible source**  **11.2 reduce negative emotions**  **(5)** | **2** |
|  |  |  |  |  |  |
| Brendbekken 2017 (Intervention 2) | Intervention 2 - BI = Mixed psychological (eg, Counselling) plus Physical Examination | **Education**  **Persuasion**  **Training**  **Enablement (4)** | **Knowledge**  **Skills**  **Beliefs about capabilities**  **Optimism**  **Beliefs consequences**  **Goals (6)** | **1.3 goal setting outcome**  **2.6 biofeedback**  **4. 1 instructions perform behaviour**  **5.1 information health consequences**  **6.1 demonstration behaviour**  **9.1 credible source**  **(6)** | **2** |
|  |  |  |  |  |  |
| Brox 2003 (Intervention) | Intervention 1 Mixed psychological (eg. Psychoeducation) plus exercises | **Education**  **Persuasion**  **Training**  **Enablement (4)** | **Knowledge**  **Skills**  **Beliefs consequences**  **Reinforcement**  **Goals**  **Social influence**  **Behavioural regulation**  **Beliefs about capabilities (8)** | **1.3 goal setting behaviour**  **2.3 self-monitoring of behaviour**  **2.6 biofeedback**  **2.7 Feedback on outcomes of behaviour**  **3.1 social support unspecified**  **4.1 instructions perform behaviour**  **5.1 info health consequences**  **6.1 demo behaviour**  **8.1 Behaviour practice/rehearsal**  **8.7 graded tasks**  **9.1 credible source**  **12.6 body changes**  **13.2 Framing/reframing**  **(13)** | **3** |
|  |  |  |  |  |  |
| Brox 2003 (Comparator) | Comparator 1 - medical treatment | **Enablement (1)** |  | **4.1 instructions perform behaviour**  **9.1 credible source**  **12.6 body changes (3)** | **2** |
|  |  |  |  |  |  |
| Brox 2006 (Intervention) | Intervention 1 Mixed psychological (eg. Fear avoidance) plus exercise | **Education**  **Persuasion**  **Training**  **Enablement (4)** | **Knowledge**  **Skills**  **Beliefs capabilities**  **Beliefs consequences**  **Reinforcement**  **Goals**  **Social influences**  **Behavioural regulation (8)** | **1.3 goal setting outcome**  **2.6 biofeedback**  **2.7 feedback on outcomes of behaviour**  **Self -monitoring of behaviour**  **3.1 social support unspecified**  **4.1 instructions perform behaviour**  **5.1 information health consequences**  **6.1 Demonstration of behaviour**  **8.1 Behaviour practice**  **8.7 Graded tasks**  **9.1 credible source**  **12.6 body changes**  **13.2 Framing/reframing (13)** | **2** |
|  |  |  |  |  |  |
| Brox 2006 (Comparator) | Comparator 1 Medical Treatment | **Enablement (1)** |  | **4.1 Instructions perform behaviour**  **9.1 Credible source**  **12.6 body changes (3)** | **2** |
|  |  |  |  |  |  |
| Calner 2017 (Intervention 1) | Intervention 1 WBI and MMR -mixed psychological (eg cognitive behavioural counselling, mindfulness, action planning) plus ergonomics, medical treatment, education, passive therapy and exercise. | **Education**  **Persuasion**  **Training**  **Enablement (4)** | **Knowledge**  **Skills**  **Goals**  **Memory, attention, dec making,**  **Social influence**  **Emotion,**  **Behavioural regulation (7)** | **1.2 Problem solving**  **1.3 Goal setting outcome**  **1.4 action planning**  **1.8 behavioural contract**  **2.3 self monitoring of behaviour**  **2.7 feedback on outcomes of behaviour**  **3.1 social support unspecified**  **3.2 social support practical**  **4.1 instructions perform behaviour**  **8.1 Behaviour practice**  **11.2 reduce negative emotions**  **12.6 body changes (12)** | **3** |
|  |  |  |  |  |  |
| Calner 2017 (Intervention 2) | Intervention 2 MMR only -mixed psychological (eg cognitive behavioural counselling, mindfulness) plus ergonomics, medical treatment, education, passive therapy. | **Education**  **Training**  **Enablement (3)** | **Knowledge**  **Skills**  **Goals**  **Memory, attention, dec making**  **Social influence**  **Emotion**  **Behavioural**  **Regulation (7)** | **1.2 problem solving**  **1.3 goal setting outcome**  **1.4 action planning**  **1.8 behavioural contract**  **2.7 feedback on outcomes of behaviour**  **3.1 social support unspecified**  **3.2 social support practical**  **4.1 instructions perform behaviour**  **8.1 Behavioural practice**  **12.6 body changes (10)** | **3** |
|  |  |  |  |  |  |
| Den Hollander 2018 (Intervention) | Intervention 1 Exposure In Vivo | **Education**  **Persuasion**  **Enablement (3)** | **Knowledge**  **Beliefs capabilities**  **Beliefs consequences**  **ECR**  **Emotion**  **Behavioural regulation (6)** | **3.1 social support unspecified**  **3.3 social support emotional**  **4.1 instructions perform behaviour**  **4.4 behavioural experiments**  **5.1 information health consequences**  **6.1 demonstration of behaviour**  **7.7 exposure**  **8.1 behavioural practice/rehearsal**  **11.2 reducing negative emotions**  **13.2 Framing/reframing**  **(10)** | **3** |
|  |  |  |  |  |  |
| Den Hollander 2018 (Comparator) | Comparator 1 Pain-Contingent Physical Therapy including exercise, passive therapy, plus ergonomics | **Education**  **Training**  **Enablement (3)** | **Knowledge**  **Skills**  **(2)** | **4.1 instructions perform behaviour**  **8.1 Behavioural practice**  **(2)** | **2** |
| Ehrenborg (2010) (intervention 1 without biofeedback) | Intervention 1 - conventional interdisciplinary programme without Biofeedback - Mixed psychology (psychoeducation, relaxation, stress management) plus exercise and ergonomics | **Education**  **Training**  **Environmental restructuring**  **Enablement (4)** | **Knowledge**  **Skills**  **Beliefs capabilities**  **Goals**  **Environment context resources**  **Social influence**  **Emotion (7)** | **1.2 problem solving**  **1.3 Goal Setting (outcome)**  **3.1 Social support (unspecified)**  **4.1 Instruction to perform behaviour**  **8.1 Beh practice / rehearsal**  **11.2 Reduce negative emotions**  **12.1 Restructuring physical environment**  **12.6 body changes (8)** | **2** |
| Ehrenborg (2010) (intervention 2 with biofeedback) | Intervention 2 - conventional interdisciplinary programme with Biofeedback - Mixed psychology (biofeedback, psychoeducation, relaxation, stress management) plus exercise and ergonomics | **Education**  **Training**  **Environmental restructuring**  **Enablement (4)** | **Knowledge**  **Skills**  **Beliefs capabilities**  **Goals**  **Environmental context resources**  **Social influence**  **Emotion (7)** | **1.2 problem solving**  **1.3 Goal Setting (outcome)**  **2.6 Biofeedback**  **3.1 Social support (unspecified)**  **4.1 Instruction to perform behaviour**  **8.1 Behavioural practice / rehearsal**  **11.2 Reduce negative emotions**  **12.1 Restructuring physical environment (8)** | **2** |
|  |  |  |  |  |  |
| Friedrich 1998 (Intervention) | Intervention 1 Motivational counselling (including positive reinforcement) plus exercise, | **Education**  **Persuasion**  **Inccentivisation**  **Enablement**  **Training**  **Environmental restructuring (6)** | **Knowledge**  **Skills**  **Beliefs capabilities**  **Beliefs consequences**  **Reinforcement**  **Goals**  **Environmental context resources**  **Emotion**  **Behavioural**  **Regulation (9)** | **1.2 problem solving**  **1.4 action planning**  **1.8 behavioural contract**  **2.2 feedback on behaviour**  **2.3 self-monitoring of behaviour**  **2.5 monitoring outcomes of behaviour**  **3.2 social support practical**  **4.1 instructions perform behaviour**  **5.1 information about health consequences6.1 demonstration of behaviour**  **7.1 Prompts/cues**  **8.1 Behavioural practice**  **8.7 Graded tasks**  **10.1 Material incentive behaviour**  **10.3 non specific reward**  **10.4 social reward**  **10.6 non specific incentive**  **10.7 self incentive**  **10.9 self-reward**  **11.2 reduce negative emotions**  **12.5 add objects to the environment**  **12.6 body changes**  **(22)** | **3** |
|  |  |  |  |  |  |
| Friedrich 1998 (Comparator) | Comparator -exercise | **Education**  **Persuasion**  **Training (3)** | **Knowledge**  **Skills**  **Beliefs consequences**  **Emotion (4)** | **2.2 Feedback on behaviour**  **2.5 monitoring outcomes of behaviour**  **3.2 social support practical**  **4.1 instructions perform behaviour**  **5.1 information health consequences**  **6.1 demonstration behaviour**  **8.1 behavioural practice/rehearsal**  **8.7 Graded tasks**  **11.2 reduce negative emotions**  **12.6 body changes (10)** | **3** |
|  |  |  |  |  |  |
| Gustavsson 2006 (intervention ) | Intervention 1 mixed psychological (including Applied Relaxation (using progressive and autogenic relaxation methods and cue-controlled relaxation), psychoeducation (pain and stress management)) plus TAU - could include: passive therapy, exercise, medical treatment, | **Education**  **Training (2)** | **Knowledge**  **Skills**  **Emotion(3)** | **4.1 instructions perform behaviour**  **6.1 demonstration behaviour**  **8.1 Behaviour practice**  **8.6 Generalisation of target behaviour**  **8.7 Graded tasks**  **11.2 reduce negative emotions**  **12.6 body changes (7)** | **2** |
|  |  |  |  |  |  |
| Gustavsson 2006 (control) | Control TAU - could include: passive therapy, exercise, medical treatment, | **Enablement (1)** | **0** | **12.6 body changes (1)** | **1** |
|  |  |  |  |  |  |
| Hampel 2019 Intervention 1 (pain competence) | Intervention 1 (pain competence) mixed psychological (e.g. psychoeducation, cognitive-behavioural exercises) | **Education**  **Persuasion**  **Training**  **Enablement (4)** | **Knowledge**  **Skills**  **Beliefs capabilities**  **Memory, attention, decision making,**  **Social influences**  **Emotion (6)** | **1.2 problem solving**  **2.2 feedback on behaviour**  **3.1 social support unspecified**  **4.1 instructions perform behaviour**  **6.1 demonstration of behaviour**  **6.2 social comparison**  **8.1 Behavioural practice**  **11.2 reduce negative emotions**  **12. 6 body changes**  **13.2 framing/reframing**  **(10)** | **3** |
|  |  |  |  |  |  |
| Hampel 2019 Intervention 2 (combined - pain competence and depression prevention | Intervention 2 ( (combined - pain competence and depression prevention ) mixed psychological (e.g. psychoeducation, cognitive-behavioural exercises and cog behavioural depression prevention) | **Education**  **Persuasion**  **Training**  **Enablement (4)** | **Knowledge**  **Skills**  **Beliefs capabilities**  **Memory, attention, decision making,**  **Social influences**  **Emotion (6)** | **1.2 problem solving**  **2.2 feedback on behaviour**  **2.3 self-monitoring of behaviour**  **3.1 social support unspecified**  **4.1 instructions perform behaviour**  **5.1 information health consequences**  **6.1 demonstration of behaviour**  **6.2 social comparison**  **8.1 Behavioural practice**  **11.2 Reduce negative emotions**  **12.6 body changes**  **13.2 framing/reframing**  **(12)** | **3** |
|  |  |  |  |  |  |
| Harkapaa 1989 (Intervention 1 inpatient) | Intervention 1 (inpatient) self-care - problem solving plus exercise,, education and passive therapy | **Education**  **Training**  **Enablement (3)** | **Knowledge**  **Skills**  **ECR**  **Social influences**  **(4)** | **2.1 monitor behaviour by others without feedback**  **2.2 feedback on behaviour**  **4.1 instructions perform behaviour**  **6.1 demonstration of behaviour**  **8.1 Behavioural practice**  **9.1 Credible source**  **12.6 body changes (7)** | **2** |
|  |  |  |  |  |  |
| Harkapaa 1989 (Intervention 2 outpatient) | Intervention 2 (outpatient) self-care - problem solving plus exercise,, education and passive therapy | **Education**  **Training**  **Enablement (3)** | **Knowledge**  **Skills**  **ECR**  **Social influences (4)** | **2.1 monitoring of behaviour by others without feedback**  **2.2 feedback on behaviour**  **4.1 instructions perform behaviour**  **6.1 demonstration of behaviour**  **8.1 behavioural practice**  **9.1 credible source**  **12.6 body changes (7)** | **2** |
|  |  |  |  |  |  |
| Harkapaa 1989 (Comparator) | Comparator - back exercises instructions and ergonomics | **Education (1)** | **Knowledge (1)** | **4.1 instructions perform behaviour**  **(1)** | **2** |
|  |  |  |  |  |  |
| Haugli 2000b (Intervention 1) | Intervention 1 - mixed psychological (e.g. counselling (MI), guided imagery and mindfulness) | **Education**  **Training**  **Enablement (3)** | **Knowledge**  **Skills**  **Beliefs capabilities**  **ECR**  **Social influences**  **Emotions (6)** | **1.2 problem solving**  **3.1 social support unspecified**  **5.1 information health consequences**  **8.1 behavioural practice**  **9.1 credible source**  **11.2 reduce negative emotions**  **12.6 body changes**  **13. 2 framing/reframing**  **13.5 identity associated with changed behaviour**  **(9)** | **2** |
|  |  |  |  |  |  |
| Haugli 2000 (Control) | Control: TAU - could include medical treatment | **Enablement (1)** | **0** | **0** | **NA** |
|  |  |  |  |  |  |
| Haugmark 2021 (Intervention 1) | Intervention 1 - mixed psychological (e.g. counselling (MI), guided imagery and mindfulness) | **Education**  **Persuasion**  **Training**  **Enablement (4)** | **Knowledge**  **Skills**  **Beliefs capabilities**  **Beliefs consequences**  **Goals**  **Environmental context resources**  **Social influences**  **Emotion**  **Behavioural regulation (9)** | **1.2 problem solving**  **1.3 goal setting outcome**  **1.4 action planning**  **2.3 self-monitoring of behaviour**  **2.4 self monitoring of outcomes of behaviour**  **3.1 social support unspecified**  **4.1 instructions perform behaviour**  **5.4 monitoring emotional consequences**  **6.1 demonstration behaviour**  **8.1 behavioural practice**  **8.7 graded tasks**  **11.2 reduce negative emotions**  **12.6 body changes (13)** | **3** |
|  |  |  |  |  |  |
| Haugmark 2021 (control) | Control - TAU and education | **Education (1)** | **Knowledge (1)** | **0** | **1** |
|  |  |  |  |  |  |
| Hutting 2015 (Intervention) | Intervention 1 - mixed psychological (e.g action planning, problem solving, goal setting, psychoeducation); work capacity and workload | **Education**  **Training**  **Enablement (3)** | **Knowledge**  **Skills**  **Beliefs capabilities**  **Goals**  **Environmental context resources**  **Emotion**  **Behavioural regulation (7)** | - 1. **goal setting behaviour**   2. **problem solving**   **1.4 action planning**  **9.1 credible source**  **11.2 reduce negative emotions (5)** | **2** |
|  |  |  |  |  |  |
| Hutting 2015 (Control) | Control TAU | **0** | **0** | **0** | **NA** |
|  |  |  |  |  |  |
| Jensen 1995 (Intervention) | Intervention 1 CBT Plus education, relaxation, passive therapy, health behaviour coaching | **Education**  **Persuasion**  **Training**  **Enablement (4)** | **Knowledge**  **Skills**  **Beliefs capabilities**  **Goals**  **Memory, attention, decision making**  **Environmental context resources**  **Social influence**  **Emotion**  **Behavioural regulation (9)** | **1.2 problem solving**  **1.3 goal setting outcome**  **1.8 behavioural contract**  **3.1 social support unspecified**  **4.1 instructions on how to perform a behaviour**  **6.1 demonstration of behaviour**  **9.1 credible source**  **12.4 Distancing**  **12.6 body changes (9)** | **2** |
|  |  |  |  |  |  |
| Jensen 1995 (Comparator) | Comparator 1 - education, relaxation, passive therapy, health behaviour coaching | **Education**  **Training**  **Enablement (3)** | **Knowledge**  **Skills**  **Goals**  **Environmental context resources (4)** | **1.3 goal setting outcome**  **4.1 instructions perform behaviour**  **6.1 demonstration behaviour**  **9.1 credible source**  **12.6 body changes**  **(5)** | **2** |
|  |  |  |  |  |  |
| Jensen 1997 Intervention 1 | Intervention 1 CBT plus exercise | **Education**  **Training**  **Enablement (3)** | **Knowledge**  **Skills**  **Beliefs capabilities**  **Goals**  **Emotion (5)** | **1.2 problem solving**  **1.3 goal setting outcome**  **3.1 social support unspecified**  **4.1 instructions perform behaviour**  **6.1 demonstration behaviour**  **8.1 behaviour practice**  **9.1 credible source**  **12.6 body changes**  **13.2 framing/reframing**  **(9)** | **2** |
|  |  |  |  |  |  |
| Jensen 1997 Intervention 2 added psychologist led group sessions) | Intervention 2 (additional psychological input) CBT plus exercise and additional psychological component (including cognitive restructuring) | **Education**  **Training**  **Enablement (3)** | **Knowledge**  **Skills**  **Beliefs capabilities**  **Goals**  **Emotion (5)** | **1.2 problem solving**  **1.3 goal setting outcome**  **3.1 social support unspecified**  **4.1 instructions perform behaviour**  **6.1 demonstration behaviour**  **8.1 behavioural practice**  **9.1 credible source**  **12.6 body changes**  **13.2 framing/reframing (9)** | **2** |
|  |  |  |  |  |  |
| Jensen 2001 (Intervention 1 CBT) | Intervention 1 - CBT | **Education**  **Training**  **Enablement (3)** | **Knowledge**  **Skills**  **Goals**  **Memory, attention, decision making (4)** | **1.2 problem solving**  **1.3 goal setting outcome**  **3.1 social support unspecified**  **4.1 instructions perform behaviour**  **12.4 distraction**  **12.6 body changes**  **(6)** | **3** |
|  |  |  |  |  |  |
| Jensen 2001 (Intervention 2 CBT and PT) | Intervention 2 - combined BOPT and CBT: CBT and Behaviour Orientated Physical Therapy (eg goal setting), plus ergonomics | **Education**  **Training**  **Enablement (3)** | **Knowledge**  **Skills**  **Goals**  **Memory, attention, decision making**  **Environmental context resources**  **(5)** | **1.2 problem solving**  **1.3 goal setting outcome**  **3.1 social support unspecified**  **4.1 instructions perform behaviour**  **6.1 demonstration behaviour**  **8.1 behaviour practice**  **8.7 graded tasks**  **12.4 distraction**  **12.6 body changes (9)** | **2** |
|  |  |  |  |  |  |
| Jensen 2001 (Comparator BOPT) | Comparator 1 - (Behaviour Orientated Physical Therapy) exercise, ergonomics | **Education**  **Training (2)** | **Knowledge**  **Skills**  **Goals**  **Environmental context resources**  **(4)** | **1.3 goal setting outcome**  **4.1 instructions perform behaviour**  **6.1 demonstration behaviour**  **8.1 behaviour practice**  **8.7 graded tasks**  **12.6 body changes**  **(6)** | **2** |
|  |  |  |  |  |  |
| Jensen 2001 (Control) | Control - TAU | **0** | **0** | **0** | **NA** |
|  |  |  |  |  |  |
| Johansson 1998 (Intervention) | intervention 1 cognitive behavioural programme - mixed psychological (eg, counselling, cognitive techniques, psychoeducation) , plus medication management; occupational training | **Education**  **Persuasion**  **Incentivisation**  **Training**  **Enablement (5)** | **Knowledge**  **Skills**  **Beliefs capabilities**  **Reinforcement**  **Goals**  **Memory, attention, decision making**  **Social influence**  **Emotion**  **Behaviour regulation (9)** | - 1. **goal setting behaviour**   **2.3 self monitoring of behaviour**  **3.1 social support unspecified**  **3.3 social support emotional**  **4.1 instructions perform behaviour**  **6.1 demonstration of behaviour**  **8.1 behaviour practice/rehearsal**  **8.7 graded tasks**  **10.4. social reward**  **11.2 reduce negative emotions**  **12.4 distraction**  **12.6 body changes**  **15.4 self talk (13)** | **2** |
|  |  |  |  |  |  |
| Johansson 1998 (Control) | Control: wait list | **0** | **0** | **0** | **NA** |
|  |  |  |  |  |  |
| Kappa 2006 (Intervention) | Intervention 1 -cog behavioural stress management - mixed psychological (eg, (rational emotive psychotherapy), psychoeducation, applied relaxation) plus exercise, ergonomics | **Education**  **Persuasion**  **Incentivisation**  **Training**  **Environmental restructuring**  **Enablement (6)** | **Knowledge**  **Skills**  **Beliefs consequences**  **Reinforcement**  **Environmental context resources**  **Emotion**  **Behavioural regulation (7)** | **2.3 self monitoring behaviour**  **2.6 biofeedback**  **3.1 social support unspecified**  **4.1 instructions perform behaviour**  **5.1 information health consequences**  **6.1 demonstration behaviour**  **8.1 behaviour practice**  **8.7 graded tasks**  **9.1 credible source**  **11.2 reduce negative emotions**  **12.1 restructure physical environment**  **12.6 body changes**  **(12)** | **3** |
|  |  |  |  |  |  |
| Kappa 2006 (Comparator) | Comparator 1 -Medical treatment | **Training**  **Enablement (2)** | **Skills**  **Environmental context resources**  **(2)** | **4.1 instructions perform behaviour**  **6.1 demonstration behaviour**  **8.1 behaviour practice**  **8.7 graded tasks**  **12.6 body changes**  **(5)** | **2** |
|  |  |  |  |  |  |
| Kool 2004 (Intervention1 FCT) | Intervention 1 Function-Centred Treatment [FCT] - counselling, plus ergonomics, exercise, medical treatment | **Education**  **Persuasion**  **Training**  **Enablement (4)** | **Knowledge**  **Skills**  **Beliefs capabilities**  **Beliefs consequences**  **Goals (5)** | - 1. **goal setting outcome**   **2.6 biofeedback**  **4.1 instructions perform behaviour**  **5.1 information health consequences**  **6.1 demonstation behaviour**  **6.2 social comparison**  **8.1 behaviour practice**  **12.6 body changes**  **13.2 Framing/reframing (9)** | **2** |
|  |  |  |  |  |  |
| Kool 2004 (Intervention 2 PCT) | Intervention 2 - Pain-Centred Treatment [PCT] - relaxation (progressive muscle relaxation), exercise, passive therapy, medical treatment | **Education**  **Training**  **Enablement (3)** | **Knowledge**  **Skills**  **Environmental context resources**  **Emotion (4)** | **4.1 instructions perform behaviour**  **6.1 demonstration behaviour**  **8.1 behaviour practice**  **8.7 graded tasks**  **11.2 reduce negative emotion**  **12.6 body changes**  **(6)** | **2** |
|  |  |  |  |  |  |
| Lambeek 2010 (Intervention) | Intervention 1 graded activity based on CBT and psychoeducation plus ergonomics | **Education**  **Persuasion**  **Incentivisation**  **Training**  **Environmental restructuring**  **Enablement (6)** | **Knowledge**  **Skills**  **Beliefs capabilities**  **Beliefs consequences**  **Reinforcement**  **Goals**  **Environmental context resources**  **Behavioural regulation (8)** | - 1. **problem solving**   2. **goal setting outcome**   3. **action planning**   **1.7 review outcome goals**  **2.1 monitor behaviour by others without feedback**  **2.5 monitor outcome of behaviour**  **3.1 social support unspecified**  **3. 2 social support practical**  **4.1 instructions perform behaviour**  **5.1 information health consequences**  **8.1 behaviour practice**  **8.7 graded tasks**  **10.10 Reward (outcome)**  **12.1 restructure physical environment**  **12.6 body changes**  **13.2 framing/reframing**  **(16)** | **3** |
|  |  |  |  |  |  |
| Lambeek 2010 (Control) | Control TAU | **Enablement (1)** | **0** | **0** | **NA** |
|  |  |  |  |  |  |
| Lemstra 2002 (intervention) | Intervention 1 - self-management-lifestyle - mixed psychological eg, psychoeducation, problem solving) plus education, exercise | **Education**  **Persuasion**  **Incentivisation**  **Training**  **Enablement (5)** | **Knowledge**  **Skills**  **Beliefs consequences**  **Reinforcement**  **Goals**  **Environmental context resources**  **Social influence**  **Emotion**  **Behavioural regulation (9)** | **1.2 problem solving**  **1.3 goal setting outcome**  **1.4 action planning**  **3.1 social support unspecified**  **3.2 social support practical**  **4.1 instructions perform behaviour**  **6.1 demonstration behaviour**  **8.1 behaviour practice**  **8.2 behaviour substitution**  **9.1 credible source**  **10.1 material incentive (behaviour)**  **10.2 material reward (behaviour)**  **11.2 reduce negative emotions**  **12.2 restructure social environment**  **12.6 body changes (15)** | **3** |
|  |  |  |  |  |  |
| Lemstra 2002 (Control) | Control - waitlist TAU |  | **0** | **0** | **NA** |
|  |  |  |  |  |  |
| Lemstra 2005 (Intervention) | Intervention 1 - self-management-lifestyle - mixed psychological eg, psychoeducation, problem solving) plus education, exercise | **Education**  **Persuasion**  **Incentivisation**  **Training**  **Enablement (5)** | **Knowledge**  **Skills**  **Beliefs consequences**  **Reinforcement**  **Goals**  **Social influences**  **Emotion**  **Behavioural regulation (8)** | **1.2 problem solving**  **1.3 goal setting outcome**  **1.4 action planning**  **3.1 social support unspecified**  **4.1 instructions perform behaviour**  **6.1 demonstration behaviour**  **8.1 behaviour practice**  **8.7 graded tasks**  **9.1 credible source**  **10.1 material incentive behaviour**  **10.2 material reward behaviour**  **12.6 body changes** | **3** |
|  |  |  |  |  |  |
| Lemstra 2005 (Control) | Control - waitlist TAU |  | **0** | **9.1 credible source** | **NA** |
|  |  |  |  |  |  |
| Lindell 2008 (Intervention) | Intervention 1 = cognitive behavioural rehabilitation Mixed psychological (eg, CBT, graded activity) plus medical treatment, education; ergonomics | **Education**  **Training**  **Enablement (3)** | **Knowledge**  **Skills**  **Goals**  **Emotion (4)** | **1.4 action planning**  **3.1 social support unspecified**  **4.1 instructions perform behaviour**  **8.1 behaviour practice**  **8.7 graded tasks**  **9.1 credible source**  **11.1 pharmacological support**  **11.2 reduce negative emotions**  **12.6 body changes**  **(9)** | **3** |
|  |  |  |  |  |  |
| Lindell 2008 (control) | Control- TAU - could include medical treatment | **Enablement (1)** | **0** | **0** | **NA** |
|  |  |  |  |  |  |
| Lindh 1997 (Intervention) | Intervention 1 Multidisciplinary Rehabilitation - Mixed psychological (eg, counselling (CBT based and family counselling, goal setting) plus exercise, education (ergonomic) | **Education**  **Persuasion**  **Training**  **Enablement (4)** | **Knowledge**  **Skills**  **Goals**  **Social influence**  **Emotion (5)** | **1.2 problem solving**  **1.3 goal setting outcome**  **1.7 review outcome goals**  **2.5 monitor outcomes of behaviour**  **3.1 social support unspecified**  **3.2 social support practical**  **3.3 social support emotional**  **4.1 instructions perform behaviour**  **6.1 demonstration behaviour**  **8.1 behaviour practice**  **9.1 credible source**  **11.2 reduce negative emotions**  **12.6 body changes**  **13.2 framing/reframing**  **(14)** | **3** |
|  |  |  |  |  |  |
| Lindh 1997 (Control) | Control - Sick list control | **0** | **0** | **0** | **NA** |
|  |  |  |  |  |  |
| Linton 1997 (Intervention 1) | Intervention 1 patient-based support group - psychoeducation (pain management) | **Education**  **Enablement (2)** | **Knowledge**  **Environmental context resources**  **Social influences (3)** | **1.2 problem solving**  **3.1 social support unspecified (2)** | **2** |
|  |  |  |  |  |  |
| Linton 1997 (Intervention 2 -Professional Support) | Intervention 2 - professional support group- CBT plus education | **Education**  **Training**  **Enablement (3)** | **Knowledge**  **Skills**  **Social influence**  **Emotion (4)** | **1.2 problem solving**  **3.1 social support unspecified**  **8.1 behavioural practice**  **11.2 reduce negative emotions (4)** | **3** |
|  |  |  |  |  |  |
| Linton 1997 (Control) | Control TAU - could include medical treatment | **Enablement (1)** | **0** | **0** | **NA** |
|  |  |  |  |  |  |
| Magnussen 2007 (Intervention) | Intervention 1 = Mixed Psychological (eg. counselling, Motivational interviewing, psychoeducation) plus education (ergonomic); vocational counselling | **Education**  **Persuasion**  **Enablement (3)** | **Knowledge**  **Beliefs consequences**  **Memory, attention, decision making**  **Social influence**  **Emotion (5)** | **1.2 problem solving**  **3,1 social support unspecified**  **9.1 credible source**  **11.2 reduce negative emotions**  **(4)** | **2** |
|  |  |  |  |  |  |
| Magnussen 2007 (Control) | Control group | **0** | **0** | **0** | **NA** |
|  |  |  |  |  |  |
| Marhold 2001 (Intervention1 LTSL)) | Intervention 1 (LTSL) CBT plus exercise; occupational training | **Education**  **Training**  **Enablement (3)** | **Knowledge**  **Skills**  **Goals**  **Memory, attention, decision making**  **Emotion (5)** | - 1. **goal setting behaviour**   2. **problem solving**   **4.1 instructions perform behaviour**  **6.1 demonstration behaviour**  **8.1 Behaviour practice**  **8.7 graded tasks**  **9.1 credible source**  **11.2 reduce negative emotions**  **12.6 body changes**  **13.2 framing/reframing (10)** | **3** |
|  |  |  |  |  |  |
| Marhold 2001 (Intervention 2 STSL) | Intervention 2 (STSL) CBT plus exercise | **Education**  **Training**  **Enablement (3)** | **Knowledge**  **Skills**  **Goals**  **Memory, attention, dec making**  **Emotion (5)** | **1.1 goal setting behaviour**  **1.2 problem solving**  **4.1 instructions perform behaviour**  **6.1 demonstration behaviour**  **8.1 behaviour practice**  **8.7 graded tasks**  **9.1 credible source**  **11.2 reduce negative emotions**  **12.6 body changes**  **13.2 framing/reframing**  **(10)** | **3** |
|  |  |  |  |  |  |
| Marhold 2001 (Control 1 LTSL) | Control 1 (LTSL) TAU could include medical treatment, psychologist | **Enablement (1)** | **0** | **0** | **NA** |
|  |  |  |  |  |  |
| Marhold 2001 (Control 2 STSL) | Control 2 (STSL) TAU could include medical treatment, psychologist | **Enablement (1)** | **0** | **0** | **NA** |
|  |  |  |  |  |  |
| McKnight 2010 (Intervention 1 self-management) | Intervention 1 self-management (coping and SE), Mixed psychological (eg problem solving, psychoeducation). | **Education**  **Persuasion**  **Training**  **Enablement (4)** | **Knowledge**  **Skills**  **Beliefs capability**  **Memory, attention, decision making**  **Environmental context resources (5)** | **1.2 problem solving**  **3,2 social support practical**  **5.1 information health consequences**  **13.2 framing/reframing**  **(4)** | **2** |
|  |  |  |  |  |  |
| McKnight 2010 (Intervention 2 combined) | Intervention2 (Combined) self-management (coping and SE), Mixed psychological (eg problem solving, psychoeducation), plus exercise | **Education**  **Persuasion**  **Training**  **Enablement (4)** | **Knowledge**  **Skills**  **Beliefs capabilities**  **Memory, attention, decision making**  **Environmental context resources**  **Social influence (6)** | - 1. **problem solving**   **2.2 feedback on behaviour**  **2.4 self monitor outcome of behaviour**  **3.2 social support practical**  **4.1 instructions perform behaviour**  **5.1 information health consequences**  **6.1 demonstration of behaviour**  **8.1 behaviour practice**  **8.7 graded tasks**  **12.6 body changes**  **13.2 framing/reframing (11)** | **2** |
|  |  |  |  |  |  |
| McKnight 2010 (Comparator ) | Comparator (Strength Training) - Exercise | **Persuasion**  **Training (2)** | **Skills**  **Beliefs capabilities**  **Environmental context resources**  **(3)** | **2.2 feedback on behaviour**  **2.4 self-monitor outcome of behaviour**  **4.1 instructions perform behaviour**  **6.1 demonstration of behaviour**  **8.1 behaviour practice**  **8.7 graded tasks**  **9.1 credible source**  **12.6 body changes**  **(8)** | **2** |
|  |  |  |  |  |  |
| Meyer 2005 (intervention) | Intervention 1 - operant behavioural therapy approach (eg, coping strategies and SE), exercise, education (ergonomics), workplace visit, sports activities | **Education**  **Persuasion**  **Training**  **Enablement (4)** | **Knowledge**  **Skills**  **Beliefs capabilities (3)** | **1.7 review outcome goals**  **4.1 instructions perform behaviour**  **6.1 demonstration behaviour**  **8.1 behaviour practice**  **8.7 graded tasks**  **9.1 credible source**  **11.2 reduce negative emotions**  **12.6 body changes (8)** | **2** |
|  |  |  |  |  |  |
| Meyer 2005 (control) | Comparator 1: Exercise, education, medical treatment | **Education**  **Training**  **Enablement (3)** | **Knowledge**  **Skills**  **(2)** | **4.1 instructions perform behaviour**  **6.1 demonstration behaviour**  **8.1 behaviour practice**  **8.7 graded tasks**  **9.1 credible source**  **11.1 pharmacological support**  **11.2 reduce negative emotions**  **12.6 body changes**  **(8)** | **2** |
|  |  |  |  |  |  |
| Miller 2020 (intervention) | Intervention 1 -self-management based on cognitive behavioural principles: Mixed psychological (eg, psychoeducation, coping) plus exercise | **Education**  **Persuasion**  **Training**  **Enablement (4)** | **Knowledge**  **Skills**  **Beliefs capabilities**  **Optimism**  **Beliefs consequences**  **Goals**  **ECR**  **Social influence**  **Emotion**  **Behavioural regulation (10)** | **1.1 goal setting behaviour**  **1.2 problem solving**  **1.3 goal setting outcomes**  **1.4 action planning**  **1.5 review behaviour goals**  **2.2 feedback on behaviour**  **2.3 self monitoring behaviour**  **2.4 self-monitoring outcomes of behaviour**  **3.1 social support unspecified**  **3.2 social support practical**  **4.1 instructions perform behaviour**  **5.1 information health consequences**  **8.1 behaviour practice**  **8.7 graded tasks**  **9.1 credible source**  **11.2 reduce negative emotions**  **12.6 body changes**  **13.2 framing/reframing**  **15.3 focus on past success**  **15.4 self-talk (20)** | **3** |
|  |  |  |  |  |  |
| Miller 2020 (control) | Control TAU - could include medical treatment, referral | **Enablement (1)** | **0** | **0** | **NA** |
|  |  |  |  |  |  |
| Mitchell 1994 (Intervention) | Intervention 1 Behavioural and cognitive therapy, plus exercise | **Education**  **Persuasion**  **Training**  **Enablement (4)** | **Knowledge**  **Skills**  **Beliefs capabilities**  **Goals**  **Environmental context resources**  **Social influence**  **Emotion (7)** | **1.3 goal setting outcome**  **2.6 biofeedback**  **3.1 social support unspecified**  **4.1 instructions perform behaviour**  **6.1 demonstration behaviour**  **8.1 behaviour practice**  **8.7 graded tasks**  **12.6 body changes (8)** | **2** |
|  |  |  |  |  |  |
| Mitchell 1994 (Control) | Control - TAU -medication, exercise, physio | **Enablement (1)** |  | **9.1 credible source (1)** | **1** |
|  |  |  |  |  |  |
| Pach 2022 (Intervention) | Intervention 1 - Mixed psychological (eg, autogenic training, mindfulness, guided imagery) | **Education**  **Persuasion**  **Training**  **Enablement (4)** | **Knowledge**  **Skills**  **Goals**  **Memory, attention, dec making**  **ECRs**  **Emotion**  **Behavioural regulation (7)** | **1.4 action planning**  **2.3 self-monitoring behaviour**  **4.1 instructions perform behaviour**  **7.1 prompts/cues**  **8.1 behaviour practice**  **9.1 credible source**  **11.2 reduce negative emotions**  **12.6 body changes**  **(8)** | **3** |
|  |  |  |  |  |  |
| Pach 2022 (Control) | Control Waitlist control -TAU | **Enablement (1)** | **Behavioural regulation (1)** | **2.3 self-monitoring of behaviour**  **(1)** | **3** |
|  |  |  |  |  |  |
| Pato 2010 (Intervention 1) | Intervention 1 CBT and Infiltration | **Education**  **Persuasion**  **Training**  **Modelling**  **(4)** | **Knowledge**  **Skills**  **Beliefs capabilities**  **Emotion (4)** | **3.1 social support unspecified**  **4.1 instructions perform behaviour**  **6.1 demonstration of behaviour**  **8.1 Behaviour practice**  **8.7 graded tasks**  **9.1 credible source**  **11.2 reduce negative emotions**  **12.6 body changes (8)** | **3** |
|  |  |  |  |  |  |
| Pato 2010 (Intervention 2) | Intervention 2 CBT and Physiotherapy | **Education**  **Persuasion**  **Training**  **Modelling (4)** | **Knowledge**  **Skills**  **Beliefs capabilities**  **Emotion (4)** | **2.3 self-monitoring of behaviour**  **3.1 social support unspecified**  **4.1 instructions perform behaviour**  **6.1 demonstration behaviour**  **8.1 Behavioural practice**  **8.7 Graded tasks**  **9.1 credible source**  **11.2 reduce negative emotions**  **12.6 body changes (9)** | **2** |
|  |  |  |  |  |  |
| Pato 2010 (Intervention 3) | Intervention 3 CBT and Medication | **Education**  **Persuasion**  **Training**  **Modelling (4)** | **Knowledge**  **Skills**  **Beliefs capabilities**  **Emotion (4)** | **3.1 social support unspecified**  **4.1 instructions perform behaviour**  **6.1 demonstration behaviour**  **8.1 behaviour practice**  **8.7 graded tasks**  **9.1 credible source**  **11.2 reduce negative emotions**  **12.6 body changes (8)** | **2** |
|  |  |  |  |  |  |
| Pato 2010 (Comparator 1) | Comparator 1 Infiltration only | **Enablement (1)** | **0** | **12.6 body changes (1)** | **1** |
|  |  |  |  |  |  |
| Pato 2010 (Comparator 2) | Comparator 2 Physiotherapy only | **Education**  **Training**  **Enablement (3)** | **Knowledge**  **Skills**  **(2)** | **2.3 Self-monitoring of behaviour**  **4.1 Instructions perform behaviour**  **8.1 Behaviour practice**  **12.6 Body changes (4)** | **2** |
|  |  |  |  |  |  |
| Pato 2010 (Comparator 3) | Comparator 3 Medication only | **Enablement (1)** | **0** | **0** | **NA** |
|  |  |  |  |  |  |
| Reme 2016 (Intervention 1 BI) | Intervention 1 Brief Cognitive Intervention (eg, coping skills -NOT CBT) plus education | **Education**  **Persuasion**  **Enablement (3)** | **Knowledge**  **Optimism**  **Beliefs consequences (3)** | **1.2 Problem solving**  **1.3 goal setting outcome**  **2.2 feedback on behaviour**  **2.7 feedback on outcomes of behaviour**  **3.1 social support unspecified**  **5.1 information health consequences**  **9.1 credible source**  **13.2 Framing/reframing**  **(8)** | **3** |
|  |  |  |  |  |  |
| Reme 2016 (Intervention 2 - BI and CBT) | Intervention 2 (BI and CBT) - Mixed psychological (eg, CBT) plus education | **Education**  **Persuasion**  **Training**  **Enablement (4)** | **Knowledge**  **Skills**  **Beliefs capabilities**  **Optimism**  **Beliefs consequences**  **Goals**  **Memory, attention, decision making**  **Social influence**  **Emotion**  **Behavioural regulation (10)** | **1.1 Goal setting behaviour**  **1.2 problem solving**  **1.3 goal setting outcome**  **1.4 action planning**  **1.5 review behaviour goals**  **1.7 review outcome goals**  **1.9 commitment**  **2.2 feedback on behaviour**  **2.7 feedback on outcomes of behaviour**  **3.1 social support unspecified**  **3.3 social support emotional**  **4.1 instructions perform behaviour**  **5.1 information health consequences**  **5.4 monitoring emotional consequences**  **8.1 behavioural practice**  **9.1 credible source**  **11.2 reduce negative emotions**  **12.4 distraction**  **13.2 Framing/reframing**  **(19)** | **3** |
|  |  |  |  |  |  |
| Reme 2016 (Intervention 3 - BI and GROUP CBT) | Intervention 3 (BI and Group CBT) - Mixed psychological (eg, CBT) plus education | **Education**  **Persuasion**  **Training**  **Enablement (4)** | **Knowledge**  **Skills**  **Beliefs capabilities**  **Optimism**  **Beliefs consequences**  **Goals**  **Memory, attention, decision making**  **Social influence**  **Emotion**  **(9)** | **1.2 problem solving**  **1.3 goal setting outcome**  **2.2 feedback on behaviour**  **2.7 feedback on outcomes of behaviour**  **3.1 social support unspecified**  **4.1 instructions perform behaviour**  **5.1 information health consequences**  **7.7 exposure**  **8.1 Behaviour practice**  **9.1 credible source**  **11.2 reduce negative emotions**  **13.2 framing/reframing**  **(11)** | **2** |
|  |  |  |  |  |  |
| Reme 2016 (Intervention 4 - BI and PE) | Intervention 4 (BI and PE) - cognitive intervention (eg, coping skills -NOT CBT) plus Exercise, education. | **Education**  **Persuasion**  **Training**  **Enablement (4)** | **Knowledge**  **Skills**  **Beliefs capabilities**  **Optimism**  **Beliefs consequences**  **Goals (6)** | - 1. **problem solving**   **1.3 goal setting outcome**  **2.2 feedback on behaviour**  **2.7 feedback on outcomes of behaviour**  **3.1 social support unspecified**  **4.1 instructions perform behaviour**  **5.1 information health consequences**  **6.1 demonstration behaviour**  **8.1 Behaviour practice**  **9.1 credible source**  **12.6 body changes**  **13.2 Framing/reframing**  **(12)** | **2** |
|  |  |  |  |  |  |
| Reme 2016 (Intervention 5 - BI and Seal Oil) | Intervention 5 (BI plus seal oil) - Brief Cognitive Intervention (BI) - (eg, coping skills -NOT CBT) plus education, seal oil. - | **Education**  **Persuasion**  **Enablement (3)** | **Knowledge**  **Optimism**  **Beliefs consequences**  **Environmental context resources (4)** | **1.2 problem solving**  **1.3 goal setting outcome**  **2.2 feedback on behaviour**  **2.7 feedback on outcomes of behaviour**  **3.1 social support unspecified**  **5.1 information health consequences**  **9.1 credible source**  **13.2 Framing/reframing**  **(8)** | **3** |
|  |  |  |  |  |  |
| Reme 2016 (Intervention 6 - BI and Soy Oil) | Intervention 6: (BI plus soy oil) - Brief Cognitive Intervention (BI) - (eg, coping skills -NOT CBT) plus education, soy oil. - | **Education**  **Persuasion**  **Enablement (3)** | **Knowledge**  **Optimism**  **Beliefs consequences**  **Environmental context resources**  **(4)** | **1.2 problem solving**  **1.3 goal setting outcome**  **2.2 feedback on behaviour**  **2.7 feedback on outcomes of behaviour**  **3.1 social support unspecified**  **5.1 information health consequences**  **9.1 credible source**  **13.2 framing/reframing**  **(8)** | **3** |
|  |  |  |  |  |  |
| Rolving 2015 (Intervention) | Intervention 1: CBT plus education, medical treatment, return to work | **Education**  **Training (2)** | **Knowledge**  **Skills**  **Beliefs consequences**  **Reinforcement**  **Goals**  **Memory, attention, decision making**  **Social influence**  **Emotion (8)** | **1.2 problem solving**  **1.3 goal setting outcome**  **1.7 review outcome goals**  **2.2 feedback on behaviour**  **2.3 self-monitoring of behaviour**  **3.1 social support unspecified**  **3.3 social support emotional**  **4.1 instructions performing behaviour**  **6.1 demonstration behaviour**  **8.1 Behaviour practice**  **8.7 graded tasks**  **9.1 credible source**  **11.2 reduce negative emotions**  **12.6 body changes**  **13.2 framing/reframing**  **15.3 focus on past success (16)** | **2** |
|  |  |  |  |  |  |
| Rolving 2015 Comparator | Comparator 1 TAU -education, medical treatment | **Education**  **Training (2)** | **Knowledge**  **Skills (2)** | **4.1 instructions perform behaviour**  **6.1 demonstration of behaviour**  **8.1 behaviour practice**  **9.1 credible source**  **12.6 body changes (5)** | **3** |
|  |  |  |  |  |  |
| Rolving 2022 (Intervention) | Intervention 1 - CBT plus biofeedback, education, medical treatment | **Education**  **Persuasion**  **Training**  **Environmental restructuring**  **Modelling**  **Enablement (6)** | **Knowledge**  **Skills**  **Beliefs capabilities**  **Reinforcement**  **Goals**  **Memory, attention, dec making**  **ECR**  **Social influence**  **Emotion**  **Behavioural regulation (10)** | **1.1 goal setting behaviour**  **1.2 problem solving**  **1.3 goal setting outcome**  **1.4 action planning**  **2.6 biofeedback**  **3.1 social support unspecified**  **3.3 social support emotional**  **4.1 instructions perform behaviour**  **5.1 information health consequences**  **6.1 demonstration behaviour**  **7.7 exposure**  **8.1 behaviour practice**  **8.7 graded tasks**  **9.1 credible source**  **10.4 social reward**  **11.2 reduce negative emotions**  **12.6 body changes**  **13.2 framing/reframing**  **(18)** | **3** |
|  |  |  |  |  |  |
| Rolving 2022 Comparator | Comparator 1TAU - biofeedback, education, medical treatment | **Education**  **Enablement (2)** | **Knowledge (1)** | **1.4 action planning**  **2.6 Biofeedback**  **5.1 information health consequences**  **9.1 credible source (4)** | **2** |
|  |  |  |  |  |  |
| Sander 2020 (intervention) | Intervention 1 Mixed psychological (CBT based including psychoeducation), | **Education**  **Persuasion**  **Incentivisation**  **Training**  **Environmental restructuring**  **Enablement (6)** | **Knowledge**  **Skills**  **Reinforcement**  **Goals**  **Environmental context resources**  **Social influence**  **Emotion**  **Behavioural regulation (8)** | **1.1 goal setting behaviour**  **1.2 problem solving**  **1.3 goal setting outcome**  **2.2 feedback on behaviour**  **2.4 self-monitoring outcomes of behaviour**  **3.1 social support unspecified**  **4.1 instructions perform behaviour**  **5.4 monitor emotional consequences of behaviour**  **7.1 prompts/cues**  **8.1 behaviour practice/rehearsal**  **9.1 credible source**  **11.2 reduce negative emotions**  **15.3 focus on past success (13)** | **3** |
|  |  |  |  |  |  |
| Sander 2020 (Control) | Control TAU - could include medical treatment | **Enablement (1)** | **0** | **0** | **NA** |
|  |  |  |  |  |  |
| Schlicker 2020 (Intervention) | Intervention 1 Mixed psychological (CBT based including psychoeducation), plus ergonomics | **Education**  **Persuasion**  **Training**  **Environmental restructuring**  **Enablement (5)** | **Knowledge**  **Skills**  **Reinforcement**  **Goals**  **Environmental context resources**  **Social influence**  **Emotion**  **Behavioural regulation (8)** | **1.1 goal setting behaviour**  **1.2 problem solving**  **1.3 goal setting outcome**  **2.2 feedback on behaviour**  **2.4 self-monitoring outcomes of behaviour**  **3.1 social support unspecified**  **4.1 instructions perform behaviour**  **5.4 Monitoring emotional consequences**  **7.1 prompts/cues**  **8.1 behaviour practice**  **9.1 credible source**  **11.2 reduce negative emotions**  **12.1 restructure physical environment**  **13.2 Framing/reframing**  **15.3 Focus on past success (15)** | **3** |
|  |  |  |  |  |  |
| Schlicker 2020 (control) | Control TAU | **0** | **0** | **0** | **NA** |
|  |  |  |  |  |  |
| Schweikert 2006 (Intervention) | Intervention 1 Cognitive Behavioural Treatment , plus medical treatment, exercise, education | **Education**  **Persuasion**  **Training**  **Enablement (4)** | **Knowledge**  **Skills**  **Beliefs capabilities**  **Memory, attention, dec making**  **Environmental context resources**  **Social influence**  **Emotions (7)** | **1.2 problem solving**  **3.1 social support unspecified**  **4.1 instructions perform behaviour**  **5.1 information health consequences**  **6.1 demonstration behaviour**  **8.1 behaviour practice**  **9.1 credible source**  **11.2 reduce negative emotions**  **12.4 Distraction**  **12.6 body changes**  **13.2 Framing/reframing**  **15.3 Focus on past success (11)** | **2** |
|  |  |  |  |  |  |
| Schweikert 2006 (Control) | Comparator 1 -medical treatment, exercise, education | **Education**  **Training**  **Enablement (3)** | **Knowledge**  **Skills (2)** | **4.1 instructions perform behaviour**  **5.1 information health consequences**  **6.1 demonstration behaviour**  **8.1 behaviour practice**  **12.6 body changes (5)** | **3** |
|  |  |  |  |  |  |
| Skouen 2002b Intervention 1 (light multidisciplinary): | Intervention 1 (Light multidisciplinary) - psychoeducation plus exercise, workplace intervention | **Education**  **Persuasion**  **Training**  **Enablement (4)** | **Knowledge**  **Skills**  **Beliefs capabilities**  **Beliefs consequences**  **Social influence (5)** | **4.1 instructions perform behaviour**  **5.1 information health consequences**  **8.7 graded tasks**  **13.2 Framing/reframing**  **(4)** | **3** |
|  |  |  |  |  |  |
| Skouen 2002 Intervention 2 (Extensive multidisciplinary): | Intervention 2 (extensive multidisciplinary) - Cognitive behavioural treatment Mixed psychological (eg, cognitive coping, psychoeducation)) plus , exercise, and occasional workplace interventions | **Education**  **Persuasion**  **Training**  **(3)** | **Knowledge**  **Skills**  **Beliefs capabilities**  **Social influence**  **Emotion (5)** | **3.1 social support unspecified**  **4.1 instructions perform behaviour**  **6.1 demonstration behaviour**  **8.1 Behavioural practice**  **8.7 Graded tasks**  **12.6 Body changes**  **13.2 Framing/reframing (7)** | **2** |
|  |  |  |  |  |  |
| Skouen 2002 (Control) | Control: TAU -could include medical treatment, advice, referral. | **Enablement (1)** | **0** | **0** | **NA** |
|  |  |  |  |  |  |
| Skouen 2006 Intervention 1 (light multidisciplinary): | Intervention 1 (Light multidisciplinary) - psychoeducation plus exercise, workplace intervention | **Education**  **Persuasion**  **Training**  **Enablement (4)** | **Knowledge**  **Skills**  **Beliefs about capabilities**  **Beliefs consequences**  **Social influences (5)** | **4.1 instructions perform behaviour**  **5.1 information health consequences**  **8.7 Graded tasks**  **13.2 Framing/reframing (4)** | **2** |
|  |  |  |  |  |  |
| Skouen 2006 Intervention 2 (Extensive multidisciplinary): | Intervention 2 (extensive multidisciplinary) - Cognitive behavioural treatment Mixed psychological (eg, cognitive coping, psychoeducation)) plus , exercise, and occasional workplace interventions | **Education**  **Training**  **Enablement (3)** | **Knowledge**  **Skills**  **Belief capability**  **Social influence**  **Emotion (5)** | **3.1 social support unspecified**  **4.1 instructions perform behaviour**  **6.1 Demonstration behaviour**  **8.1 Behaviour practice**  **8.7 Graded tasks**  **12.6 body changes**  **13.2 Framing/reframing (7)** | **2** |
|  |  |  |  |  |  |
| Skouen 2006 (Control) | Control: TAU -could include medical treatment, advice, referral. | **Enablement (1)** | **0** | **11.1 Pharmacological support**  **112. reduce negative emotions (2)** | **1** |
|  |  |  |  |  |  |
| Smeets 2009 (Intervention 1 GAP) | Intervention 1 (GAP) - Problem Solving Training plus exercise (graded activity) | **Education**  **Persuasion**  **Training**  **Enablement (4)** | **Knowledge**  **Skills**  **Memory attention, decision making**  **Environmental context resources (4)** | **1.2 problem solving**  **3.1 social support unspecified**  **4.1 instructions perform behaviour**  **6.1 demonstration behaviour**  **8.1 Behaviour practice**  **8.7Graded tasks**  **9.1 credible source**  **(7)** | **3** |
|  |  |  |  |  |  |
| Smeets 2009 (Intervention 2 Combined ) | Intervention 2 (Combined) Problem Solving Training plus exercise | **Education**  **Persuasion**  **Training**  **Enablement (4)** | **Knowledge**  **Skills**  **Beliefs consequences**  **Reinforcement**  **Memory, attention, dec making**  **ECR (6)** | **1.2 problem solving**  **4.1 instructions perform behaviour**  **5.1 information health consequences**  **6.1 Demonstration behaviour**  **8.1 Behaviour practice**  **8.7 Graded tasks**  **9.1 credible source**  **12.6 body changes (8)** | **3** |
|  |  |  |  |  |  |
| Smeets 2009 (Comparator) | Comparator 1 APT): Exercise | **Training (1)** | **Skills**  **ECR (2)** | **4.1 instructions perform behaviour**  **6.1 demonstration behaviour**  **8.1 behaviour practice**  **12.6 body changes (4)** | **2** |
|  |  |  |  |  |  |
| Smeets 2009 (Control) | Control - Wait list | **0** | **0** | **0** | **NA** |
|  |  |  |  |  |  |
| Van Eijk-Hustings 2013 (Intervention 1MD) | Intervention 1 - Mixed psychological (eg Psychotherapy and sociotherapy) plus medical treatment | **Education**  **Training**  **Enablement (3)** | **Knowledge**  **Skills**  **Memory, attention, dec making**  **Social influences**  **Emotion (5)** | **1.2 problem solving**  **4.1 instructions perform behaviour**  **6.1 Demonstration behaviour**  **8.1 Behaviour practice**  **11.2 Reduce negative emotions**  **12. 6 body changes (6)** | **3** |
|  |  |  |  |  |  |
| Van Eijk-Hustings 2013 (Comparator AE) | Comparator 1 (aerobic exercise) = exercise | **Education**  **Training (2)** | **Knowledge**  **Skills**  **Environmental context resources**  **Behavioural regulation (4)** | **2.4 self monitoring of outcomes of behaviour**  **4.1 instructions perform behaviour**  **6.1 Demonstration of behaviour**  **8.1 Behavioural practice**  **8.7 Graded tasks**  **12.6 Body changes (6)** | **3** |
|  |  |  |  |  |  |
| Van Eijk-Hustings 2013 (control) | Control - TAI included education, could also include medical treatment or social support | **Education**  **Enablement (2)** | **Knowledge**  **Social influences (2)** | **9.1 credible source (1)** | **2** |
|  |  |  |  |  |  |
| von Korff 2005 (intervention 1) | Intervention 1 - Mixed psychological (eg action planning, problem solving) plus exercise | **Education**  **Persuasion**  **Enablement (3)** | **Knowledge**  **Skills**  **Beliefs consequences**  **Goals**  **Environmental context resources**  **Emotion**  **Behavioural regulation (7)** | **1.1 Goal setting behaviour**  **1.2 Problem solving**  **1.4 Action planning**  **3.1 social support unspecified**  **4.1 instructions perform behaviour**  **5.1 information health consequences**  **6.1 demonstration behaviour**  **9.1 credible source**  **11.2 reduce negative emotions**  **12.6 body changes**  **(10)** | **2** |
|  |  |  |  |  |  |
| von Korff 2005 (control) | Control 1 TAU -could include medical treatment | **Enablement (1)** | **0** | **0** | **NA** |
|  |  |  |  |  |  |

Supplementary file 6: Distribution of intervention functions, TDF domains and Behavioural Change Techniques

|  | **Psychological interventions  (n = 74)** | | **Comparator/ control conditions (n = 50)** | | **Overall  (n = 124)** |
| --- | --- | --- | --- | --- | --- |
|  | Frequency | % | Frequency | % | Frequency (%) |
| **Behaviour change techniques** | | | | | |
| 1.1 Goal setting (behaviour) | 11 | 15% | 0 | 0% | 11 (9%) |
| 1.2 Problem solving | 45 | 61% | 0 | 0% | 45 (36%) |
| 1.3 Goal setting (outcome) | 36 | 49% | 4 | 8% | 40 (32%) |
| 1.4 Action planning | 14 | 19% | 1 | 2% | 15 (12%) |
| 1.5 Review behaviour goals | 2 | 3% | 0 | 0% | 2 (2%) |
| 1.6 Discrepancy between current behaviour and goal | 0 | 0% | 0 | 0% | 0% |
| 1.7 Review outcome goals | 5 | 7% | 0 | 0% | 5 (4%) |
| 1.8 Behavioural contract | 4 | 5% | 0 | 0% | 4 (3%) |
| 1.9 Commitment | 1 | 1% | 0 | 0% | 1.(0.8%) |
| 2.1 Monitoring of behaviour by others without feedback | 3 | 4% | 0 | 0% | 3 (2%) |
| 2.2 Feedback on behaviour | 18 | 24% | 2 | 4% | 20 (16%) |
| 2.3 Self-monitoring of behaviour | 14 | 19% | 2 | 4% | 16 (13%) |
| 2.4 Self-monitoring of outcome(s) of behaviour | 6 | 8% | 2 | 4% | 8 (6%) |
| 2.5 Monitoring of outcomes of behaviour without feedback | 3 | 4% | 1 | 2% | 4 (3%) |
| 2.6 Biofeedback | 12 | 16% | 2 | 4% | 14 (11%) |
| 2.7 Feedback on outcome(s) of behaviour | 11 | 15% | 0 | 0% | 11 (9%) |
| 3.1 Social support (unspecified) | 55 | 74% | 3 | 6% | 58 (47%) |
| 3.2 Social support (practical) | 11 | 15% | 1 | 2% | 12 (10% |
| 3.3 Social support (emotional) | 6 | 8% | 0 | 0% | 6 (4%) |
| 4.1 Instruction on how to perform a behaviour | 64 | 86% | 20 | 40% | 84 (68%) |
| 4.2 Information about antecedents | 0 | 0% | 0 | 0% | 0% |
| 4.3 Re-attribution | 0 | 0% | 0 | 0% | 0% |
| 4.4 Behavioural experiments | 1 | 1% | 0 | 0% | 1 (0.8%) |
| 5.1 Information about health consequences | 25 | 34% | 3 | 6% | 28 (23%) |
| 5.2 Saliance of consequences | 0 | 0% | 0 | 0% | 0% |
| 5.3 Information about social and environmental consequences | 0 | 0% | 0 | 0% | 0% |
| 5.4 Monitoring of emotional consequences | 5 | 7% | 0 | 0% | 5 (4%) |
| 5.5 Anticipated regret | 0 | 0% | 0 | 0% | 0% |
| 5.6 Information about emotional consequences | 0 | 0% | 0 | 0% | 0% |
| 6.1 Demonstration of the behaviour | 47 | 64% | 13 | 26% | 60 (48%) |
| 6.2 Social comparison | 3 | 4% | 0 | 0% | 3 (2%) |
| 6.3 Information about others' approval | 0 | 0% | 0 | 0% | 0% |
| 7.1 Prompts/cues | 5 | 7% | 0 | 0% | 5 (4%) |
| 7.2 Cue signalling reward | 0 | 0% | 0 | 0% | 0% |
| 7.3 Reduce prompts/ cues | 0 | 0% | 0 | 0% | 0% |
| 7.4 Remove access to the award | 0 | 0% | 0 | 0% | 0% |
| 7.5 Remove aversive stimulus | 0 | 0% | 0 | 0% | 0% |
| 7.6 Satiation | 0 | 0% | 0 | 0% | 0% |
| 7.7 Exposure | 3 | 4% | 0 | 0% | 3 (2%) |
| 7.8 Associative learning | 0 | 0% | 0 | 0% | 0% |
| 8.1 Behavioural practice/ rehearsal | 59 | 80% | 15 | 30% | 74 (60%) |
| 8.2 Behaviour substitution | 1 | 1% | 0 | 0% | 1 (0.8%) |
| 8.3 Habit formation | 0 | 0% | 0 | 0% | 0% |
| 8.4 Habit reversal | 0 | 0% | 0 | 0% | 0% |
| 8.5 Overcorrection | 0 | 0% | 0 | 0% | 0% |
| 8.6 Generalisation of target behaviour | 1 | 1% | 0 | 0% | 1 (0.8%) |
| 8.7 Graded tasks | 32 | 43% | 8 | 16% | 40 (32%) |
| 9.1 Credible source | 44 | 59% | 11 | 22% | 55 (44%) |
| 9.2 Pros and cons | 0 | 0% | 0 | 0% | 0% |
| 9.3 Comparative imagining of future outcomes | 0 | 0% | 0 | 0% | 0% |
| 10.1 Material incentive (behaviour) | 3 | 4% | 0 | 0% | 3 (2%) |
| 10.2 Material reward (behaviour) | 2 | 3% | 0 | 0% | 2 (1%) |
| 10.3 Non-specific reward | 1 | 1% | 0 | 0% | 1 (0.8%) |
| 10.4 Social reward | 4 | 5% | 0 | 0% | 4 (3%) |
| 10.5 Social incentive | 0 | 0% | 0 | 0% | 0% |
| 10.6 Non-specific incentive | 1 | 1% | 0 | 0% | 1 (0.8%) |
| 10.7 Self-incentive | 1 | 1% | 0 | 0% | 1 (0.8%) |
| 10.8 Incentive (outcome) | 0 | 0% | 0 | 0% | 0% |
| 10.9 Self-reward | 2 | 3% | 0 | 0% | 2 (1%) |
| 10.10 Reward (outcome) | 1 | 1% | 0 | 0% | 1 (0.8%) |
| 10.11 Future Punishment | 0 | 0% | 0 | 0% | 0% |
| 11.1 Pharmacological support | 3 | 4% | 1 | 2% | 4 (3%) |
| 11.2 Reduce negative emotions | 45 | 61% | 4 | 8% | 49 (40%) |
| 11.3 Conserving mental resources | 0 | 0% | 0 | 0% | 0% |
| 11.4 Paradoxical instructions | 0 | 0% | 0 | 0% | 0% |
| 12.1 Restructuring the physical environment | 5 | 7% | 0 | 0% | 5 (4%) |
| 12.2 Restructuring the social environment | 1 | 1% | 0 | 0% | 1 (0.8%) |
| 12.3 Avoidance/ reducing exposure to cues for the behaviour | 0 | 0% | 0 | 0% | 0% |
| 12.4 Distraction | 6 | 8% | 0 | 0% | 6 (5%) |
| 12.5 Adding objects to the environment | 2 | 3% | 0 | 0% | 2 (2%) |
| 12.6 Body changes | 54 | 73% | 19 | 38% | 73 (59%) |
| 13.1 Identification of self as a role model | 0 | 0% | 0 | 0% | 0% |
| 13.2 Framing/ reframing | 33 | 45% | 0 | 0% | 33 (27%) |
| 13.3 Incompatible beliefs | 0 | 0% | 0 | 0% | 0% |
| 13.4 Valued self-identify | 0 | 0% | 0 | 0% | 0% |
| 13.5 Identity associated with changed behaviour | 1 | 1% | 0 | 0% | 1 (0.8%) |
| 14.1 Behaviour cost | 0 | 0% | 0 | 0% | 0% |
| 14.2 Punishment | 0 | 0% | 0 | 0% | 0% |
| 14.3 Remove reward | 0 | 0% | 0 | 0% | 0% |
| 14.4 Reward approximation | 0 | 0% | 0 | 0% | 0% |
| 14.5 Reward completion | 0 | 0% | 0 | 0% | 0% |
| 14.6 Situation-specific reward | 0 | 0% | 0 | 0% | 0% |
| 14.7 Reward incompatible behaviour | 0 | 0% | 0 | 0% | 0% |
| 14.8 Reward alternative behaviour | 0 | 0% | 0 | 0% | 0% |
| 14.9 Reduce reward frequency | 0 | 0% | 0 | 0% | 0% |
| 14.10 Remove punishment | 0 | 0% | 0 | 0% | 0% |
| 15.1 Verbal persuasion about capability | 0 | 0% | 0 | 0% | 0% |
| 15.2 Mental rehearsal of success performance | 0 | 0% | 0 | 0% | 0% |
| 15.3 Focus on past success | 6 | 8% | 0 | 0% | 6 (5%) |
| 15.4 Self-talk | 2 | 3% | 0 | 0% | 2 (1%) |
| 16.1 Imaginary punishment | 0 | 0% | 0 | 0% | 0% |
| 16.2 Imaginary reward | 0 | 0% | 0 | 0% | 0% |
| 16.3 Vicarious consequences | 0 | 0% | 0 | 0% | 0% |
| **Theoretical domains** | | | | | |
| Knowledge | 74 | 100% | 18 | 36% | 92 (74%) |
| Skills | 68 | 92% | 17 | 34% | 85 (69%) |
| Social/professional role and identity | 0 | 0% | 0 | 0% | 0 (0%) |
| Beliefs about capabilities | 43 | 58% | 1 | 2% | 44 (35%) |
| Optimism | 8 | 11% | 0 | 0% | 8 (6%) |
| Beliefs about consequences | 24 | 32% | 1 | 2% | 25 (20%) |
| Reinforcement | 14 | 19% | 0 | 0% | 14 (11%) |
| Intentions | 0 | 0% | 0 | 0% | 0 (0%) |
| Goals | 40 | 54% | 4 | 8% | 44 (35%) |
| Memory, attention and decision processes | 23 | 31% | 0 | 0% | 23 (19%) |
| Environmental context and resources | 35 | 47% | 6 | 12% | 41 (33%) |
| Social influences | 43 | 58% | 4 | 8% | 47 (38%) |
| Emotion | 46 | 62% | 2 | 4% | 48 (39%) |
| Behavioural regulation | 25 | 34% | 3 | 6% | 28 (23%) |
| **Intervention functions** | | | | | |
| Education | 74 | 100% | 18 | 36% | 92 (74%) |
| Persuasion | 51 | 69% | 4 | 8% | 55 (44%) |
| Incentivisation | 9 | 12% | 0 | 0% | 9 (7%) |
| Coercion | 0 | 0% | 0 | 0% | 0 (0%) |
| Training | 65 | 88% | 17 | 34% | 82 (66%) |
| Restriction | 0 | 0% | 0 | 0% | 0 (0%) |
| Environmental restructuring | 11 | 15% | 0 | 0% | 11 (9%) |
| Modelling | 3 | 4% | 0 | 0% | 3 (2%) |
| Enablement | 66 | 89% | 28 | 56% | 94 (76%) |

Supplementary file 7: Description and mapping of the 5 common intervention components

| ***Common component within interventions and areas mapped** | **Definition in the context of the interventions** | **Intervention function** | **Theoretical domain** | **BCT** | **Examples of intervention content** |
| --- | --- | --- | --- | --- | --- |
| **Knowledge and education** | Education related to the condition, psychological pain management strategies, exercise techniques and to a lesser degree education involved ergonomics advice. At times the interventions focussed on education linked to broader life issues e.g. hobbies, not just pain management. | Education | Knowledge | Not mapped | Psychoeducation eg chair and desk arrangement at work  Pain management coping skills eg linked to CBT  Information about ergonomics  Reconceptualising pain and life problems  Back school education  BCT skills for use with pain  Vocational rehabilitation  Teaching strategies to manage fatigue  Education around pacing and improving motivation  Coping and acceptance  Classes on anatomy and physiology  Learning how to write a job resume  The physiology of the condition and how to avoid muscle dysfunction  Functioning training and activity planning  Considering impact of cognition and fear on pain and disability  Education about the importance of fitness for managing pain  Stress management education  The relationship between the mind and body  Health behaviour and coping strategies  Cognitive restricting and reducing cognitive errors  Increasing knowledge about the body  Education about the impact of activity on pain  Optional workplace visit to give additional instructions or training if necessary  Teaching coping skills related to the workplace e.g. heavy lifting  Practical advice on how the condition can be improved  Psychoeducation on how to adapt the workplace to employee needs |
| **Skills and training** | Training tended to involve exercise for pain or condition management and mobility, and psychological skills for pain/condition management to a lesser degree. There was comparatively little work-focussed training. Sometimes the training was tailored to individual needs, and could be delivered in a particular setting, with instructions to perform the behaviour at home. In some cases, the training involved the fine tuning of developed skills. | Training | Skills | 4.1. Instructions on how to perform a behaviour  6.1 Demonstration of the behaviour  8.1. Behavioural practice/rehearsal  12.6 Body changes | Exercises -muscular strength  Relaxation training  Body awareness training  Coping skills, problem solving and goal setting training (eg as part of CBT)  Weight training  Aerobic fitness training  Ergonomics training eg lifting at different heights  Taught how to stretch at home and advised about daily walks  In OT, training focussed on a a simulated work situation and work intensification, such as lifting, sitting and standing  Individual exercises for endurance and co-ordination  Instructions on performing personal and vocational activities  Depression preventing training  Self-management training  Graded activity training eg in relation to going for a walk  Ergonomics training  Guided physical training  Autogenic training |
| **Reducing negative emotions and stress** | Includes reconceptualising pain, mood, stress, anxiety, depression, fear, highlighting feelings and thoughts looking at link between mood and outcomes, as well as addressing anger and fear of pain and activity. | Persuasion  Enablement | Emotion | 11.2. Reducing negative emotions | Addressing emotions  Defining depression-related goals  Sessions looked at the connection between pleasant activities and mood  Patients were encouraged to overcome fear that might contribute to the avoidance of physical activity  Applied relaxation for stress management  Focus on pain-reducing and pain enhancing emotions  Attending to emotional experience. Discussion of anger and contributors to angry feelings  Stress and stress management strategies  CBT for anxiety and depression  Emotional support  Autogenic training for stress and anxiety  Relaxation and stress reduction  Fear avoidance management  Addressing fears about pain  Activity to replace negative or stressful feelings |
| **Promoting beliefs about capabilities** | Includes confidence building, operant conditioning approaches, self-efficacy, empowerment, activity to encourage people to take responsibility, encouragement. | Education  Persuasion  Enablement | Beliefs about capabilities | Not mapped | Encouraging patients to deal with life stress  Classes build confidence in ability to manage health and stay active  Helping patients to take responsibility for their condition  Activities to increase self-confidence and self-responsibility  Instilling confidence in ability to undertake activities of daily life  Promote self-management competencies and self-efficacy expectations  Encourage patients to self-manage their condition  Enhance internal locus of control  Sessions aim to increase new skills and learn pain control techniques  Patients to reconceptualise their ability to control pain  Helping patients to explore their physical boundaries and overcome barriers to movement  Improving self-efficacy  Activity to increase self-efficacy and self-confidence  Promoting adaptive strategies and reducing passive strategies  Teaching control of pain through controlling physical reactions to stress, and pain management techniques  Encouraged to think about self-confidence and adopting positive attitudes |
| **Social support** | Needs to be a person involved and discussed in a supportive way, such as working with intervention deliverer for support, being in group context for intervention, support group, working with family, employer, friends on discussion of behaviour and intervention plan. | Enablement | Social influences | 3.1 Social support (unspecified) | Group discussion and support within intervention sessions  Health supervisor help patients take an active part in their own lives  Sessions with professionals, such as psychologists within the interventions  Participants within the group interventions provide encouragement and support to others to continue to engage with the intervention  Receiving guidance from a counselling partner within the intervention  Opportunity to have family involvement in the rehab planning  Social exchange of experiences within intervention group  Behaviour change in everyday life assessed with spouses and work supervisor  Involvement of family or friends to support the therapy |

*At least 2 of the intervention function, TDF domains and BCTs mapped against each other

Supplementary file 8: Panel graph displaying publication bias funnel plots for (A) work status; (B) return to work; (C) sick leave; and (D) work capacity

**
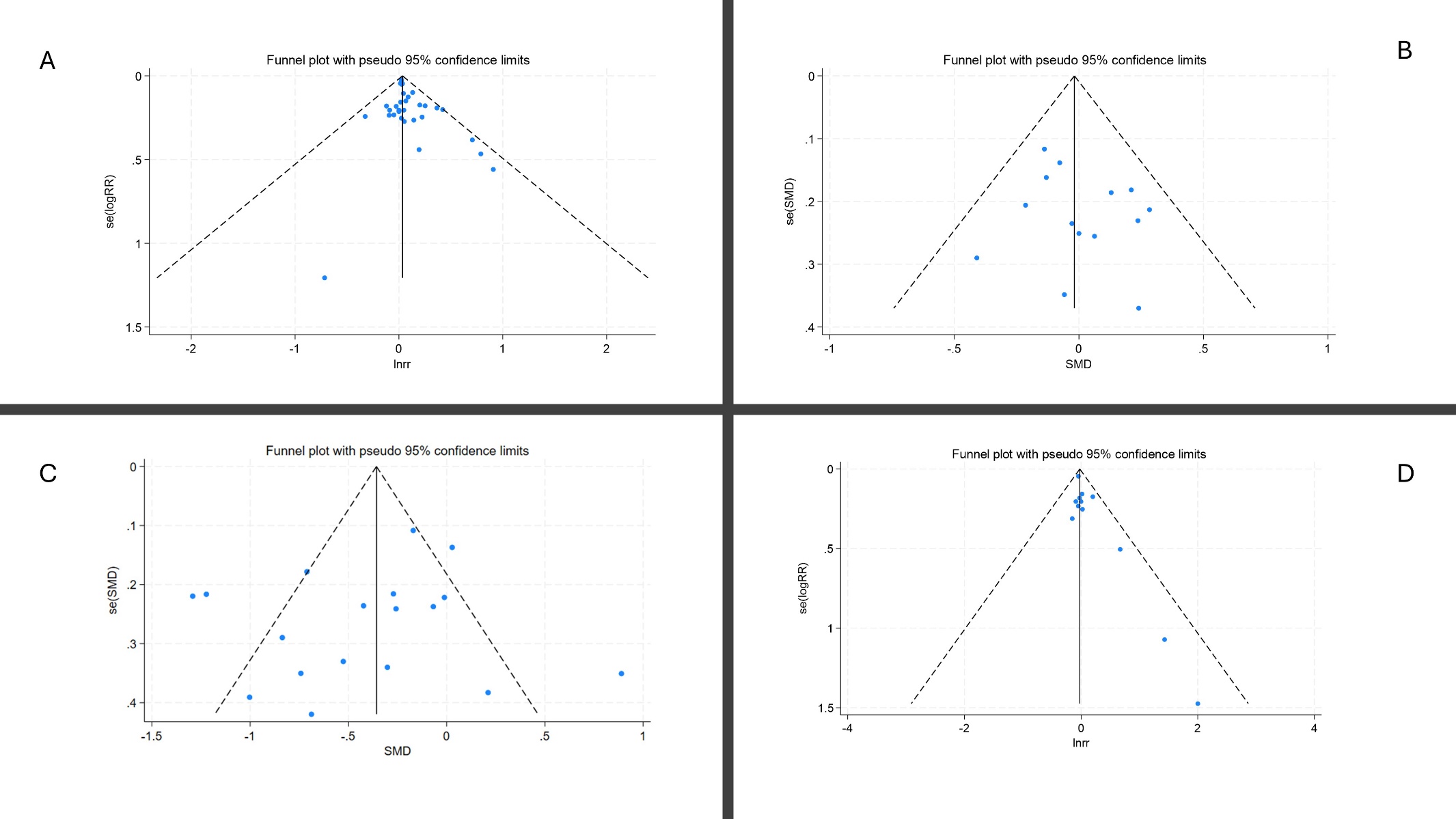
**

| Supplementary file 9: GRADE summary of findings for all work outcomes | | | | | | |
| --- | --- | --- | --- | --- | --- | --- |
| **Psychological interventions compared to non-psychological interventions or controls for people with chronic pain** | | | | | | |
| **Patient or population:** people with chronic pain  **Setting:**  **Intervention:** Psychological interventions  **Comparison:** non-psychological interventions or controls | | | | | | |
| Outcomes | **Anticipated absolute effects^*^** (95% CI) | | Relative effect  (95% CI) | № of participants  (studies) | Certainty of the evidence  (GRADE) | Comments |
|  | **Risk with non-psychological interventions or controls** | **Risk with Psychological interventions** |  |  |  |  |
| Work status   follow-up: mean 40 months | 614 per 1,000 | **632 per 1,000**  (620 to 651) | **RR 1.03**  (1.01 to 1.06) | 4087  (24 RCTs) | ⨁◯◯◯  Very low^a,b,c^ | Psychological interventions may have a small effect on work status but the evidence is very uncertain. |
| Return to work  follow-up: mean 24 months | 541 per 1,000 | **519 per 1,000**  (492 to 568) | **RR 0.96**  (0.91 to 1.05) | 1470  (9 RCTs) | ⨁◯◯◯  Very low^a,b,c,d^ | The evidence is very uncertain about the effect of psychological interventions on return to work. |
| Sick leave  follow-up: mean 12.5 months | - | SMD **0.41 SD lower**  (0.64 lower to 0.18 lower) | - | 1607  (14 RCTs) | ⨁◯◯◯  Very low^a,b,e^ | Psychological interventions may reduce sick leave but the evidence is very uncertain. |
| Work capacity  follow-up: mean 12.4 months | - | SMD **0.02 SD lower**  (0.12 lower to 0.08 higher) | - | 1658  (13 RCTs) | ⨁◯◯◯  Very low^a,b,d^ | The evidence is very uncertain about the effect of psychological interventions on work capacity. |
| ***The risk in the intervention group** (and its 95% confidence interval) is based on the assumed risk in the comparison group and the **relative effect** of the intervention (and its 95% CI).    **CI:** confidence interval; **RR:** risk ratio; **SMD:** standardised mean difference | | | | | | |
| **GRADE Working Group grades of evidence**  **High certainty:** we are very confident that the true effect lies close to that of the estimate of the effect.  **Moderate certainty:** we are moderately confident in the effect estimate: the true effect is likely to be close to the estimate of the effect, but there is a possibility that it is substantially different.  **Low certainty:** our confidence in the effect estimate is limited: the true effect may be substantially different from the estimate of the effect.  **Very low certainty:** we have very little confidence in the effect estimate: the true effect is likely to be substantially different from the estimate of effect. | | | | | | |

**Explanations**

a. Downgraded by one level for risk of bias: the majority of studies were assessed as some concerns or high risk of bias

b. Downgraded by one level for indirectness: population characteristics not well reported (especially in terms of work characteristics), interventions included a mixture of work focus and pain/clinical focus, and outcome definitions and measurement methods varied

c. Downgraded by one level for evidence of publication bias: funnel plot asymmetry and Egger test

d. Downgraded by one level for imprecision: 95% confidence interval is wide and overlaps null effect

e. Downgraded by one level for inconsistency: evidence of substantial heterogeneity (I2 >50%)

Supplementary file10: Effectiveness of interventions containing psychological components in relation to work status at different follow-up time points

Supplementary file 11: Meta-analysis of intervention coding components for all outcomes

| **Intervention Component** | **Component estimate (RR / SMD) (95% CI), p-value** | | | |
| --- | --- | --- | --- | --- |
|  | **Work status (RR)** | **Return to work (RR)** | **Sick leave (SMD)** | **Work capacity (SMD)** |
| Intervention functions | | | | |
| Total number of included Ifs | RR 1.03 (0.99 to 1.08), p=0.17 | 1.01 (0.88 to 1.16) p=0.90 | 0.002 (-0.24 to 0.24), p=0.984 | -0.08 (-0.16 to 0.00), p=0.05 |
| Education | NA^a^ | NA^a^ | NA^a^ | NA^a^ |
| Persuasion | RR 1.00 (0.93 to 1.07), p=0.92 | 0.98 (0.74 to 1.31), p=0.91 | 0.27 (-0.26 to 0.79), p=0.30 | -0.23 (-0.53 to 0.08), p=0.13 |
| Incentivisation | RR 1.07 (0.91 to 1.26), p=0.38 | 5.23 (0.76 to 36.16), p=0.09* | 0.42 (-0.42 to 1.26), p=0.30 | -0.24 (-0.47 to -0.01), p=0.045 |
| Coercion | NA^b^ | NA^b^ | NA^b^ | NA^b^ |
| Training | RR 1.02 (0.94 to 1.10), p=0.62 | 0.97 (0.70 to 1.33), p=0.82 | 0.12 (-1.13 to 1.37), p=0.84 | -0.20 (-0.51 to 0.11), p=0.18 |
| Restriction | NA^b^ | NA^b^ | NA^b^ | NA^b^ |
| Environmental restructuring | RR 1.06 (0.91 to 1.25), p=0.43 | NA^b^ | -0.18 (-1.00 to 0.63), p=0.64 | -0.18 (-0.41 to 0.48), p=0.11 |
| Modelling | RR 0.98 (0.75 to 1.28), p=0.91 | NA^b^ | -0.55 (-1.42 to 0.32), p=0.20 | NA^a^ |
| Enablement | RR 1.03 (0.97 to 1.09), p=0.40 | 1.03 (0.80 to 1.33), p=0.78 | -0.33 (-1.08 to 0.42), p=0.37 | -0.05 (-0.46 to 0.35), p=0.78 |
| Theoretical domains | | | | |
| Total number of included TDFs | 1.00 (0.98 to 1.03), p=0.82 | 1.00 (0.86 to 1.18), p=0.95 | 0.11 (-0.05 to 0.26), p=0.18 | -0.06 (-0.12 to 0.01), p=0.09 |
| Knowledge | NA^a^ | NA^a^ | NA^a^ | NA^a^ |
| Skills | 2.12 (0.18 to 24.90), p=0.54 | 0.50 (0.16 to 1.54), p=0.20 | 0.12 (-1.13 to 1.37), p=0.84 | -0.32 (-0.80 to 0.16), p=0.17 |
| Social/Professional role and identity | NA^b^ | NA^b^ | NA^b^ | NA^b^ |
| Beliefs about capabilities | 0.99 (0.94 to 1.05), p=0.81 | 1.06 (0.86 to 1.30), p=0.56 | 0.05 (-0.49 to 0.60), p=0.84 | -0.08 (-0.32 to 0.16), p=0.47 |
| Optimism | NA^b^ | NA^b^ | NA^b^ | NA^b^ |
| Beliefs about consequences | 1.01 (0.94 to 1.08), p=0.75 | 1.19 (0.89 to 1.58), p=0.21 | 0.11 (-0.53 to 0.75), p=0.72 | -0.07 (-0.32 to 0.19), p=0.57 |
| Reinforcement | 1.08 (0.94 to 1.23), p=0.27 | 5.23 (0.76 to 36.16), p=0.09* | 0.44 (-0.26 to 1.13), p=0.20 | -0.16 (-0.40 to 0.08), p=0.17 |
| Intentions | NA^b^ | NA^b^ | NA^b^ | NA^b^ |
| Goals | 1.01 (0.95 to 1.07), p=0.80 | 0.89 (0.70 to 1.13), p=0.30 | 0.15 (-0.39 to 0.69), p=0.56 | -0.03 (-0.31 to 0.26), p=0.85 |
| Memory, attention and decision processes | 0.98 (0.92 to 1.04), p=0.43 | 1.11 (0.61 to 2.01), p=0.72 | 0.20 (-0.34 to 0.74), p=0.44 | 0.32 (-0.16 to 0.80), p=0.17 |
| Environmental context and resources | 1.00 (0.94 to 1.06), p=0.91 | NA^b^ | 0.05 (-0.50 to 0.60), p=0.86 | -0.19 (-0.44 to 0.06), p=0.12 |
| Social influences | 1.01 (0.96 to 1.08), p=0.63 | 0.96 (0.67 to 1.38), p=0.81 | -0.16 (-0.70 to 0.37), p=0.53 | -0.04 (-0.28 to 0.20), p=0.73 |
| Emotion | 1.00 (0.93 to 1.07), p=0.92 | 0.93 (0.73 to 1.19), p=0.55 | 0.11 (-0.46 to 0.68), p=0.69 | -0.03 (-0.40 to 0.33), p=0.84 |
| Behavioural regulation | 1.01 (0.94 to 1.08), p=0.82 | 1.02 (0.74 to 1.43), p=0.87 | 0.37 (-0.24 to 0.99), p=0.22 | -0.21 (-0.49 to 0.07), p=0.12 |
| Behaviour change techniques | | | | |
| Total number of included BCTs | 1.01 (0.99 to 1.02), p=0.28 | 0.99 (0.97 to 1.01), p=0.30 | 0.04 (-0.05 to 0.12), p=0.36 | -0.03 (-0.57 to 0.00), p=0.05 |
| 1.1 Goal setting (behaviour) | 0.99 (0.92 to 1.07), p=0.78 | NA^b^ | 0.42 (-0.33 to 1.16), p=0.25 | -0.01 (-0.24 to 0.22), p=0.92 |
| 1.2 Problem solving | 1.01 (0.95 to 1.07), p=0.85 | 0.94 (0.78 to 1.14), p=0.51 | -0.22 (-0.76 to 0.32), p=0.40 | -0.04 (-0.29 to 0.20), p=0.71 |
| 1.3 Goal setting (outcome) | 1.02 (0.94 to 1.09), p=0.66 | 0.90 (0.73 to 1.11), p=0.29 | -0.25 (-0.82 to 0.31), p=0.36 | -0.11 (-0.34 to 0.12), p=0.31 |
| 1.4 Action planning | 1.01 (0.94 to 1.08), p=0.87 | 1.10 (0.77 to 1.57), p=0.56 | 0.09 (-0.70 to 0.89), p=0.81 | 0.05 (-0.19 to 0.30), p=0.65 |
| 1.5 Review behaviour goals | 2.40 (0.77 to 7.51), p=0.13 | NA^b^ | NA^b^ | NA^b^ |
| 1.6 Discrepency between current behaviour and goal | NA^b^ | NA^b^ | NA^b^ | NA^b^ |
| 1.7 Review outcome goals | 1.03 (0.85 to 1.25), p=0.73 | 0.91 (0.75 to 1.10), p=0.30 | 0.04 (-0.77 to 0.86), p=0.91 | -0.04 (-0.81 to 0.73), p=0.91 |
| 1.8 Behavioural contract | 1.25 (0.86 to 1.80). p=0.23 | NA^b^ | NA^b^ | -0.41 (-1.05 to 0.24), p=0.20 |
| 1.9 Commitment | NA^b^ | NA^b^ | NA^b^ | NA^b^ |
| 2.1 Monitoring of behaviour by others without feedback | 1.00 (0.92 to 1.07), p=0.90 | NA^b^ | -0.32 (-1.41 to 0.77), p=0.54 | NA^b^ |
| 2.2 Feedback on behaviour | 1.00 (0.93 to 1.08), p=0.90 | 0.88 (0.44 to 1.77), p=0.69 | 0.43 (-0.68 to 1.34), p=0.43 | -0.13 (-0.36 to 0.99), p=0.24 |
| 2.3 Self-monitoring of behaviour | 1.05 (0.86 to 1.28), p=0.62 | 0.88 (0.44 to 1.77), p=0.69 | 0.77 (0.20 to 1.34), p=0.01 | -0.20 (-0.43 to 0.04), p=0.10 |
| 2.4 Self-monitoring of outcome(s) of behaviour | 2.24 (1.08 to 4.65), p=0.03 | NA^b^ | NA^b^ | -0.13 (-0.35 to 0.10), p=0.25 |
| 2.5 Monitoring of outcomes of behaviour without feedback | 1.14 (0.95 to 1.36), p=0.15 | 0.92 (0.76 to 1.11), p=0.36 | -0.32 (-1.41 to 0.77), p=0.54 | -0.41 (-1.05 to 0.24), p=0.20 |
| 2.6 Biofeedback | 0.99 (0.91 to 1.08), p=0.81 | NA^b^ | -0.37 (-1.09 to 0.35), p=0.29 | -0.21 (-0.67 to 0.25), p=0.34 |
| 2.7 Feedback on outcome(s) of behaviour | 1.02 (0.58 to 1.78), p=0.95 | NA^b^ | NA^b^ | NA^b^ |
| 3.1 Social support (unspecified) | 0.99 (0.92 to 1.07), p=0.77 | 0.87 (0.63 to 1.20), p=0.34 | 0.24 (-0.30 to 0.77), p=0.36 | 0.02 (-0.32 to 0.35), p=0.91 |
| 3.2 Social support (practical) | 1.11 (0.97 to 1.26), p=0.13 | 0.93 (0.77 to 1.12), p=0.39 | -0.50 (-1.18 to 0.19), p=0.14 | -0.41 (-1.05 to 0.24), p=0.20 |
| 3.3 Social support (emotional) | 1.00 (0.84 to 1.18), p=0.94 | 0.91 (0.75 to 1.10), p=0.30 | 0.60 (-0.08 to 1.28), p=0.08 | NA |
| 4.1 Instruction on how to perform a behaviour | 0.70 (0.47 to 1.05), p=0.08 | 0.50 (0.16 to 1.54), p=0.20 | -0.15 (-1.28 to 0.98), p=0.78 | -0.25 (-0.58 to 0.08), p=0.13 |
| 4.2 Information about antecedents | NA^b^ | NA^b^ | NA^b^ | NA^b^ |
| 4.3 Re-attribribution | NA^b^ | NA^b^ | NA^b^ | NA^b^ |
| 4.4 Behavioural experiments | NA^b^ | NA^b^ | -0.12 (-1.37 to 1.13), p=0.84 | NA^b^ |
| 5.1 Information about health consequences | 1.01 (0.94 to 1.09), p=0.71 | 1.16 (0.84 to 1.60), p=0.34 | 0.10 (-0.49 to 0.69), p=0.72 | -0.20 (-0.55 to 0.16), p=0.25 |
| 5.2 Saliance of consequences | NA^b^ | NA^b^ | NA^b^ | NA^b^ |
| 5.3 Information about social and environmental consequences | NA^b^ | NA^b^ | NA^b^ | NA^b^ |
| 5.4 Monitoring of emotional consequences | 2.13 (0.82 to 5.51), p=0.12 | NA^b^ | NA^b^ | -0.13 (-0.35 to 0.10), p=0.25 |
| 5.5 Anticipated regret | NA^b^ | NA^b^ | NA^b^ | NA^b^ |
| 5.6 Information about emotional consequences | NA^b^ | NA^b^ | NA^b^ | NA^b^ |
| 6.1 Demonstration of the behaviour | 0.91 (0.79 to 1.06), p=0.21 | 0.92 (0.74 to 1.13), p=0.37 | -0.12 (-0.81 to 0.56), p=0.71 | -0.16 (-0.41 to 0.09), p=0.19 |
| 6.2 Social comparison | NA^b^ | NA^b^ | NA^b^ | NA^b^ |
| 6.3 Information about others' approval | NA^b^ | NA^b^ | NA^b^ | NA^b^ |
| 7.1 Prompts/cues | 1.34 (0.95 to 1.88), p=0.10 | NA^b^ | 0.47 (-0.56 to 1.51), p=0.35 | -0.13 (-0.36 to 0.10), p=0.24 |
| 7.2 Cue signalling reward | NA^b^ | NA^b^ | NA^b^ | NA^b^ |
| 7.3 Reduce prompts/ cues | NA^b^ | NA^b^ | NA^b^ | NA^b^ |
| 7.4 Remove access to the award | NA^b^ | NA^b^ | NA^b^ | NA^b^ |
| 7.5 Remove aversive stimulus | NA^b^ | NA^b^ | NA^b^ | NA^b^ |
| 7.6 Satiation | NA^b^ | NA^b^ | NA^b^ | NA^b^ |
| 7.7 Exposure | 0.86 (0.59 to 1.24) p=0.40 | NA^b^ | -0.12 (-1.37 to 1.13), p=0.84 | NA^b^ |
| 7.8 Associative learning | NA^b^ | NA^b^ | NA^b^ | NA^b^ |
| 8.1 Behavioural practice/ rehearsal | 1.01 (0.94 to 1.08), p=0.83 | 0.91 (0.72 to 1.15), p=0.39 | NA^b^ | -0.21 (-0.48 to 0.05), p=0.11 |
| 8.2 Behaviour substitution | 1.01 (0.81 to 1.25), p=0.93 | 7.58 (0.28 to 202.49), p=0.20 | NA^b^ | NA^b^ |
| 8.3 Habit formation | NA^b^ | NA^b^ | NA^b^ | NA^b^ |
| 8.4 Habit reversal | NA^b^ | NA^b^ | NA^b^ | NA^b^ |
| 8.5 Overcorrection | NA^b^ | NA^b^ | NA^b^ | NA^b^ |
| 8.6 Generalisation of target behaviour | NA^b^ | NA^b^ | 0.65 (-0.63 to 1.93), p=0.30 | NA^b^ |
| 8.7 Graded tasks | 1.01 (0.93 to 1.09), p=0.86 | 1.08 (0.88 to 1.33), p=0.43 | 0.22 (-0.32 to 0.75), p=0.40 | -0.09 (-0.34 to 0.15), p=0.43 |
| 9.1 Credible source | 1.01 (0.96 to 1.08), p=0.65 | 0.91 (0.72 to 1.17), p=0.43 | 0.23 (-0.32 to 0.77), p=0.39 | 0.21 (-0.12 to 0.54), p=0.20 |
| 9.2 Pros and cons | NA^b^ | NA^b^ | NA^b^ | NA^b^ |
| 9.3 Comparative imagaining of future outcomes | NA^b^ | NA^b^ | NA^b^ | NA^b^ |
| 10.1 Material incentive (behaviour) | 1.12 (0.95 to 1.33), p=0.18 | 5.23 (0.76 to 36.16), p=0.09* | NA^b^ | -0.41 (-1.05 to 0.24), p=0.20 |
| 10.2 Material reward (behaviour) | 1.09 (0.90 to 1.32), p=0.37 | 5.23 (0.76 to 36.16), p=0.09* | NA^b^ | NA^b^ |
| 10.3 Non-specific reward | 1.25 (0.86 to 1.80), p=0.23 | NA^b^ | NA^b^ | -0.41 (-1.05 to 0.24), p=0.20 |
| 10.4 Social reward | 1.03 (0.80 to 1.34), p=0.80 | NA^b^ | 1.36 (0.25 to 2.48), p=0.02 | -0.41 (-1.05 to 0.24), p=0.20 |
| 10.5 Social incentive | NA^b^ | NA^b^ | NA^b^ | NA^b^ |
| 10.6 Non-specific incentive | 1.25 (0.86 to 1.80), p=0.23 | NA^b^ | NA^b^ | -0.41 (-1.05 to 0.24), p=0.20 |
| 10.7 Self-incentive | 1.25 (0.86 to 1.80), p=0.23 | NA^b^ | NA^b^ | -0.41 (-1.05 to 0.24), p=0.20 |
| 10.8 Incentive (outcome) | NA^b^ | NA^b^ | NA^b^ | NA^b^ |
| 10.9 Self-reward | 1.12 (0.85 to 1.49), p=0.41 | NA^b^ | -0.45 (-1.63 to 0.73), p=0.43 | -0.41 (-1.05 to 0.24), p=0.20 |
| 10.10 Reward (outcome) | NA^b^ | NA^b^ | -0.32 (-1.41 to 0.77), p=0.54 | NA^b^ |
| 10.11 Future Punishment | NA^b^ | NA^b^ | NA^b^ | NA^b^ |
| 11.1 Pharmacological support | 0.98 (0.71 to 1.35), p=0.91 | 1.04 (0.73 to 1.49), p=0.81 | NA^b^ | 0.25 (-0.16 to 0.66), p=0.21 |
| 11.2 Reduce negative emotions | 1.00 (0.94 to 1.06), p=0.97 | 0.90 (0.71 to 1.15), p=0.36 | 0.01 (-0.64 to 0.66), p=0.98 | NA^b^ |
| 11.3 Conserving mental resources | NA^b^ | NA^b^ | NA^b^ | NA^b^ |
| 11.4 Paradoxical instructions | NA^b^ | NA^b^ | NA^b^ | NA^b^ |
| 12.1 Restructuring the physical environment | 1.11 (0.82 to 1.48), p=0.49 | NA^b^ | -0.32 (-1.41 to 0.77), p=0.54 | 0.00 (-0.35 to 0.36), p=0.98 |
| 12.2 Restructuring the social environment | 1.01 (0.81 to 1.25), p=0.93 | 7.58 (0.28 to 202.49), p=0.20 | NA^b^ | NA^b^ |
| 12.3 Avoidance/ reducing exposure to cues for the behaviour | NA^b^ | NA^b^ | NA^b^ | NA^b^ |
| 12.4 Distraction | 1.04 (0.82 to 1.32), p=0.75 | NA^b^ | 0.73 (-0.02 to 1.49), p=0.06 | NA^b^ |
| 12.5 Adding objects to the environment | 1.25 (0.86 to 1.80), p=0.23 | NA^b^ | NA^b^ | -0.41 (-1.05 to 0.24), p=0.20 |
| 12.6 Body changes | 0.88 (0.67 to 1.16), p=0.36 | 0.83 (0.61 to 1.13), p=0.21 | -0.04 (-0.91 to 0.84), p=0.93 | -0.05 (-0.28 to 0.19), p=0.68 |
| 13.1 Identification of self as a role model | NA^b^ | NA^b^ | NA^b^ | NA^b^ |
| 13.2 Framing/ reframing | 1.07 (0.96 to 1.19), p=0.21 | 0.94 (0.73 to 1.20), p=0.56 | -0.14 (-0.68 to 0.40), p=0.59 | 0.27 (-0.25 to 0.78), p=0.28 |
| 13.3 Incompatible beliefs | NA^b^ | NA^b^ | NA^b^ | NA^b^ |
| 13.4 Valued self-identify | NA^b^ | NA^b^ | NA^b^ | NA^b^ |
| 13.5 Identity associated with changed behaviour | 1.48 (0.98 to 2.23), p=0.06 | NA^b^ | 0.15 (-0.98 to 1.28), p=0.78 | NA^b^ |
| 14.1 Behaviour cost | NA^b^ | NA^b^ | NA^b^ | NA^b^ |
| 14.2 Punishment | NA^b^ | NA^b^ | NA^b^ | NA^b^ |
| 14.3 Remove reward | NA^b^ | NA^b^ | NA^b^ | NA^b^ |
| 14.4 Reward approximation | NA^b^ | NA^b^ | NA^b^ | NA^b^ |
| 14.5 Reward completion | NA^b^ | NA^b^ | NA^b^ | NA^b^ |
| 14.6 Situation-specific reward | NA^b^ | NA^b^ | NA^b^ | NA^b^ |
| 14.7 Reward incompatable behaviour | NA^b^ | NA^b^ | NA^b^ | NA^b^ |
| 14.8 Reward alternative behaviour | NA^b^ | NA^b^ | NA^b^ | NA^b^ |
| 14.9 Reduce reward frequency | NA^b^ | NA^b^ | NA^b^ | NA^b^ |
| 14.1 Remove punishment | NA^b^ | NA^b^ | NA^b^ | NA^b^ |
| 15.1 Verbal persuasion about capability | NA^b^ | NA^b^ | NA^b^ | NA^b^ |
| 15.2 Mental rehearsal of success performance | NA^b^ | NA^b^ | NA^b^ | NA^b^ |
| 15.3 Focus on past success | 1.01 (0.67 to 1.52), p=0.98 | 0.88 (0.44 to 1.77), p=0.69 | 0.36 (-0.41 to 1.14), p=0.34 | -0.08 (-0.32 to 0.15), p=0.46 |
| 15.4 Self-talk | 2.40 (0.77 to 7.51), p=0.13 | NA^b^ | 1.36 (0.25 to 2.48), p=0.02 | NA^b^ |
| 16.1 Imaginary punishment | NA^b^ | NA^b^ | NA^b^ | NA^b^ |
| 16.2 Imaginary reward | NA^b^ | NA^b^ | NA^b^ | NA^b^ |
| 16.3 Vicarious consequences | NA^b^ | NA^b^ | NA^b^ | NA^b^ |

*model did not converge using REML, presented result using MM^; a^: Not applicable as all interventions reporting this outcome included this component^; b^: Not applicable as no interventions reporting this outcome included this component.

Supplementary file 12: Risk of bias in studies included in work status analysis


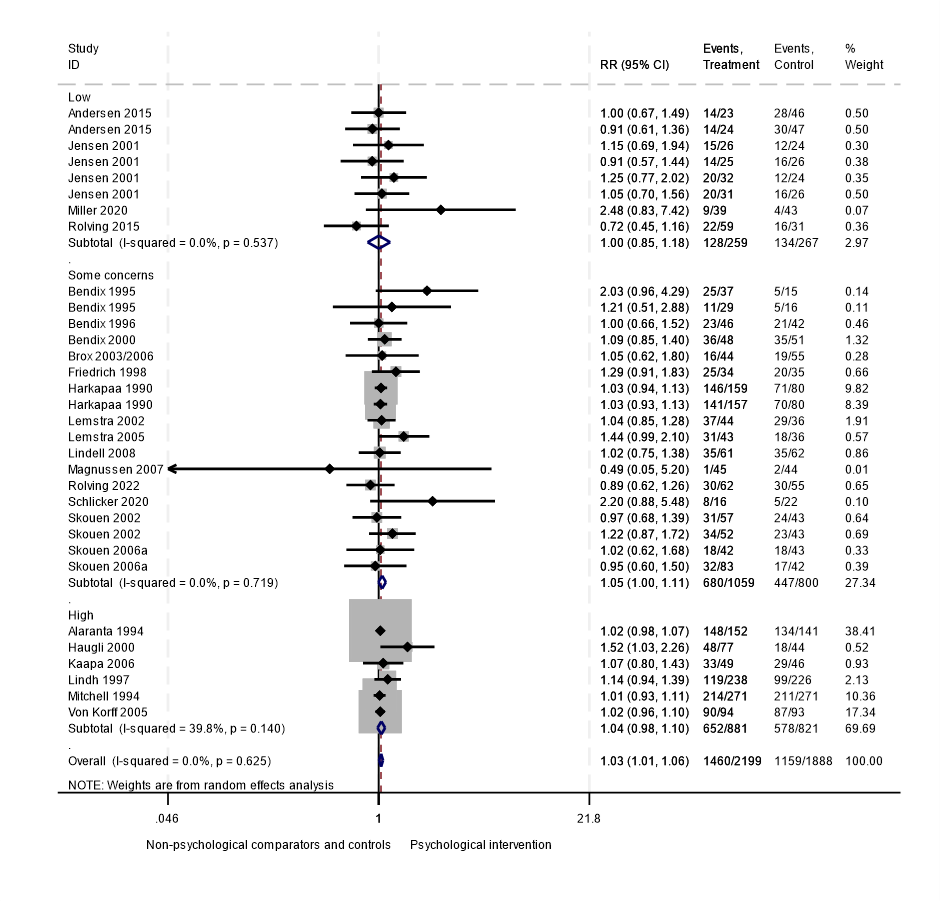


Supplementary file 13: meta-regression results for intervention delivery (all outcomes)

| **Who was involved in intervention delivery** | **Component estimate (RR / SMD) (95% CI), p-value** | | | |
| --- | --- | --- | --- | --- |
|  | **Work status (RR)** | **Return to work (RR)** | **Sick leave (SMD)** | **Work capacity (SMD)** |
| Psychology professional | 0.91 (0.73 to 1.14), p=0.40 | 0.97 (0.70 to 1.33), p=0.82 | 0.27 (-0.30 to 0.84), p=0.33 | -0.10 (-0.34 to 0.14), p=0.38 |
| Physiotherapist | 1.02 (0.77 to 1.35), p=0.90 | 0.97 (0.70 to 1.33), p=0.82 | -0.21 (-0.82 to 0.40), p=0.47 | -0.01 (-0.24 to 0.22), p=0.91 |
| Occupational therapist | 0.99 (0.93 to 1.05), p=0.76 | 0.91 (0.75 to 1.10), p=0.30 | -0.41 (-0.93 to 0.11), p=0.11 | -0.17 (-0.57 to 0.23), p=0.38 |
| Social worker | 0.99 (0.93 to 1.05), p=0.71 | 0.91 (0.74 to 1.12), p=0.32 | 0.001 (-0.60 to 0.60), p=1.00 | 0.19 (-0.18 to 0.56), p=0.28 |
| Person with lived experience | 0.85 (0.66 to 1.10), p=0.22 | 0.96 (0.70 to 1.30), p=0.75 | 0.43 (-0.68 to 1.54), p=0.43 | 0.05 (-0.35 to 0.46), p=0.78 |
| Health professional | 0.99 (0.92 to 1.07), p=0.77 | 1.11 (0.91 to 1.36), p=0.27* | 0.16 (-0.41 to 0.72), p=0.56 | 0.01 (-0.22 to 0.54), p=0.90 |
| Other | 1.00 (0.94 to 1.07), p=0.99 | 0.95 (0.79 to 1.15), p=0.59 | 0.09 9-0.70 to 0.89), p=0.80 | 0.32 (-0.16 to 0.80), p=0.17 |

Supplementary file 14: Intervention delivery mode in relation to work status

Supplementary file 15: Comparison of interventions containing a work component versus no work component in relation to work status

Supplementary file 16: Different follow-up time periods in relation to return to work

Supplementary file 17: Risk of bias in relation to return to work

Supplementary file 18: Intervention delivery mode in relation to return to work

Supplementary file 19: Comparison of interventions containing a work component versus no work component in relation to return to work

Supplementary file 20: Comparisons of interventions and comparator/controls in relation to sick leave at different follow-up time points

Supplementary file 21: Intervention delivery mode in relation to sick leave

Supplementary file 22: Risk of bias in relation to sick leave

Supplementary file 23: Comparison of interventions containing a work component versus those that don’t in relation to sick leave

Supplementary file 24: Sick leave sensitivity analysis for Johansson et al (1998) paper


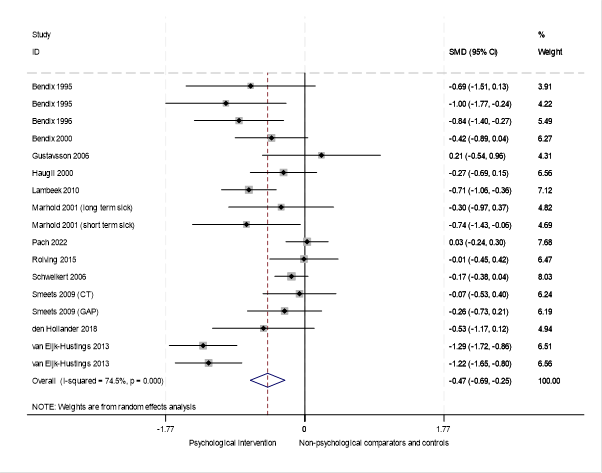


SMD -0.47 (95% CI -0.69 to -0.25). I2=74.5%.

Supplementary file 25: Interventions and comparator/controls in relation to work ability and work capacity at different follow-up time points


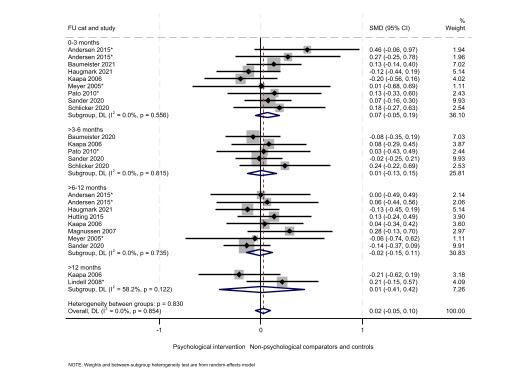


Supplementary file 26: Risk of bias in relation to work ability/capacity


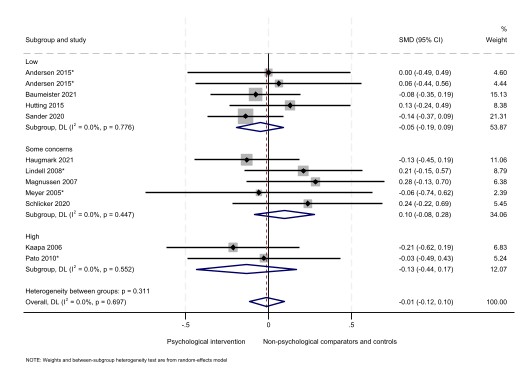


Supplementary file 27: Intervention delivery mode for work ability/capacity


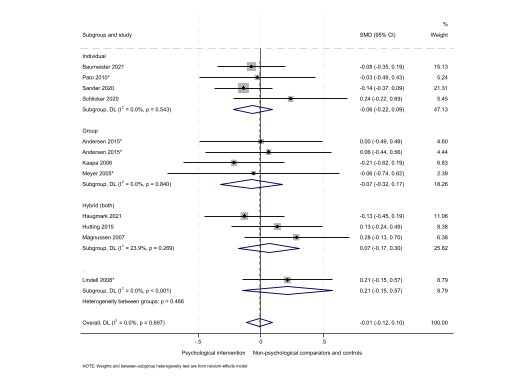


4= missing delivery mode data for Lindell 2008

Supplementary file 28: Comparison of interventions containing a work component versus those that don’t in relation to work ability and capacity

Supplementary file 29: RCT Narrative synthesis of work outcomes

| **Study** | **Outcome and timepoint** | **Result** | **Direction of effect** | **Overall ROB** |
| --- | --- | --- | --- | --- |
| Hutting 2015 | Self-efficacy at work up to 12 month follow up (higher = better) | B 2.32 (95% CI -2.55 to 0.17), p=0.11 | Intervention = control | Low |
| Hutting 2015 | Stanford Presenteeism Scale up to 12 month follow up (lower = better) | B 0.12 (95% CI -0.81 to 1.05), p=0.8 (adjusted for duration of symptoms and gender) | Intervention = control | Low |
| Lindell 2008 | Return to work chance at 6 months | HR 0.9 (95% CI 0.6 to 1.4), p>0.05 | Intervention = control | ? |
| Lindell 2008 | Return to work chance at 12 months | HR 1.2 (95% CI 0.7 to 2.0), p>0.05 | Intervention = control | ? |
| Lindell 2008 | Return to work chance at 18 months | HR 1.6 (95% CI 0.7 to 3.6), p>0.05 | Intervention = control | Some concerns |
| McKnight 2010 | ERGOS work simulator at 24 months | Cohen’s d between group effect sizes:  ST + SM vs ST -0.01 ST + SM vs SM -0.01 ST vs SM 0.00 | Intervention = control | High |

Supplementary file 30: Narrative synthesis: RCT psychological interventions versus psychological interventions

| Study | Intervention comparisons | Outcome and result | Direction of effect | Overall ROB |
| --- | --- | --- | --- | --- |
| Altmaier 1992 | 1. Mixed psychological including cognitive behavioural coping skills and counselling, plus education (mechanisms of pain), exercise, medical treatment 2. Counselling, plus education (mechanisms of pain), exercise, medical treatment | - RTW at 6 months follow up (fully employed at same/equivalent job) 11/24 (48%) vs 14/21 (67%) - RTW at 6 months follow up (part time, light duty or retraining) 18/24 (76%) vs 18/21 (86%) | No significant difference between interventions  No significant difference between interventions | High |
| Brendbekken 2017 | 1. Counselling and Physical Examination 2. Mixed psychological (eg, Counselling) plus Physical Examination | - Full RTW at 1 month follow up   RR 1.07 (0.44 to 2.56)   - Full RTW at 2 month follow up   RR 1.07 (0.53 to 2.18)   - Full RTW at 3 month follow up   RR 1.15 (0.61 to 2.18)   - Full RTW at 4 month follow up   RR 1.13 (0.62 to 2.03)   - Full RTW at 5 month follow up   RR 0.92 (0.53 to 1.60)   - Full RTW at 6 month follow up   RR 0.85 (0.50 to 1.45)   - Full RTW at 7 month follow up   RR 1.11 (0.66 to 1.87)   - Full RTW at 8 month follow up   RR 0.98 (0.59 to 1.64)   - Full RTW at 9 month follow up   RR 0.93 (0.57 to 1.54)   - Full RTW at 10 month follow up   RR 1.18 (0.72 to 1.95)   - Full RTW at 11 month follow up   RR 1.27 (0.78 to 2.09)   - Full RTW at 12 month follow up   RR 1.10 (0.67 to 1.81)   - Full RTW at 24 month follow up   RR 1.25 (0.75 to 2.06)   - Full RTW at 1 month follow up   RR 1.45 (0.88 to 2.39)   - Full RTW at 2 month follow up   RR 1.86 (1.10 to 3.14)   - Full RTW at 3 month follow up   RR 2.24 (1.28 to 3.91)   - Full RTW at 4 month follow up   RR 1.53 (0.87 to 2.68)   - Full RTW at 5 month follow up   RR 1.26 (0.70 to 2.28)   - Full RTW at 6 month follow up   RR 1.40 (0.75 to 2.61)   - Full RTW at 7 month follow up   RR 2.31 (1.19 to 4.51)   - Full RTW at 8 month follow up   RR 1.90 (0.97 to 3.72)   - Full RTW at 9 month follow up   RR 1.61 (0.77 to 3.72)   - Full RTW at 10 month follow up   RR 1.62 (0.75 to 3.53)   - Full RTW at 11 month follow up   RR 1.60 (0.74 to 3.46)   - Full RTW at 12 month follow up   RR 1.60 (0.74 to 3.46)   - Full RTW at 24 month follow up   RR 0.85 (0.42 to 1.71)   - Out of work at 12 month follow up 59/141 (41.8%) vs 65/143 (45.5%) - Out of work at 24 month follow up 63/141 (44.7%) vs 68/143 (47.6%) | No significant difference between interventions  No significant difference between interventions  No significant difference between interventions  No significant difference between interventions  No significant difference between interventions  No significant difference between interventions  No significant difference between interventions  No significant difference between interventions  No significant difference between interventions  No significant difference between interventions  No significant difference between interventions  No significant difference between interventions  No significant difference between interventions  No significant difference between interventions  Significant difference between interventions  Significant difference between interventions No significant difference between interventions  No significant difference between interventions  No significant difference between interventions  Significant difference between interventions  No significant difference between interventions  No significant difference between interventions  No significant difference between interventions  No significant difference between interventions  No significant difference between interventions  No significant difference between interventions  No significant difference between interventions  No significant difference between interventions | Low |
| Calner 2017 | 1. mixed psychological (eg cognitive behavioural counselling, mindfulness, action planning) plus ergonomics, medical treatment, education, passive therapy and exercise. 2. mixed psychological (eg cognitive behavioural counselling, mindfulness) plus ergonomics, medical treatment, education, passive therapy. | - Work status at 4 month follow up   Not working: 21/55 (38%) vs 15/44 (34%) Working 25%: 2/55 (4%) vs 2/44 (5%) Working 50%: 9/55 (16%) vs 8/44 (18%) Working 75%: 1/55 (2%) vs 0/44 (0%) Working 100%: 22/55 (40%) vs 19/44 (43%)   - Work status at 12 month follow up Not working: 19/53 (36%) vs 11/44 (43%) Working 25%: 2/53 (36%) vs 1/44 (2%) Working 50%: 6/53 (11%) vs 10/44 (23%) Working 75%: 3/53 (6%) vs 3/44 (7%) Working 100%: 23/53 (43%) vs 19/44 (43%) - Work ability index at 4 month follow up  Adjusted treatment effect 1.1 (95% CI -2.2 to 4.3), p=0.52 - Work ability index at 12 month follow up Adjusted treatment effect -0.1 (95% CI -4.6 to 4.3), p=0.95 - Work ability index score at 4 month follow up Adjusted treatment effect -0.5 (95% CI -1.4 to 0.4), p=0.30 - Work ability index score at 12 month follow up Adjusted treatment effect -0.4 (95% CI -1.4 to 0.7), p=0.47 | No significant difference between interventions  No significant difference between interventions  No significant difference between interventions  No significant difference between interventions  No significant difference between interventions  No significant difference between interventions | Some concerns |
| Ehrenborg 2010 | 1. conventional interdisciplinary programme without Biofeedback - Mixed psychology (psychoeducation, relaxation, stress management) plus exercise and ergonomic 2. conventional interdisciplinary programme with Biofeedback - Mixed psychology (biofeedback, psychoeducation, relaxation, stress management) plus exercise and ergonomic | - Work status at 6 month follow up (working part time or more) 18 (72%) vs 25 (76%) - Work status at 6 month follow up (full-time workers) 8 (32%) vs 15 (52%) | No significant difference between interventions  Significant difference between interventions | Some concerns |
| Hampel 2019 | 1. mixed psychological (e.g. psychoeducation, cognitive-behavioural exercises) 2. mixed psychological (e.g. psychoeducation, cognitive-behavioural exercises and cog behavioural depression prevention) | - Pain-related sick leave up to 12 months follow-up  Freidman’s ANOVA IG2 χ^2^(2)=76.55, p<0.001 Freidman’s ANOVA IG1 χ^2^(2)=22.92, p=0.002 - Work ability index (total) up to 12 months follow up Repeated measures ANOVA  Treatment group p=0.92 Time p<0.001 Treatment group x time p=0.001 - Work ability index (total) up to 24 month follow up Repeated measures ANOVA Treatment group p=0.43 Time p<0.001 Treatment group x time p=0.006 - Work ability index (mental) up to 12 months follow up Treatment group p=0.57 Time p<0.001 Treatment group x time p<0.001 - Work ability index (physical) up to 12 months follow up   Treatment group p=0.64 Time p<0.001 Treatment group x time p=0.08 | No significant difference between interventions (but effect sizes in IG2 were larger)  No significant difference between interventions (treatment group main effect)  Significant difference between interventions (treatment group x time interaction)  No significant difference between interventions (treatment group main effect)  Significant difference between interventions (treatment group x time interaction)  No significant difference between interventions (treatment group main effect)  Significant difference between interventions (treatment group x time interaction)  No significant difference between interventions (treatment group main effect) | High  High |
| Jensen 1997 | 1. CBT plus exercise 2. CBT plus exercise and additional psychological component (including cognitive restructuring) | - Sick leave up to 18 month follow up: “no significant differences were obtained between groups either pre- or posttreatment.” | No significant difference between interventions | Low |
| Kool 2005 | 1. counselling, plus ergonomics, exercise, medical treatment 2. relaxation (progressive muscle relaxation), exercise, passive therapy, medical treatment | - Number at work at 3 months follow up 40/86 vs 24/87, p=0.04 - Unemployment rate at 3 months follow up 16/86 vs 21/87, p=0.64 - Unemployment rate at 1 year follow up 37/87 vs 45/87, p=0.23 - Returned to work at 1 year  52/87 vs 36/87, p=0.02; OR 2.11 (95% CI 1.15 to 3.85) - Received permanent disability allowance (full or partial) at 1 year 32/87 vs 38/87, p=0.20 - Working capacity at 3 weeks Not fit to work: 7/87 vs 15/87 100% fit to work: 42/87 vs 32/87,   50% fit to work 35/87 vs 34/87, p=0.07   - Days at work at 3 month follow up 25.9 (32.2) vs 15.8 (27.5), p=0.03 - Number of work days at 1 year follow up 118 (134) vs 74 (114), p=0.01 | Significant difference between interventions  No significant difference between interventions  No significant difference between interventions  Significant difference between interventions  No significant difference between interventions  No significant difference between interventions  Significant difference between interventions  Significant difference between interventions | Some concerns |
| Reme 2016 | 1. Brief Cognitive Intervention (BI) - (eg, coping skills -NOT CBT) plus education 2. (BI and CBT) - Mixed psychological (eg, CBT) plus education 3. (BI and Group CBT) - Mixed psychological (eg, CBT) plus education 4. (BI and PE) - cognitive intervention (eg, coping skills -NOT CBT) plus Exercise, education. 5. (BI plus seal oil) - Brief Cognitive Intervention (BI) - (eg, coping skills -NOT CBT) plus education, seal oil. 6. (BI plus soy oil) - Brief Cognitive Intervention (BI) - (eg, coping skills -NOT CBT) plus education, soy oil. | *Results only reported for IGs 1,2,5,6*   - Reduced sick leave (part/full RTW) at 1 month follow up 36/100 vs 18/102 vs 14/105 vs 23/105, p<0.01 - Reduced sick leave (part/full RTW) at 2 month follow up 49/100 vs 40/102 vs 31/105 vs 38/105, p=0.04 - Reduced sick leave (part/full RTW) at 3 month follow up 60/100 vs 47/102 vs 43/105 vs 44/105, p=0.02 - Reduced sick leave (part/full RTW) at 4 month follow up 64/100 vs 49/102 vs 51/105 vs 52/105, p=0.06 - Reduced sick leave (part/full RTW) at 5 month follow up 63/100 vs 59/102 vs 54/105 vs 56/105, p=0.40 - Reduced sick leave (part/full RTW) at 6 month follow up 61/100 vs 61/102 vs 58/105 vs 56/105, p=0.67 - Reduced sick leave (part/full RTW) at 7 month follow up 58/100 vs 60/102 vs 58/105 vs 56/105, p=0.81 - Reduced sick leave (part/full RTW) at 8 month follow up 58/100 vs 63/102 vs 55/105 vs 57/105, p=0.59 - Reduced sick leave (part/full RTW) at 9 month follow up 53/100 vs 61/102 vs 55/105 vs 57/105, p=0.70 - Reduced sick leave (part/full RTW) at 10 month follow up 57/100 vs 55/102 vs 55/105 vs 57/105, p=0.92 - Reduced sick leave (part/full RTW) at 11 month follow up 59/100 vs 55/102 vs 57/105 vs 53/105, p=0.63 - Reduced sick leave (part/full RTW) at 12 month follow up 60/100 vs 51/102 vs 54/105 vs 56/105, p=0.47 - Full RTW only at 12 month follow up 56/100 vs 48/102 vs 54/105 vs 50/105, p>0.05 | Significant difference between interventions  Significant difference between interventions  Significant difference between interventions  No significant difference between interventions  No significant difference between interventions  No significant difference between interventions  No significant difference between interventions  No significant difference between interventions  No significant difference between interventions  No significant difference between interventions  No significant difference between interventions  No significant difference between interventions  No significant difference between interventions | Low |

Supplementary file 31: Sensitivity analysis including additional papers

Sensitivity analysis – including the results of Cui 2023 and Hansen 2023

Work capacity meta-analysis

SMD -0.02 (95% CI -0.12 to 0.08).

No difference to the results.
